# Cobalt‐Catalyzed Suzuki Biaryl Coupling of Aryl Halides

**DOI:** 10.1002/anie.201710053

**Published:** 2017-11-28

**Authors:** Soneela Asghar, Sanita B. Tailor, David Elorriaga, Robin B. Bedford

**Affiliations:** ^1^ School of Chemistry University of Bristol Cantock's Close Bristol BS8 1TS UK; ^2^ Department of Chemistry and Chemical Engineering SBA School of Science & Engineering Lahore University of Management Sciences Lahore 54792 Pakistan

**Keywords:** aryl halides, carbenes, cobalt, N-heterocycles, Suzuki coupling

## Abstract

Readily accessed cobalt pre‐catalysts with N‐heterocyclic carbene ligands catalyze the Suzuki cross‐coupling of aryl chlorides and bromides with alkyllithium‐activated arylboronic pinacolate esters. Preliminary mechanistic studies indicate that the cobalt species is reduced to Co^0^ during the reaction.

The palladium‐catalyzed cross‐coupling of organoboronic acids or esters with organic halides or related substrates—the Suzuki reaction—is a powerful and very widely exploited method for the formation of biaryl compounds (Scheme 1).^1^ This reaction is used commercially for the production of a range of materials, including the sartan class of angiotensin inhibitors for the treatment of hypertension (e.g. Lorsartan) and Boscalid, a broad‐spectrum agrochemical fungicide.^2^

**Scheme 1:**
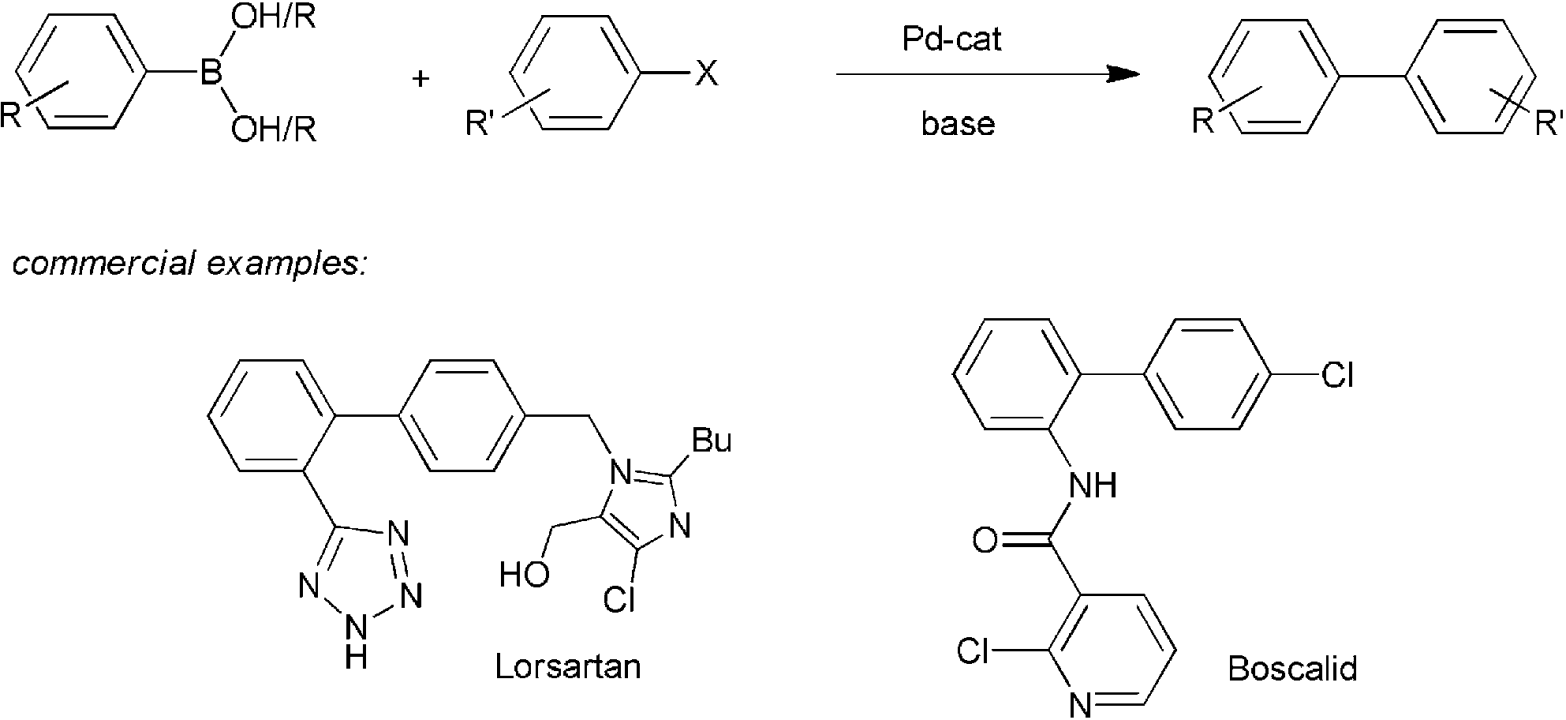
Palladium‐catalyzed Suzuki biaryl coupling and selected products.

While palladium‐based catalysts are ubiquitous in Suzuki cross‐coupling reactions, there is a growing impetus to replace them with more sustainable alternatives based on earth‐abundant metals (EAM). This is not only because of the fact that palladium, like all platinum‐group metals (PGMs), is scarce and expensive, but also because of the relatively high toxicity of PGMs; as a consequence of this toxicity there are strict regulations in place for the removal of PGMs to the low ppm level from active pharmaceutical intermediates.^3^ Of the EAM alternatives to palladium for Suzuki biaryl coupling, first‐row transition metals are particularly attractive, with nickel‐based catalysts being the most well developed to date.^4^, ^5^ Iron‐catalyzed Suzuki cross‐coupling can be performed between a variety of organic halides and organoboron reagents,^6^ but simple biaryl bond formation remains elusive and challenging.6a, 7, 8 Recently, Chirik and co‐workers reported early results in the coupling of aryl triflates with arylboron pinacol esters by using a cobalt PNP‐pincer‐based catalyst.^9^ We now report the cross‐coupling of aryl chlorides and bromides with activated arylboronic pinacol esters,^10^, ^11^ using simple cobalt catalysts prepared in situ from commercially available precursors.

The results shown in Table 1 summarize selected^12^ optimization studies using 4‐chorotoluene and the activated phenylboronic ester **1 a**,6b,6c,6e, 8 catalyzed by species formed in situ from cobalt(II) chloride with a range of different ligands and ligand precursors.

**Table 1:** Optimization of ligands. 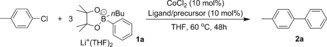

Entry	Ligand/ precursor	Yield [%]^[a]^	Entry	Ligand/ precursor	Yield [%]^[a]^
1	none	0	8	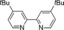	50
2	PPh_3_	2	9	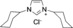	24
3	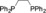	6	10	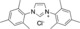	92
4	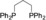	0	11	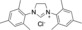	71
5	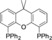	8	12	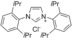	99
6	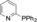	0	13	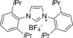	99
7	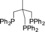	0	14	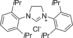	99

[a] Yield of **2 a**, determined by ^1^H NMR spectroscopy (1,3,5‐trimethoxybenzene as an internal standard).

No reaction was observed in the absence of added ligand (Table 1, entry 1) and little or no product **2 a** was obtained when mono, bi‐, or tridentate phosphine ligands were tested (Table 1, entries 3–7). Modest conversion was obtained with a bipyridyl ligand architecture (Table 1, entry 8) and some activity was also observed with an *N*,*N*′‐dialkyl N‐heterocyclic carbene (NHC) precursor (Table 1, entry 9). However, the star performers proved to be *N*,*N*′‐diaryl‐substituted NHC precursors (Table 1, entries 10–14) with IPr (Table 1, entries 12 and 13) and SIPr (Table 1, entry 14) precursors giving essentially quantitative conversion to the desired product at 60 °C; reducing either the temperature or the loading of **1 a** proved deleterious to the performance.^12^ Importantly, a repeat of the reaction outlined in entry 10 gave no product when the CoCl_2_ was omitted, whereas repeating the reaction in entry 14 with high purity CoCl_2_ (99.999%) gave **2 a** in essentially quantitative yield. This finding indicates that impurities, in either the cobalt source or one of the other components of the reaction mixture, are not responsible for the observed catalytic activity.^7^

The effect of changing either the halide or the boronic ester activator (RM) is summarized in Scheme 2. Under these conditions, *t*BuLi works as well as *n*BuLi, whereas the Grignard reagent EtMgCl performs poorly, meanwhile no reaction is seen with lithium isopropoxide. Interestingly, poorer activity results from the use of 4‐bromotoluene as a substrate compared with the use of its chloride congener. This is in stark contrast to palladium‐catalyzed cross‐coupling, in which the weaker aryl bromide bonds are cleaved more readily. This finding may indicate a significant deviation in the mechanism of activation of the C−X bond for palladium and cobalt species (see below). Very poor results were obtained with 4‐iodotoluene as the electrophile.

**Scheme 2:**
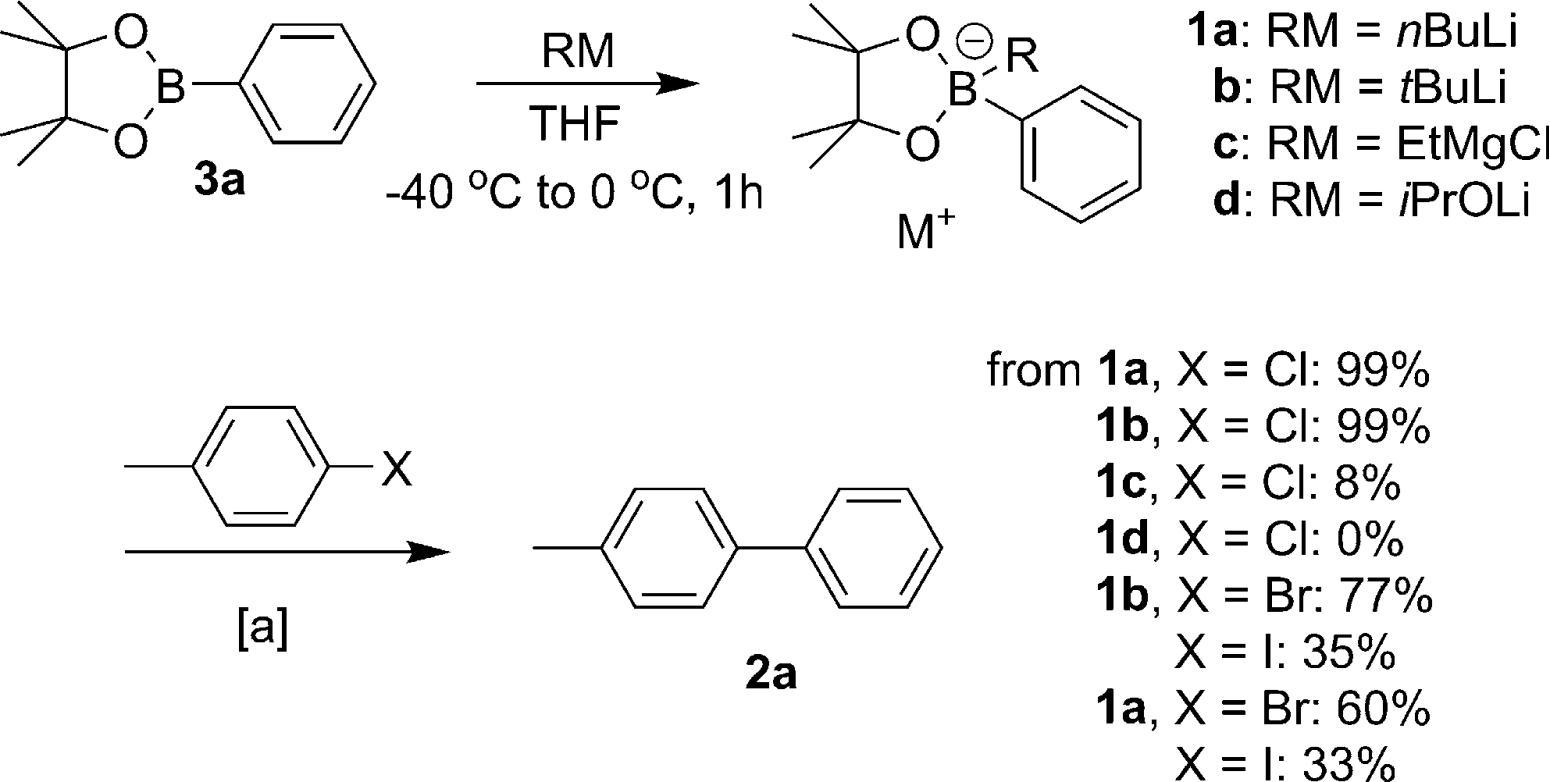
Varying the boronic ester activator (RM) and 4‐tolyl halide. [a] Conditions as per Table 1, entry 14.

The results obtained under the optimized conditions in the cross‐coupling of a variety of aryl chlorides and bromides with *n*BuLi‐activated aryl pinacol boronic esters are summarized in Table 2. A range of electron‐rich and electron‐poor aryl halides were tolerated, with good‐to‐excellent yields of the desired cross‐coupled products obtained. Moderate steric hindrance in the aryl halide substrate was also well accommodated (Table 2, entries 13–16 and 21). Esters, tertiary amines, amides, fluoro, trifluoromethyl, and alkoxide functions were tolerated on the aryl halide. By contrast, nitro, cyano, aldehydic, and ketonic substituents on the aryl halide groups were poorly tolerated, giving either little or none of the desired product, or substantial amounts of products from competitive side reactions (see Chart S1 in the Supporting Information).^12^ The yields of the isolated products from the heterocyclic halides screened in Table 2, entries 25–27, and Chart S1 were somewhat disappointing, owing largely to poor separation of the products from biphenyl, produced by competitive homo‐coupling of the electrophile.

**Table 2:** Reaction scope. See Chart S1 in the Supporting Information for unsuccessful attempts. 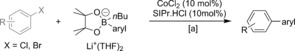

Entry^[a]^	Aryl halide	Product	Yield [%]^[b]^
1	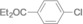	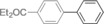	88
2	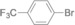	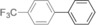	67
3	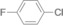	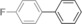	93
4	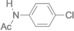	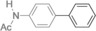	75
5	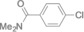	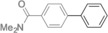	69
6	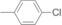	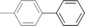	92
7	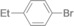	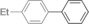	69
8	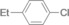	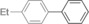	79
9	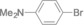	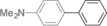	80
10	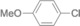	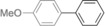	82
11	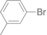	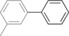	78
12	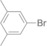	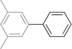	84
13	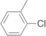	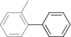	85
14	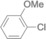	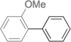	86
15	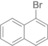	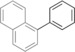	73
16	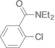	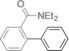	55
17	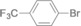	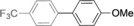	82
18	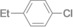	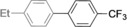	76
19	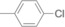	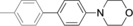	73
20	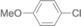	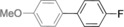	82
21	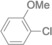	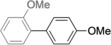	78
22	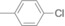	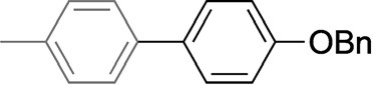	82
23	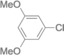	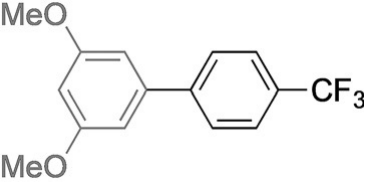	85
24	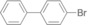	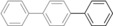	58
25	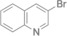	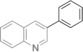	37
26	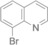	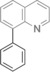	35
27	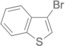	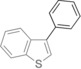	33

[a] Conditions as per Table 1. [b] Yields of isolated products.

The synthetic utility of the transformation was highlighted by functionalization of the biologically relevant, heterocycle‐containing substrate Edaravone, a substance used for the treatment of brain ischemia following a stroke.^13^ The reaction of the commercially available *ortho*‐chloro‐substituted analogue of Edaravone, **4 a**, with activated arylBPin esters is outlined in Scheme 3. Gratifyingly, the 2‐arylated Edaravone derivatives **5 a**–**g** could be produced with moderate‐to‐good yields of isolated products. Surprisingly, the *meta*‐ and *para*‐chloro analogues of Edaravone, **4 b** and **c**, respectively, did not undergo any coupling. This result suggests that the dihydro‐*3H*‐pyrazol‐3‐one moiety strongly directs to the *ortho*‐position; such a directing effect has previously been exploited in palladium‐catalyzed *ortho*‐C−H functionalization of Edaravone.^14^ Indeed, in stark contrast to the reactions with simple aryl halides, the coupling of **4 a** with **1 a** proceeded equally well in the absence of the NHC ligand, giving **5 a** in 86 % yield, determined by ^1^H NMR spectroscopy, which is consistent with a strong *ortho*‐directing effect.^12^

**Scheme 3:**
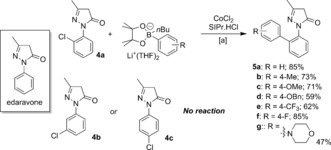
Suzuki coupling of an Edaravone derivative, **4 a**. [a] Conditions as per Table 1; yields of isolated products are shown.

Finally, we turned our attention to preliminary mechanistic investigations. To determine the lowest oxidation state that could be accessed by the Co species in the presence of the reducing organoborate, we reacted **1 a** with CoCl_2_ and free SIPr, exploiting the diene dvtms (1,3‐divinyl‐1,1,3,3‐tetramethyldisiloxane) as a trap for any low‐valent, low‐coordinate organocobalt intermediates obtained (Scheme 4). Following this reaction by ^1^H NMR spectroscopy,^12^, ^15^ we observed the production of the zero‐valent cobalt complex **6 a**, which could be more readily prepared and isolated by using potassium graphite as the reducing reagent, by extension of a methodology reported by Deng and co‐workers for a similar complex.^16^ This synthetic approach could also be exploited to produce the bis‐norbornene analogue, **6 b**. The single‐crystal X‐ray structures of the complexes **6 a** and **b** are shown in Figure 1.

**Figure 1:**
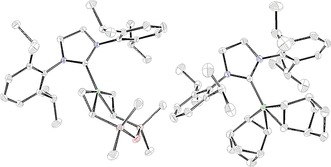
Single‐crystal X‐ray structures of zero‐valent cobalt complexes **6 a** (left) and **6 b** (right). Ellipsoids set at 50 % probability, Hydrogen atoms omitted for clarity.

**Scheme 4:**
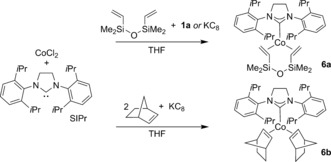
Reduction of the cobalt species in the presence of alkenes.

The diene complex **6 a** showed no activity when trialed as a pre‐catalyst in the coupling of 4‐chlorotoluene with **1 a**. Indeed, the diene acts as a poison for the catalytic reaction, with no **2 a** produced when the reaction using CoCl_2_/SIPr⋅HCl was repeated in its presence.^12^ This lends credence to the tentative suggestion that cobalt(0) species are formed during catalysis. By contrast, when the bis‐norbornene complex **6 b** was exploited as the pre‐catalyst good activity was observed, with **2 a** produced in 60 % yield, as determined by ^1^H NMR spectroscopy. If the reaction does indeed involve a Co^0^ intermediate, then subsequent reaction with the aryl halide may proceed via either a classical two‐electron oxidative addition or, more likely, a bimetallic, radical‐centered process.^17^ Certainly, the observed preference for aryl chlorides over bromides outlined above is atypical of simple, mononuclear oxidative addition.^18^

In summary, we have developed the cobalt‐catalyzed Suzuki cross‐coupling of aryl chlorides and bromides with activated arylBPin esters, using simple and commercially available ligands and cobalt salts. There are remaining issues for the routine replacement of palladium with cobalt in the Suzuki coupling of aryl halides, including the need to develop milder nucleophiles to prevent competitive side reactions, and to reduce catalyst loadings; these studies are ongoing within the group. Regardless, we have unequivocally demonstrated that cobalt‐catalyzed Suzuki biaryl bond formation by using aryl halide substrates is tractable.

## Conflict of interest

The authors declare no conflict of interest.

## Supporting information

As a service to our authors and readers, this journal provides supporting information supplied by the authors. Such materials are peer reviewed and may be re‐organized for online delivery, but are not copy‐edited or typeset. Technical support issues arising from supporting information (other than missing files) should be addressed to the authors.

Supplementary
