# Supplementary material for: Cobalt‐Catalyzed Suzuki Biaryl Coupling of Aryl Halides
Source: Angew Chem Int Ed Engl. 2017 Nov 28;56(51):16367–70. doi: 10.1002/anie.201710053 (PMC5767760; doi:10.1002/anie.201710053)
Supplement: Supplementary file 1 — Supplementary [file ANIE-56-16367-s001.pdf]

Angewandte Chemie
Eine Zeitschrift der Gesellschaft Deutscher Chemiker

Supporting Information

Cobalt-Catalyzed Suzuki Biaryl Coupling of Aryl Halides

*Soneela Asghar, Sanita B. Tailor, David Elorriaga, and Robin B. Bedford**

anie_201710053_sm_miscellaneous_information.pdf

Supporting Information

Table of Contents

1. General Information S2
2. Experimental Details S2
   2.1 Preparation of activated boronate nucleophiles, Li[(Ar)(^{n}Bu)Bpin], **1**. S2
   2.2 Optimisation of the Reaction Conditions S2
   2.3 General Procedure for Cobalt-Catalyzed Suzuki Cross-Coupling of Aryl Halides S4
   2.4 Competition reaction S5
   2.5 Data for Catalysis Products S5
   2.6 Unsuccessful Reactions S17
   2.7 Preparation and Reactivity of Cobalt (0) Complexes S18
   2.8 ^{1}H and ^{13}C NMR Spectra of Cross-Coupled Products S23
3. Crystallographic Data S57
4. References S58

1. General Information

Unless otherwise indicated, all experiments were performed under a dry and inert atmosphere (N_2 or Ar) using standard Schlenk-line and glovebox techniques. Anhydrous solvents were obtained using an

Anhydrous Engineering double alumina column drying system. Commercial grade solvents were used for chromatography and work-up procedures. Column chromatography was performed using technical grade silica gel, pore size 60 Å, 230-400 mesh particle size. TLC analyses were performed using aluminium plates covered with SiO_2 (Merck 60, F-254) and visualized by UV detection (254 or 365 nm). Benzene-d_6 was degassed and dried over activated molecular sieves (4 Å). 1,3-Bis(2,6-di-*iso*-propylphenyl)imidazolidin-2-ylidene (SIPr) was synthesised according to literature procedure.[S1] ^{1}H, ^{13}C and ^{19}F NMR spectra were recorded in $CDCl_3$ on Varian 400-MR, Bruker Nano 400, Jeol ECS 400 or Bruker Advance III HD 500 Cryo spectrometers. Chemical shifts (δ) are reported in parts per million (ppm) referenced to the solvent residual peak. Coupling constants (J) are given in Hz and multiplicities being abbreviated as: br (broad), s (singlet), d (doublet), t (triplet), q (quartet), m (multiplet). Infrared spectra were recorded using a Perkin Elmer Spectrum Two FT-IR spectrometer. Absorption spectra were recorded with an Ocean Optics USB2000+UV-VIS spectrometer. The method for determining magnetic moments originally described by Evans was followed. [S2] Mass spectrometry was performed by the University of Bristol mass spectrometry service by electrospray ionisation (ESI^+) using a Bruker Daltonics MicroTOF II. GC-FID/MS analysis was conducted using an Agilent 7820A GC system and 5977B MSD.

2. Experimental Details

2.1 Preparation of activated boronate nucleophiles, Li[(Ar)(^{n}Bu)Bpin], 1.

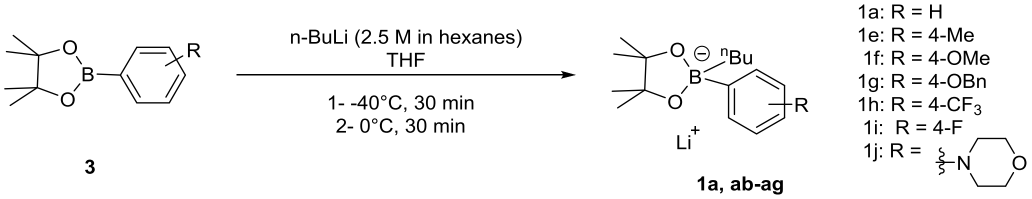

3

1a, ab-ag

1a: R = H
1e: R = 4-Me
1f: R = 4-OMe
1g: R = 4-OBn
1h: R = 4-CF_3
1i: R = 4-F
1j: R =

The appropriate arylboronic acid pinacol ester, **3**, (1.5 mmol) was dissolved in THF (3.8 ml) in a Schlenk flask. To this, was added a solution of 2.5 M *n*-butyl lithium in hexanes (1,5 mmol) in one portion at -40 °C. The mixture was stirred at -40 °C for 30 minutes, allowed to warm to 0 °C and then stirred for another 30 minutes. This resultant solution of **1** was used immediately after preparation. [S3]

2.2 Optimisation of the Reaction Conditions

A Schlenk flask was charged with the appropriate amount of $CoCl_2$ (2 – 10 mol%) SIPr·HCl (2 – 10 mol%) and dry THF (1 ml) was added. The solution was stirred at room temperature for 1 hour. A solution of the appropriate borate, **1** (0.5 – 0.75 mmol) and then chlorotoluene (30 µl, 0.25 mmol) were added, the mixture was stirred at the appropriate temperature for 12-48 h, and then cooled to room temperature. The reaction mixture was quenched with $HCl_{(aq)}$ (1 M, 2 ml) and the organics were extracted using CH_2Cl_2 (3 x 10 ml) and dried over anhydrous Na_2SO_4. The organic extracts were

filtered and the solvent removed under reduced pressure to afford the crude product, **2a**. The product was dissolved in $CDCl_3$, 1,3,5-trimethoxybenzene (42.0 mg, 0.25 mmol, internal standard) and the conversion to **2a** was determined by ^{1}H NMR spectroscopy. The variations to components or conditions summarised in tables 1 and S1 – S3.

Table S1. Optimisation of the borate nucleophile

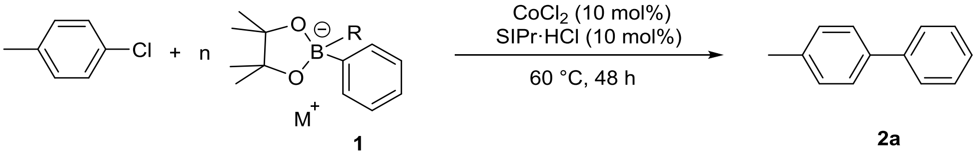

1 2a

Entry	R	M+	Borate (equiv)	Yield (%)[a]
1	^{t}Bu	Li	3	99
**2**	**^{n}Bu**	**Li**	**3**	**99**
3	Et	MgCl	3	8
4	^{i}PrO	Li	3	0
5	^{n}Bu	Li	2.5	74
6	^{n}Bu	Li	2	54

[a] Reaction conditions: 4-Chlorotoluene (0.25 mmol), **1** (0.75 mmol), $CoCl_2$ (0.025 mmol, 10 mol%), SIPr·HCl (0.025 mmol, 10 mol%), 60 °C, 48 h. Spectroscopic yield of **2a**, determined by ^{1}H NMR spectroscopy (1,3,5-trimethoxybenzene internal standard)

Table S2. Screening of cobalt sources

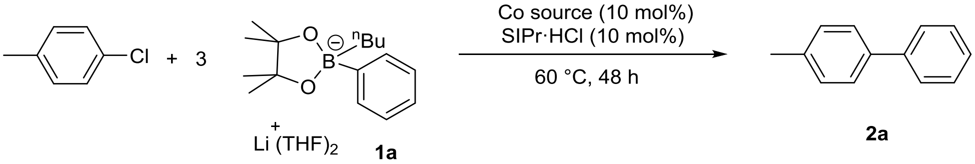

1a 2a

Entry	Cobalt source (% purity)	Yield (%)[a]
1	No cobalt	0
**2**	$CoCl_2$ (99.999%)	**99**
3	$CoBr_2$ (99%)	86
4	$Co(acac)_2$ (97%)	98
5	$Co(acac)_3$ (99.99)	98

[a] Reaction conditions: 4-Chlorotoluene (0.25 mmol), **1a** (0.75 mmol), Co source (0.025 mmol, 10 mol%), SIPr·HCl (0.025 mmol, 10mol %), 60 °C, 48 h. Spectroscopic yield of **2a**, determined by ^{1}H NMR spectroscopy (1,3,5-trimethoxybenzene internal standard)

Table S3. Screening of reaction parameters

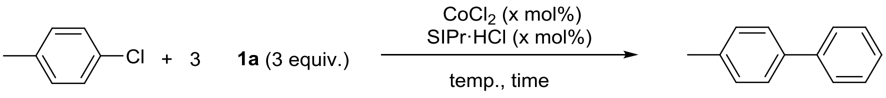

2a

Entry	x	Temp. (°C)	Time (h)	Yield (%)[a]
1	2	60	48	32
2	5	60	48	76
3	10	60	48	99
4	10	50	48	42
5	10	rt	48	0
6	10	60	24	92
7	10	60	12	82

[a] Reaction conditions: 4-Chlorotoluene (0.25 mmol), **1a** (0.75 mmol), $CoCl_2$ (0.025 mmol, 10 mol%), SIPr·HCl (0.025 mmol, 10 mol%), 60 °C, 48 h. Spectroscopic yield of **2a**, determined by ^{1}H NMR spectroscopy (1,3,5-trimethoxybenzene internal standard)

2.3 General Procedure for Cobalt-Catalyzed Suzuki Cross-Coupling of Aryl Halides

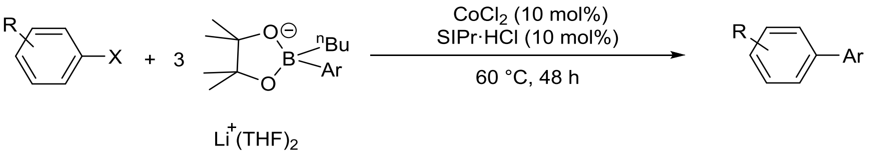

1a, ab-g

A Schlenk flask was charged with $CoCl_2$ (6.5 mg, 0.05 mmol, 10 mol%), SIPr·HCl (21.4 mg, 0.05 mmol, 10 mol%) and dry THF (1 ml) was added. The solution was stirred at room temperature for 1 hour. Freshly prepared borate **1** (1.5 mmol in THF 3.8 ml) and then aryl halide (0.5 mmol) were added, the mixture was stirred at 60 °C for 48 h and then cooled to room temperature. The reaction mixture was quenched with $HCl_{(aq)}$ (1 M, 2 ml) and the organics were extracted using CH_2Cl_2 (3 x 10 ml) and dried over anhydrous Na_2SO_4. The organic extracts were filtered and the solvent removed under reduced pressure to afford the crude product. Spectroscopic yields were determined by ^{1}H NMR spectroscopy (1,3,5-trimethoxybenzene internal standard). The crude product was purified by column chromatography (silica gel).

2.4 Competition reaction

Conducted according to the General Procedure for catalysis using 4-chlorotoluene (0.25 mmol) and 4-bromotoluene (0.25 mmol), **1a** (0.75 mmol), $CoCl_2$ (0.025 mmol), SIPr·HCl (0.025 mmol, 12 h. The ratio of the coupled products 4-methylbiphenyl and 4-ethylbiphenyl was determined as ~ 60:40 (0.185 mmol: 0.123 mmol) by ^{1}H NMR spectroscopy (1,3,5-triemethoxybenzene internal standard) with the combined conversion of ~62% to the cross-coupling products.

2.5 Data for Catalysis Products

Ethyl [1,1'-biphenyl]-4-carboxylate (Table 2, Entry 1)

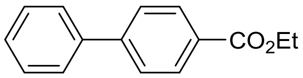

The general procedure was followed using ethyl 4-chlorobenzoate and **1a**. Purification by column chromatography (hexanes/CH_2Cl_2 2:1) gave the title compound as white solid (99.6 mg, 88%).
^{1}H NMR (500 MHz, $CDCl_3$) δ 8.12 (d, *J* = 8.7 Hz, 2H), 7.66 (d, *J* = 8.6 Hz, 2H), 7.63 (d, *J* = 7.0 Hz, 2H), 7.47 (t, *J* = 7.5 Hz, 2H), 7.40 (t, *J* = 7.3 Hz, 1H), 4.41 (q, *J* = 7.1 Hz, 2H), 1.42 (t, *J* = 7.1 Hz, 3H). ^{13}C NMR (126 MHz, $CDCl_3$) δ 166.69 (CO), 145.68 (C), 140.22 (C), 130.21(2CH), 129.40 (C), 129.07 (2CH), 128.25 (CH), 127.43 (2CH), 127.16 (2CH), 61.13 (CH_2), 14.52 (CH_3). Spectroscopic data in agreement with the literature. [S4]

4-(Trifluoromethyl)-1,1'-biphenyl (Table 2, Entry 2)

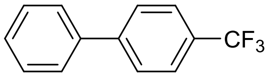

The general procedure was followed using ethyl 4-bromobenzotriflouride and **1a**. Purification by column chromatography (hexanes) gave the title compound as a white solid. (74 mg, 67%).
^{1}H NMR (400 MHz, $CDCl_3$) δ 7.70 (s, 4 H), 7.60 (d, *J* = 6.9 Hz, 2 H), 7.51 – 7.46 (m, *J* = 7.6 Hz, 2 H), 7.44 – 7.38 (m, *J* = 7.6 Hz, 1 H). ^{13}C NMR (126 MHz, $CDCl_3$) δ 144.88 (C), 139.92 (C), 129.49 (q, $^2J_{C\text{-}F}$ = 32.76 Hz, C), 129.13 (2 CH), 128.33 (CH), 127.57 (2 CH), 127.43 (2 CH), 125.85 (q, $^3J_{C\text{-}F}$ = 3.8 Hz, 2 CH), 124.45 (q, $^1J_{C\text{-}F}$ = 272.16 Hz, CF_3). ^{19}F NMR (377 MHz, $CDCl_3$): δ = -62.42 (CF_3). Spectroscopic data in agreement with the literature. [S5]

4-Fluoro-1,1'-biphenyl (Table 2, Entry 3)

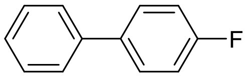

The general procedure was followed using ethyl 1-chloro-4-fluorobenzene and **1a**. Purification by column chromatography (hexanes) gave the title compound as a white solid. (80 mg, 93%).

^{1}H NMR (400 MHz, $CDCl_3$) δ 7.63 – 7.53 (m, 4H), 7.48 – 7.42 (m, 2H), 7.36 (t, J = 7.6 Hz, 1H), 7.14 (t, J = 8.7 Hz, 2H). ^{13}C NMR (101 MHz, $CDCl_3$) δ 162.61 (d, $^{1}J_{C-F}$ = 246.4 Hz, C), 141.39 (C), 140.41 (C), 128.96 (CH), 128.89 (CH), 128.88 (CH), 128.79 (CH), 127.35 (d, $^{3}J_{C-F}$ = 8.7 Hz, 2CH), 127.16 (CH), 115.75 (d, $^{2}J_{C-F}$ = 21.4 Hz, 2CH). ^{19}F NMR (377 MHz, $CDCl_3$): δ = -115.88 (F). Spectroscopic data in agreement with the literature. [S6]

***N*-([1,1'-Biphenyl]-4-yl)acetamide (Table 2, Entry 4)**

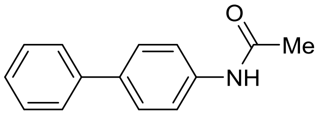

The general procedure was followed using 4-chloroacetanilide and **1a**. Purification by column chromatography (hexanes/ CH_2Cl_2 1:2) gave the title compound as a white solid (79 mg, 75%).

^{1}H NMR (400 MHz, $CDCl_3$) δ 7.62 (s, 1H), 7.41 (d, J = 8.0 Hz, 2H), 7.34 (d, J = 1.7 Hz, 2H), 7.32 (t, J = 1.4 Hz, 1H), 7.28 – 7.26 (m, 2H), 7.25 – 7.23 (m, 2H), 2.28 (s, 3H). ^{13}C NMR (126 MHz, $CDCl_3$) δ 168.47 (CO), 140.61 (C), 137.34 (C), 137.28 (C), 128.92 (2CH), 127.76 (2CH), 127.25 (CH), 126.98 (2CH), 120.31 (2CH), 25.0 (CH_3). Spectroscopic data in agreement with the literature. [S7]

***N,N*-Dimethyl-[1,1'-biphenyl]-4-carboxamide (Table 2, Entry 5)**

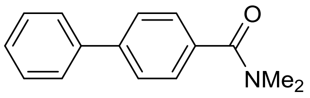

The general procedure was followed using 4-Chloro-*N,N*-dimethylbenzamide **1a**. Purification by column chromatography (hexanes/ CH_2Cl_2 1:2) gave the title compound as an off-white crystalline solid (78 mg, 69%).

^{1}H NMR (400 MHz, $CDCl_3$) δ 7.64 – 7.57 (m, 4H), 7.50 (d, J = 7.4 Hz, 2H), 7.45 (t, J = 7.6 Hz, 2H), 7.37 (tt, J = 7.6 Hz, 1.2 Hz, 1H), 3.09 (bs, 6H). ^{13}C NMR (126 MHz, $CDCl_3$) 171.58 (CO), 142.55 (C), 140.46 (C), 135.20 (C), 128.99 (2CH), 127.85 (CH), 127.79 (2CH), 127.27 (2CH), 127.17(2CH), 39.85 (CH_3), 35.47 (CH_3). Spectroscopic data in agreement with the literature. [S8]

4-Methyl-1,1'-biphenyl (Table 2, Entry 6)

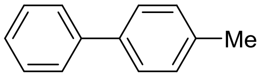

The general procedure was followed using 4-chlorotoluene and **1a** but on half the scale (0.25 mmol of aryl halide substrate). Purification by column chromatography (hexanes) gave the title compound as a white solid (38 mg, 92%).

^{1}H NMR (400 MHz, $CDCl_3$) δ 7.59 (dd, *J* = 8.3, 1.2 Hz, 2H), 7.52 – 7.48 (m, 2H), 7.43 (t, *J* = 7.5 Hz, 2H), 7.32 (tt, *J* = 7.2 Hz, 1H), 7.26 (d, *J* = 8.4 Hz, 2H), 2.4(s, 3H). ^{13}C NMR (101 MHz, $CDCl_3$) δ 141.31 (C), 138.51 (C), 137.16 (C), 129.61 2(CH), 128.85 (2CH), 127.14 (2CH)127.12 (2CH), 127.11 (CH), 21.25 (CH_3). Spectroscopic data in agreement with the literature. [S9]

4-Ethyl-1,1'-biphenyl (Table 2, Entry 7)

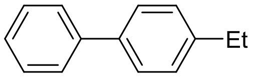

The general procedure was followed using 1-bromo-4-ethylbenzene and **1a** but on half the scale (0.25 mmol of aryl halide substrate). Purification by column chromatography (hexanes/ CH_2Cl_2 3:1) gave the title compound as a colorless oil (32 mg, 69%).

^{1}H NMR (500 MHz, $CDCl_3$) δ 7.58 (d, *J* = 7.2 Hz, 2H), 7.52 (d, *J* = 8.1 Hz, 2H), 7.43 (t, *J* = 7.7 Hz, 2H), 7.35 – 7.31 (m, 1H), 7.28 (d, *J* = 8.2 Hz, 2H), 2.70 (q, *J* = 7.6 Hz, 2H), 1.28 (t, *J* = 7.6 Hz, 3H). ^{13}C NMR (126 MHz, $CDCl_3$) δ 143.53 (C), 141.38 (C), 138.75 (C), 128.89 (CH), 128.84 (CH), 128.42 (CH), 128.37 (CH), 127.31 (CH), 127.22 (CH), 127.15 (CH), 127.10 (CH), 127.07 (CH), 28.66 (CH_2), 15.74 (CH_3). Spectroscopic data in agreement with the literature. [S6]

4-Ethyl-1,1'-biphenyl (Table 2, Entry 8)

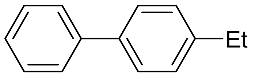

The general procedure was followed using 1-chloro-4-ethylbenzene and **1a**. Purification by column chromatography (hexanes/ CH_2Cl_2 3:1) gave the title compound as a colorless oil (72 mg, 79%).

^{1}H NMR (400 MHz, $CDCl_3$) δ 7.59 (dd, *J* = 8.3, 1.3 Hz, 2H), 7.52 (d, *J* = 8.2 Hz, 2H), 7.43 (t, *J* = 7.6 Hz, 2H), 7.32 (tt, *J* = 8.3, 1.2 Hz, 1H), 7.28 (d, *J* = 8.4 Hz, 2H), 2.70 (q, *J* = 7.6 Hz, 2H), 1.28 (t, *J* = 7.6 Hz, 3H). ^{13}C NMR (126 MHz, $CDCl_3$) δ 143.53 (C), 141.33 (C), 138.75 (C), 128.84 (2CH), 128.42 (2CH), 127.22 (2CH), 127.15 (2CH), 127.10 (CH), 28.66 (CH_2), 15.74 (CH_3). Spectroscopic data in agreement with the literature. [S6]

***N,N*-Dimethyl-[1,1'-biphenyl]-4-amine (Table 2, Entry 9)**

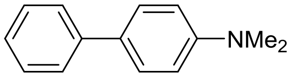

The general procedure was followed using 4-Bromo-*N*,*N*-dimethylaniline and **1a** but on half the scale (0.25 mmol of aryl halide substrate). Purification by column chromatography (hexanes/EtOAc 2:1) gave the title compound as light brown solid (39 mg, 80%).

^{1}H NMR (400 MHz, $CDCl_3$) δ 7.56 (dd, *J* = 8.3, 1.2 Hz, 2H), 7.52 (d, *J* = 8.9 Hz, 2H), 7.40 (t, *J* = 7.2 Hz, 2H), 7.26 (t, *J* = 7.2 Hz, 1H), 6.83 (d, *J* = 8.7 Hz, 2H), 3.00 (s, 6H). ^{13}C NMR (101 MHz, $CDCl_3$) δ 150.06 (C), 141.35 (C), 129.07 (C), 128.78 (2CH), 127.85 (2CH), 126.44 (2CH), 126.14 (CH), 112.98 (2CH), 40.78 ($2CH_3$). Spectroscopic data in agreement with the literature. [S10]

4-Methoxy-1,1'-biphenyl (Table 2, Entry 10)

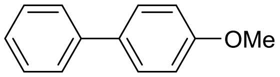

The general procedure was followed using 4-chloroanisole and **1a** but on half the scale (0.25 mmol of aryl halide substrate). Purification by column chromatography (hexanes/ CH_2Cl_2 3:1) gave the title compound as a white solid (38 mg, 82%).

^{1}H NMR (400 MHz, $CDCl_3$) δ 7.60 – 7.54 (m, 4H), 7.44 (t, *J* = 8 Hz, 2H), 7.33 (tt, *J* = 7.2, 1.2 Hz, 1H), 7.01 (d, *J* = 8.8 Hz, 2H). ^{13}C NMR (126 MHz, $CDCl_3$) δ 159.28 (C), 140.97 (C), 133.93 (C), 128.86 (2CH), 128.30 (2CH), 126.88 (2CH), 126.80 (CH), 114.34 (2CH), 55.50 (OCH_3). Spectroscopic data in agreement with the literature. [S9]

3-Methyl-1,1'-biphenyl (Table 2, Entry 11)

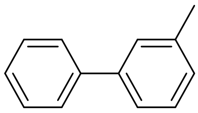

The general procedure was followed using 3-bromotoluene and **1a** but on half the scale (0.25 mmol of aryl halide substrate). Purification by column chromatography (hexanes) gave the title compound as a colorless oil (33 mg, 78%).

^{1}H NMR (400 MHz, $CDCl_3$) δ 7.59 (d, *J* = 8 Hz, 2H), 7.47 – 7.37 (m, *J* = 7.2 Hz, 4H), 7.33 (tt, *J* = 7.6, 1.2 Hz, 2H), 7.18 – 7.16 (m, 1H), 2.43 (s, 3H). ^{13}C NMR (101 MHz, $CDCl_3$) δ 141.5 (C), 141.39 (C), 138.46 (C), 128.83 (2CH), 128.80 (CH), 128.12 (2CH), 127.32 (2CH), 127.29 (CH), 124.41 (CH), 21.69 (CH_3). Spectroscopic data in agreement with the literature. [S9]

3,5-Dimethyl-1,1'-biphenyl (Table 2, Entry 12)

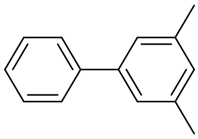

The general procedure was followed using 1-bromo-3,5-dimethylbenzene and **1a**. Purification by column chromatography (hexanes/ CH_2Cl_2 3:1) gave the title compound as a colorless oil (77 mg, 84%).

^{1}H NMR (400 MHz, $CDCl_3$) δ 7.59 (d, *J* = 8.4, 1.6 Hz, 2H), 7.46 – 7.41 (m, *J* = 7.2 Hz 2H), 7.37 – 7.33 (m, *J* = 7.2 Hz, 1H), 7.24 – 7.20 (m, 2H), 7.02 – 6.97 (m, 1H), 2.40 (s, 6H). ^{13}C NMR (101 MHz,

CDCl$_3$) δ 141.61 (C), 141.41 (C), 138.38 (2C), 129.02 (CH), 128.87 (CH), 128.76 (CH), 127.39 (CH), 127.33 (CH), 127.31 (CH), 127.21 (CH), 125.25 (CH), 21.56 (2CH$_3$). Spectroscopic data in agreement with the literature. [S11]

2-Methyl-1,1'-biphenyl (Table 2, Entry 13)

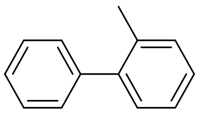

The general procedure was followed using 2-chlorotoluene and **1a.** Purification by column chromatography (hexanes/ CH$_2$Cl$_2$ 3:1) gave the title compound as a colorless oil (71 mg, 85%).
^{1}H NMR (400 MHz, CDCl$_3$) δ 7.42 (t, *J* = 7.2 Hz, 2H) 7.37 – 7.31 (m, 3H), 7.29 – 7.26 (m, *J* = 2.4 Hz, 2H), 7.24 (dt, *J* = 4.3, 2.3 Hz, 2H). ^{13}C NMR (101 MHz, CDCl$_3$) δ 142.11 (C), 142.08 (C), 135.49 (C), 130.43 (CH), 129.93 (CH), 129.33 (2CH), 128.19 (2CH), 127.38 (CH), 126.89 (CH), 125.89 (CH). Spectroscopic data in agreement with the literature. [S9]

2-Methoxy-1,1'-biphenyl (Table 2, Entry 14)

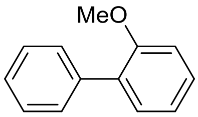

The general procedure was followed using 2-chloroanisole and **1a** but on half the scale (0.25 mmol of aryl halide substrate). Purification by column chromatography (hexanes/ CH$_2$Cl$_2$ 3:1) gave the title compound as a colorless oil (40 mg, 87%).
^{1}H NMR (400 MHz, CDCl$_3$) δ 7.54 (d, *J* = 7.0 Hz, 2H), 7.41 (t, *J* = 8.0 Hz, 2H), 7.36 – 7.30 (m, 3H), 7.04 (td, *J* = 7.4, 1.1 Hz, 1H), 7.00 (dd, *J* = 8.6, 1.1 Hz, 1H), 3.82 (CH$_3$). ^{13}C NMR (101 MHz, CDCl$_3$) δ 156.61 (C), 138.69 (C), 131.03 (CH), 130.89 (C), 129.68 (2CH), 128.74 (CH), 128.11 (2CH), 127.04 (CH), 120.97 (CH), 111.39 (CH), 55.71 (OCH$_3$). Spectroscopic data in agreement with the literature. [S9]

1-Phenylnaphthalene (Table 2, Entry 15)

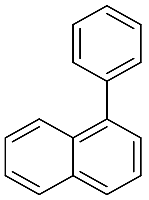

The general procedure was followed using 1-chloronaphthalene and **1a**. Purification by column chromatography (hexanes) gave the title compound as a colorless oil (74 mg, 73%).
^{1}H NMR (400 MHz, CDCl$_3$) δ 7.91 (d, *J* = 8.4 Hz, 2H), 7.87 (d, *J* = 8.2 Hz, 1H), 7.55 (d, *J* = 7.0 Hz, 1H), 7.52 – 7.48 (m, 5H), 7.47 – 7.41 (m, 3H). ^{13}C NMR (101 MHz, CDCl$_3$) δ 140.91 (C), 140.41 (C),

133.94 (C), 131.77 (C), 130.21 (2CH), 128.39 (3CH), 127.76 (CH), 127.37 (CH), 127.06 (CH), 126.17 (CH), 126.15 (CH), 125.90 (CH), 125.51 (CH). Spectroscopic data in agreement with the literature. [S5]

***N,N*-Diethyl-[1,1'-biphenyl]-2-carboxamide (Table 2, Entry 16)**

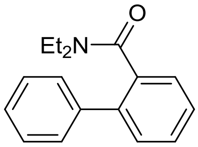

The general procedure was followed using 2-chloro-*N,N*-diethylbenzamide and **1a**. Purification by column chromatography (hexanes/EtOAc 1:1) gave the title compound as a white solid (70 mg, 55%).
^{1}H NMR (500 MHz, $CDCl_3$) δ 7.48 (d, *J* = 7, 2H), 7.45 – 7.42 (m, 1H), 7.39 (dd, *J* = 7.8, 1.2 Hz, 2H), 7.38 – 7.30 (m, 4H), 3.76 (dd, *J* = 13.7, 6.9 Hz, 1H), 2.97 (ddd, *J* = 21.2, 13.9, 7.1 Hz, 2H), 2.64 (dd, *J* = 14.3, 7.1 Hz, 1H), 0.89 (t, *J* = 7.1 Hz, 3H), 0.73 (t, *J* = 7.1 Hz, 3H). ^{13}C NMR (126 MHz, $CDCl_3$) δ 170.69 (CO), 139.95 (C), 138.52 (C), 136.50 (C), 129.55 (CH), 129.07 (CH), 129.01 (2CH), 128.43 (2CH), 127.69 (CH), 127.67 (CH), 127.12 (CH), 42.37 (CH_2), 38.45 (CH_2), 13.51 (CH_3), 12.07 (CH_3). Spectroscopic data in agreement with the literature. [S12]

4-Methoxy-4'-(trifluoromethyl)-1,1'-biphenyl (Table 2, Entry 17)

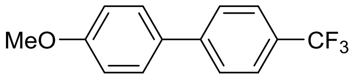

The general procedure was followed using 4-bromobenzotrifluoride and **1f**. Purification by column chromatography (hexanes/ CH_2Cl_2 3:1) gave the title compound as a white solid (103 mg, 82%).
^{1}H NMR (400 MHz, $CDCl_3$) δ 7.66 (s, 4H), 7.54 (d, *J* = 8.8 Hz, 2H), 7.00 (d, *J* = 8.8 Hz, 2H), 3.87 (s, 3H). ^{13}C NMR (126 MHz, $CDCl_3$) δ 159.98 (C), 144.40 (C), 132.33 (C), 128.83 (q, $^2J_{C\text{-}F}$ = 34.02 Hz, C), 128.50 (2CH), 127.01 (2CH), 125.81 (q, $^3J_{C\text{-}F}$ = 3.8 Hz, 2CH), 124.52 (q, $^1J_{C\text{-}F}$ = 272.16 Hz, CF_3), 114.57 (2CH), 55.54 (OCH_3). ^{19}F NMR (377 MHz, $CDCl_3$): δ = -62.35 (CF_3). Spectroscopic data in agreement with the literature. [S9]

4-Ethyl-4'-(trifluoromethyl)-1,1'-biphenyl (Table 2, Entry 18)

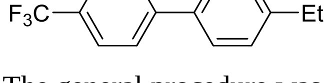

The general procedure was followed using 1-chloro-4-ethylbenzene and **1h** but on half the scale (0.25 mmol of aryl halide substrate). Purification by column chromatography (hexanes/ CH_2Cl_2 3:1) gave the title compound as a white solid (47 mg, 76%).
^{1}H NMR (400 MHz, $CDCl_3$) δ 7.68 (s, 4H), 7.53 (d, *J* = 8.2 Hz, 2H), 7.31 (d, *J* = 8.6 Hz, 2H), 2.71 (q, *J* = 7.6 Hz, 2H), 1.29 (t, *J* = 7.6 Hz, 3H). ^{13}C NMR (126 MHz, $CDCl_3$) δ 144.82 (C), 144.63(C),

137.26 (C), 129.17 (q, $^{2}J_{C-F}$ = 31.5 Hz, C), 128.66 (2CH), 127.79 (CH), 127.35 (2CH), 126.10 (q, $^{2}J_{C-F}$ = 3.8 Hz, CH), 125.80 (q, $^{3}J_{C-F}$ = 3.8 Hz, 2CH), 125.50 (q, $^{1}J_{C-F}$ = 273.42 Hz, CF_3), 28.70 (CH_2), 15.69 (CH_3). ^{19}F NMR (377 MHz, $CDCl_3$): δ = -62.38 (CF_3). Spectroscopic data in agreement with the literature. [S13]

4-(4'-Methyl-[1,1'-biphenyl]-4-yl)morpholine (Table 2, Entry 19)

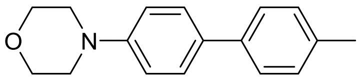

The general procedure was followed using 4-chlorotoluene and **1j** but on half the scale (0.25 mmol of aryl halide substrate). Purification by column chromatography (hexanes/ CH_2Cl_2 1:1, R_f = 0.35) gave the title compound as a white solid (46 mg, 73%). mp 173-175 °C. ^{1}H NMR (400 MHz, $CDCl_3$) δ 7.52 (d, *J* = 8.8 Hz, 2H), 7.45 (d, *J* = 8.2 Hz, 2H), 7.22 (d, *J* = 7.8 Hz, 2H), 6.99 (d, *J* = 8.2 Hz, 2H), 3.90 (t, *J* = 4.8 Hz, 4H), 3.21 (t, *J* = 4.8 Hz, 4H), 2.38 (s, 3H). ^{13}C NMR (126 MHz, $CDCl_3$) δ 150.47 (C), 138.07 (C), 136.37 (C), 132.90 (C), 129.57 (2CH), 127.76 (2CH), 126.54 (2CH), 115.98 (2CH), 67.05 ($2CH_2$), 49.44 ($2CH_2$), 21.21 (CH_3). GCMS (EI) m/z (%): 253.2 (100) $(M)^+$, 253 (20), 195 (64). HRMS: (ESI$^+$) Calculated for $[C_{17}H_{19}NO]^+$ $[M]^+$: 254.1545. Found: 254.1552. FT-IR $\tilde{v}_{max}$ (neat)/ cm^{-1}: 2964, 2855, 1605, 1507, 1227, 1120, 923, 806.

4-Fluoro-4'-methoxy-1,1'-biphenyl (Table 2, Entry 20)

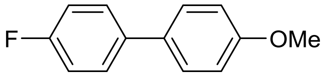

The general procedure was followed using 4-chloroanisole and **1i.** Purification by column chromatography (hexanes/ CH_2Cl_2 3:1) gave the title compound as a white solid (82 mg, 82%).

^{1}H NMR (400 MHz, $CDCl_3$) δ 7.52 – 7.45 (m, *J* = 8.8, 2.4 Hz, 4H), 7.10 (t, *J* = 8.4 Hz, 2H), 6.97 (d, *J* = 8.8 Hz, 2H), 3.85 (3H). ^{13}C NMR (126 MHz, $CDCl_3$) δ 162.09 (d, $^{1}J_{C-F}$ = 245.5 Hz, C), 159.11 (C), 136.98 (C), 132.84 (C), 128.21 (d, $^{3}J_{C-F}$ = 7.9 Hz, (2CH), 128.03 (2CH), 115.53 (d, $^{2}J_{C-F}$ = 21.4 Hz, 2CH), 114.25 (2CH), 55.37 (OCH_3). ^{19}F NMR (377 MHz, $CDCl_3$): δ = -116.78 (F). Spectroscopic data in agreement with the literature. [S9]

2,4'-Dimethoxy-1,1'-biphenyl (Table 2, Entry 21)

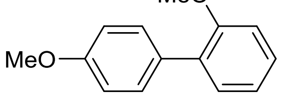

The general procedure was followed using 2-chloroanisole and **1f**. Purification by column chromatography (hexanes/ CH_2Cl_2 2:1) gave the title compound as a colorless oil (83 mg, 78%).

^{1}H NMR (400 MHz, $CDCl_3$) δ 7.48 (d, *J* = 8.8 Hz, 4H), 6.96 (d, *J* = 8.8 Hz, 4H), 3.85 (s, 6H). ^{13}C NMR (126 MHz, $CDCl_3$) δ 158.84 (2C), 133.63 (2C), 127.88 (4CH), 114.31 (4CH), 55.50 (2CH_3). Spectroscopic data in agreement with the literature. [S9]

4-(Benzyloxy)-1,1'-biphenyl (Table 2, Entry 22)

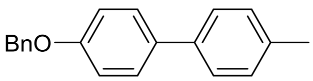

The general procedure was followed using 2-chlorotoluene and **1g** but on half the scale (0.25 mmol of aryl halide substrate). Purification by column chromatography (hexanes/ CH_2Cl_2 2:1) gave the title compound as a white solid (56 mg, 82%).

^{1}H NMR (500 MHz, $CDCl_3$) δ 7.51 (d, *J* = 8.8 Hz, 2H), 7.48 – 7.43 (m, 4H), 7.40 (t, *J* = 7.2 Hz, 2H), 7.36 – 7.33 (m, 1H), 7.22 (d, *J* = 7.8 Hz, 2H), 7.04 (d, *J* = 8.8 Hz, 2H), 5.11 (s, 2H), 2.38 (s, 3H). ^{13}C NMR (126 MHz, $CDCl_3$) δ 158.29 (C), 138.06 (C), 137.17 (C), 136.53 (C), 134.15 (C), 129.62 (CH), 129.58 (CH), 128.76 (CH), 128.72 (CH), 128.11 (2CH), 127.63 (2CH), 126.73 (2CH), 115.24 (2CH), 114.99 (CH), 70.25 (CH_2), 21.20 (CH_3). Spectroscopic data in agreement with the literature. [S14]

3,5-Dimethoxy-4'-(trifluoromethyl)-1,1'-biphenyl (Table 2, Entry 23)

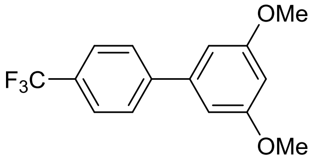

The general procedure was followed using 5-chloro-1,3-dimethoxybenzene and **1h** but on half the scale (0.25 mmol of aryl halide substrate). Purification by column chromatography (hexanes/ CH_2Cl_2 2:1) gave the title compound as a colorless oil (60 mg, 85%).

^{1}H NMR (400 MHz, $CDCl_3$) δ 7.68 (s, 4H), 6.72 (d, *J* = 2.3 Hz, 2H), 6.52 (t, *J* = 2.3 Hz, 1H), 3.86 (s, 6H). ^{13}C NMR (126 MHz, $CDCl_3$) δ 161.35 (2C), 144.84 (C), 141.09 (C), 129.73 (q, $^2J_{C\text{-}F}$ = 32.5 Hz, C), 127.63 (2CH), 125.79 (q, $^3J_{C\text{-}F}$ = 3.7 Hz, 2CH), 124.39 (q, $^1J_{C\text{-}F}$ = 272.16 Hz, CF_3) 105.78 (2 CH), 100.08 (CH), 55.62 (2 OCH_3).^{19}F NMR (377 MHz, $CDCl_3$): δ = -62.45 (CF_3). GCMS (EI) m/z (%): 282.2 (100) $(M)^+$, 253 (20), 183 (20), 139 (24). FT-IR $\tilde{\nu}_{max}$ (neat)/ cm^{-1}: 2933, 1594, 1321, 1155, 1110, 1066, 1016, 831. Spectroscopic data in agreement with the literature. [S6]

4-(Trifluoromethoxy)-1,1'-biphenyl (Table 2, Entry 24)

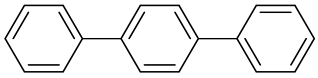

The general procedure was followed using 4-bromobiphenyl and **1a** but on half the scale (0.25 mmol of aryl halide substrate). Purification by column chromatography (hexanes/ CH_2Cl_2 3:1) gave the title compound as a white solid (34 mg, 58%).

^{1}H NMR (400 MHz, $CDCl_3$) δ 7.69 (s, 4H), 7.65 (d, *J* = 7.1 Hz, 4H), 7.47 (t, *J* = 7.5 Hz, 4H), 7.37 (t, *J* = 7.4 Hz, 2H). ^{13}C NMR (126 MHz, $CDCl_3$) δ 140.86 (2C), 140.28 (2C), 128.96 (4CH), 127.64 (4CH), 127.48 (2CH), 127.20 (4CH). Spectroscopic data in agreement with the literature. [S15]

3-Phenylquinoline (Table 2, Entry 25)

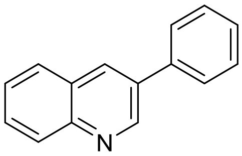

The general procedure was followed using 3-bromoquinoline and **1a**. Purification by column chromatography (hexanes/EtOAc 2:1) gave the title compound as a white solid (38 mg, 37%).
^{1}H NMR (400 MHz, $CDCl_3$) δ 9.19 (s, 0H), 8.32 (s, 0H), 8.15 (d, *J* = 9.5 Hz, 1H), 7.89 (d, *J* = 8.1 Hz, 1H), 7.77 – 7.69 (m, 3H), 7.59 (t, *J* = 7.5 Hz, 1H), 7.54 (t, *J* = 7.7 Hz, 2H), 7.45 (t, *J* = 7.4 Hz, 1H). ^{13}C NMR (126 MHz, $CDCl_3$) δ 150.04 (CH), 147.42 (C), 138.02 (C), 134.03 (C), 133.47 (CH), 129.59 (3CH), 129.34 (CH), 128.28 (CH), 128.19 (C), 128.16 (CH), 127.59 (2CH), 127.19 (CH). Spectroscopic data in agreement with the literature. [S16]

8-Phenylquinoline (Table 2, Entry 26)

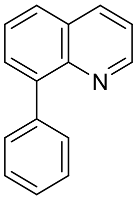

The general procedure was followed using 8-bromoquinoline and **1a**. Purification by column chromatography (hexanes/EtOAc 2:1) gave the title compound as a white solid (36 mg, 35%).
^{1}H NMR (400 MHz, $CDCl_3$) δδ 8.96 (dd, *J* = 4.2, 1.8 Hz, 1H), 8.21 (dd, *J* = 8.2, 1.8 Hz, 1H), 7.83 (dd, *J* = 8.1, 1.5 Hz, 1H), 7.72 (m, *J* = 7.6, 1.3 Hz, 3H), 7.61 (dd, *J* = 8.1, 7.2 Hz, 1H), 7.50 (t, *J* = 7.7 Hz, 2H), 7.42 (dd, *J* = 8.3, 4.0 Hz, 2H). ^{13}C NMR (126 MHz, $CDCl_3$) δ 150.40 (CH), 146.15 (C), 141.06 (C), 139.67 (C), 136.43 (CH), 130.74 (2CH), 130.48 (CH), 128.88 (C), 128.15 (2CH), 127.67 (CH), 127.52 (CH), 126.42 (CH), 121.13 (CH). Spectroscopic data in agreement with the literature. [S17]

3-Phenylbenzo[b]thiophene (Table 2, Entry 27)

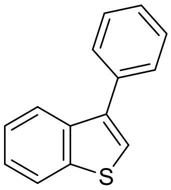

The general procedure was followed using 2-bromothionaphthene and **1a**. Purification by column chromatography (hexanes/EtOAc 2:1) gave the title compound as a colorless oil (34 mg, 33%).
^{1}H NMR (400 MHz, $CDCl_3$) δ 7.93 (d, J = 9.3 Hz, 2H), 7.60 (d, J = 8.4 Hz, 2H), 7.50 (t, J = 7.6 Hz, 2H), 7.42 – 7.33 (m, 4H). ^{13}C NMR (126 MHz, $CDCl_3$) δ 140.70 (C), 138.12 (C), 137.92 (C), 136.03 (C), 128.75 (2CH), 128.74 (2CH), 127.56 (CH), 124.42 (CH), 124.34 (CH), 123.41 (CH), 122.94 (2CH). Spectroscopic data in agreement with the literature. [S18]

2-([1,1'-Biphenyl]-2-yl)-5-methyl-2,4-dihydro-3H-pyrazol-3-one (5a)

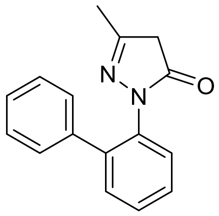

The general procedure was followed using 1-(2-Chlorophenyl)-3-methyl-2-pyrazolin-5-one and **1a** but on half the scale (0.25 mmol of aryl halide substrate). Purification by column chromatography (hexanes/EtOAc 1:1, R_f = 0.35) gave the title compound as a light brown solid (52 mg, 85%). ^{1}H NMR (500 MHz, $CDCl_3$) δ 7.45 – 7.40 (m, J = 7.6, 2 Hz, 4H), 7.36 – 7.31 (m, 4H), 7.32 – 7.27 (m, 1H), 3.12 (s, 2H), 2.04 (s, 3H). ^{13}C NMR (126 MHz, $CDCl_3$) δ 171.53 (CO), 155.91 (C), 140.17 (C), 139.50 (C), 134.58 (C), 131.02 (CH), 129.11 (CH), 128.56 (2CH), 128.46 (CH), 128.34 (2CH), 127.91 (CH), 127.36 (CH), 41.47 (CH_2), 17.07 (CH_3). Spectroscopic data in agreement with the literature. [S19]

5-Methyl-2-(4'-methyl-[1,1'-biphenyl]-2-yl)-2,4-dihydro-3H-pyrazol-3-one (5b)

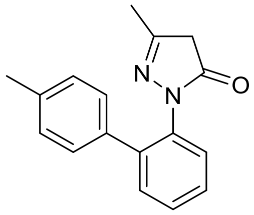

The general procedure was followed using 1-(2-Chlorophenyl)-3-methyl-2-pyrazolin-5-one and **1e** but on half the scale (0.25 mmol of aryl halide substrate). Purification by column chromatography (hexanes/EtOAc 1:1) gave the title compound as a brown solid (48 mg, 73%, R_f = 0.3). ^{1}H NMR (500 MHz, $CDCl_3$) δ 7.44 – 7.39 (m, 4H), 7.23 (d, J = 8.1 Hz, 2H), 7.15 (d, J = 7.7 Hz, 2H), 3.13 (s, 2H), 2.36 (s, 3H), 2.06 (s, 3H). ^{13}C NMR (126 MHz, $CDCl_3$) δ 171.48 (CO), 155.92 (C), 140.19 (C), 137.02 (C), 136.56 (C), 134.55 (C), 131.04 (CH), 129.11 (3CH), 128.37 (2CH), 128.24 (CH), 127.94 (CH), 41.51 (CH_2), 21.34 (CH_3), 17.12 (CH_3). Spectroscopic data in agreement with the literature. [S19]

2-(4'-Methoxy-[1,1'-biphenyl]-2-yl)-5-methyl-2,4-dihydro-3H-pyrazol-3-one (5c)

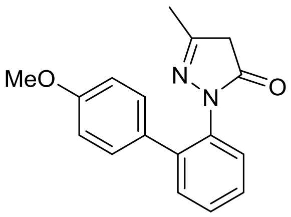

The general procedure was followed using 1-(2-Chlorophenyl)-3-methyl-2-pyrazolin-5-one and **1f**. (prepared in situ, 3 equiv). Purification by column chromatography (hexanes/EtOAc 1:1, R_f = 0.3) gave the title compound as an off-white solid (50 mg, 71%).

^{1}H NMR (500 MHz, $CDCl_3$) δ 7.44 – 7.37 (m, 4H), 7.26 (d, *J* = 8.2 Hz, 2H), 6.89 (d, *J* = 8.7 Hz, 2H), 3.82 (s, 3H), 3.14 (s, 2H), 2.06 (s, 3H). ^{13}C NMR (126 MHz, $CDCl_3$) δ 171.52 (CO), 158.99 (C), 155.95 (C), 139.96 (C), 134.58 (C), 131.89 (C), 131.01 (CH), 129.68 (2CH), 129.15 (CH), 128.11 (CH), 128.02 (CH), 113.81 (2CH), 55.35 (OCH_3), 41.51 (CH_2), 17.14 (CH_3). Spectroscopic data in agreement with the literature. [S19]

2-(4'-(Benzyloxy)-[1,1'-biphenyl]-2-yl)-5-methyl-2,4-dihydro-3H-pyrazol-3-one (5d)

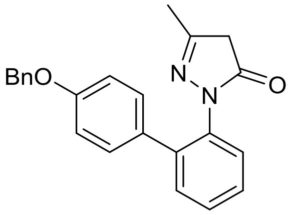

The general procedure was followed using 1-(2-Chlorophenyl)-3-methyl-2-pyrazolin-5-one and **1g** but on half the scale (0.25 mmol of aryl halide substrate). Purification by column chromatography (hexanes/EtOAc 1:1, R_f = 0.25) gave the title compound as a brown solid (52 mg, 59%); mp 86-88 °C.

^{1}H NMR (500 MHz, $CDCl_3$) δ 7.45 (d, *J* = 6.8 Hz, 2H), 7.44 – 7.35 (m, 6H), 7.34 (t, *J* = 7.3 Hz, 1H), 7.27 (m, 2H), 6.96 (d, *J* = 8.7 Hz, 2H), 5.08 (s, 2H), 3.13 (s, 2H), 2.05 (s, 3H). ^{13}C NMR (126 MHz, $CDCl_3$) δ 171.56 (CO), 158.22 (C), 155.96 (C), 139.93 (C), 137.08 (C), 134.61 (C), 132.16 (C), 130.99 (CH), 129.71 (2CH), 129.16 (CH), 128.74 (2CH), 128.15 (2CH), 128.02 (CH), 127.70 (2CH), 114.75 (2CH), 70.12 (CH_2), 41.51(CH_2), 17.14 (CH_3). HRMS: (ESI^+) Calculated for $[C_{23}H_{20}N_2O_2]^+$ $[M]^+$: 357.1603. Found: 357.1605. FT-IR $\tilde{\nu}_{max}$ (neat)/ cm^{-1}: 3032, 2932, 1721, 1606, 1515, 1484, 1386, 1237, 1176, 1013, 830, 728, 695.

5-Methyl-2-(4'-(trifluoromethyl)-[1,1'-biphenyl]-2-yl)-2,4-dihydro-3H-pyrazol-3-one (5e)

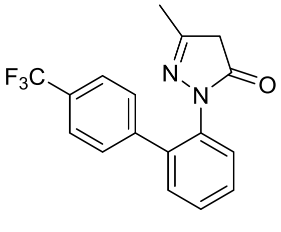

The general procedure was followed using 1-(2-Chlorophenyl)-3-methyl-2-pyrazolin-5-one and **1h** but on half the scale (0.25 mmol of aryl halide substrate). Purification by column chromatography (hexanes/EtOAc 1:1, R_f = 0.35) gave the title compound as an off-white solid (49 mg, 62%); mp 142-144 °C. ^{1}H NMR (500 MHz, $CDCl_3$) δ 7.61 (d, *J* = 8.1 Hz, 2H), 7.50 – 7.39 (m, 6H), 3.15 (s, 2H), 2.06 (s, 3H). ^{13}C NMR (126 MHz, $CDCl_3$) δ 171.36 (CO), 156.38 (C), 143.29 (C), 138.54(C), 134.55 (C), 130.83 (CH), 129.50 (q, $^2J_{C-F}$ = 32.76 Hz, C), 129.23 (CH), 129.16 (CH), 128.96 (2CH), 127.81 (CH), 125.31 (q, $^3J_{C-F}$ = 3.8 Hz, 2CH), 124.36 (q, $^1J_{C-F}$ = 272.0 Hz, CF_3), 41.51 (CH_2), 17.11 (CH_3). ^{19}F NMR (377 MHz, $CDCl_3$): δ = -62.46 (CF_3). GCMS (EI) m/z (%): 318 (57) $(M)^+$, 235 (42), 201 (100), 152 (42). HRMS: (ESI^+) Calculated for $[C_{17}H_{13}F_3N_2O]^+$ $[M]^+$: 319.105. Found: 319.1065. FT-IR $\tilde{v}_{max}$ (neat)/ cm^{-1}: 1618, 1539, 1374, 1322, 1166, 1120, 1069, 843, 762, 684.

2-(4'-Fluoro-[1,1'-biphenyl]-2-yl)-5-methyl-2,4-dihydro-3H-pyrazol-3-one (5f)

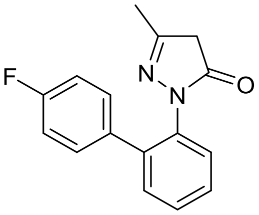

The general procedure was followed using 1-(2-Chlorophenyl)-3-methyl-2-pyrazolin-5-one and **1i** but on half the scale (0.25 mmol of aryl halide substrate). Purification by column chromatography (hexanes/EtOAc 1:1, R_f = 0.35) gave the title compound as an off-white solid (57 mg, 85%).
^{1}H NMR (500 MHz, $CDCl_3$) δ 7.45 – 7.38 (m, 4H), 7.30 (dd, *J* = 8.7, 5.4 Hz, 2H), 7.04 (t, *J* = 8.8 Hz, 2H), 3.14 (s, 2H), 2.06 (s, 3H). ^{13}C NMR (126 MHz, $CDCl_3$) δ 171.45 (CO), 162.33 (d, $^1J_{C-F}$ = 246.4 Hz, C), 156.12 (C), 139.18 (C), 135.49 (C), 134.62 (C), 130.94 (CH), 130.24 (d, $^3J_{C-F}$ = 8.1 Hz, 2CH), 129.14 (CH), 128.63 (CH), 127.93 (CH), 115.29 (d, $^2J_{C-F}$ = 21.5 Hz, 2CH), 41.48 (CH_2), 17.11 (CH_3). ^{19}F NMR (377 MHz, $CDCl_3$): δ = -115.29 (F). Spectroscopic data of compound was accordant with the literature report. [S19]

5-Methyl-2-(4'-morpholino-[1,1'-biphenyl]-2-yl)-2,4-dihydro-3H-pyrazol-3-one (5g)

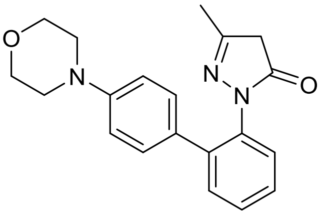

The general procedure was followed using 1-(2-Chlorophenyl)-3-methyl-2-pyrazolin-5-one and **1j** but on half the scale (0.25 mmol of aryl halide substrate). Purification by column chromatography (hexanes/EtOAc 1:1, R_f = 0.25) gave the title compound as a brown solid (39 mg, 47%); mp 125-127 °C. ^{1}H NMR (500 MHz, $CDCl_3$) δ 7.43 – 7.37 (m, 4H), 7.28 – 7.26 (m, 4H), 3.89 (s, 4H), 3.20 (q, *J* =

4.9 Hz, 4H), 3.16 (s, 2H), 2.08 (s, 3H), 7.43 – 7.37 (m, 1H), 7.26 (s, 2H), 3.89 (s, 2H), 3.20 (s, 2H), 3.16 (s, 0H), 2.08 (s, 1H). ^{13}C NMR (126 MHz, $CDCl_3$) δ 171.56 (CO), 155.94 (C), 140.10 (C), 134.52 (3C), 130.99 (CH), 129.43 (CH), 129.20 (CH), 128.10 (CH), 128.02 (CH), 115.44 (CH), 66.98 (4CH2), 41.55 (CH2), 17.19 (CH3). GCMS (EI) m/z (%): 324 (100) $(M)^+$, 266 (34), 208 (60). HRMS: (ESI^+) Calculated for $[C_{20}H_{21}N_3O_2]^+$ $[M]^+$: 336.1712. Found: 336.1717. FT-IR $\tilde{v}_{max}$ (neat)/ cm^{-1}: 2855, 1608, 1556, 1522, 1486, 1448, 1229, 1119, 925, 826, 761, 628.

2.6 Unsuccessful Reactions

Table S4 outlines the reactions that gave little or no product (as determined by GCMS analysis), while Scheme S1 outlines the observed competitive nucleophilic attack processes observed using a diarylketonic substrate.

Table S4. Reactions with trace or no conversion

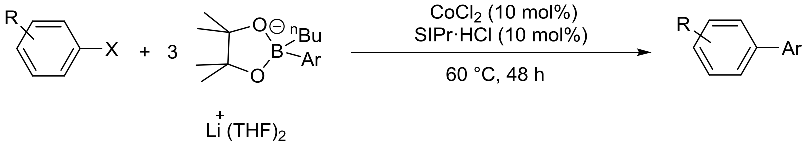

1a

**Entry**	**R**	**X**
1	4-CN	Cl, Br
2	4-NO_2	Br
3	2-CHO	Cl
4	2-$COCH_3$	Cl
5	4-NH_2	Cl
6	4-CHO	Cl
7	4-$COCH_3$	Cl
8	4-Me	4- vinylphenyl[b]
9	2-Bromoquinoline	Br
10	5-Bromoindole	Br
11	2-Bromothiophene	Br
12	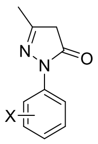	3-Cl
13	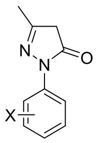	4-Cl

[a] Reaction conditions: Aryl halide (0.25 mmol), **1a** (0.75 mmol), $CoCl_2$ (0.025 mmol, 10 mol%), SIPr·HCl (0.025 mmol, 10 mol%), 60 °C, 48 h. [b] Spectroscopic yield 12%, determined by ^{1}H NMR spectroscopy (1,3,5-trimethoxybenzene internal standard).

Scheme S1. Cross-coupling with competitive nucleophilic addition

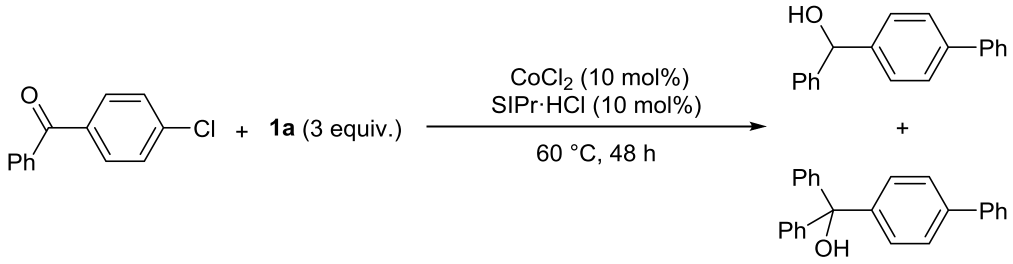

Reaction conditions: 4-chlorobenzophenone (0.25 mmol), **1a** (0.75 mmol), $CoCl_2$ (0.025 mmol, 10 mol%), SIPr·HCl (0.025 mmol, 10 mol%), 60 °C, 48 h. Products observed by GCMS.

2.7 Preparation and Reactivity of Cobalt(0) Complexes

Preparation of [Co(SIPr)(dvtms)], 6a.

(a) Using KC_8 as a reducing agent

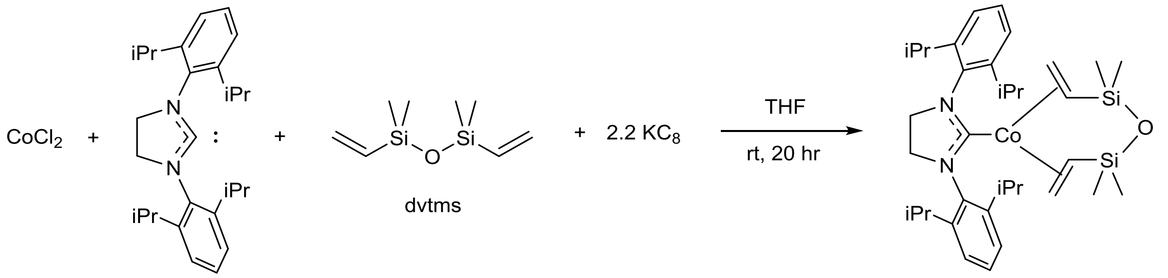

6a

$CoCl_2$ (0.065 g, 0.50 mmol) and SIPr (0.195 g, 0.50 mmol) were stirred together in THF (4 ml) for 1 hour at room temperature. To this blue solution was added dvtms (0.115 ml, 0.50 mmol) and the solution was stirred for 5 minutes. A slurry of KC_8 (0.148 g, 1.10 mmol) in THF (2 ml) was transferred via cannula to the blue reaction solution, upon which an immediate colour change to dark green was observed. After allowing to stir at room temperature for 20 hours, the reaction mixture was filtered through Celite to afford a green solution, concentrated under vacuum, washed with hexane (1 ml) and dried under vacuum to afford **6a** as a green powder (0.232 g, 66 %). Single crystals of **6a** suitable for x-ray crystallography were obtained by slow evaporation of a diethyl ether solution at room temperature. M.p. = 166-168 °C. Magnetic susceptibility (C_6D_6, 298 K): μ_{eff} = 2.9(6) μB. UV-Vis absorption spectrum (THF): λ_{max}, nm (ε, M^{-1} cm^{-1}) = 248 (19900), 320 (5590), 383 (2854), 660 (83), 768 (66). Figure S1 shows the ^1H NMR (400 MHz, C_6D_6, rt) of **6a.**

Figure S1. ^{1}H NMR of [CoSIPr(dvtms)], **6a**.

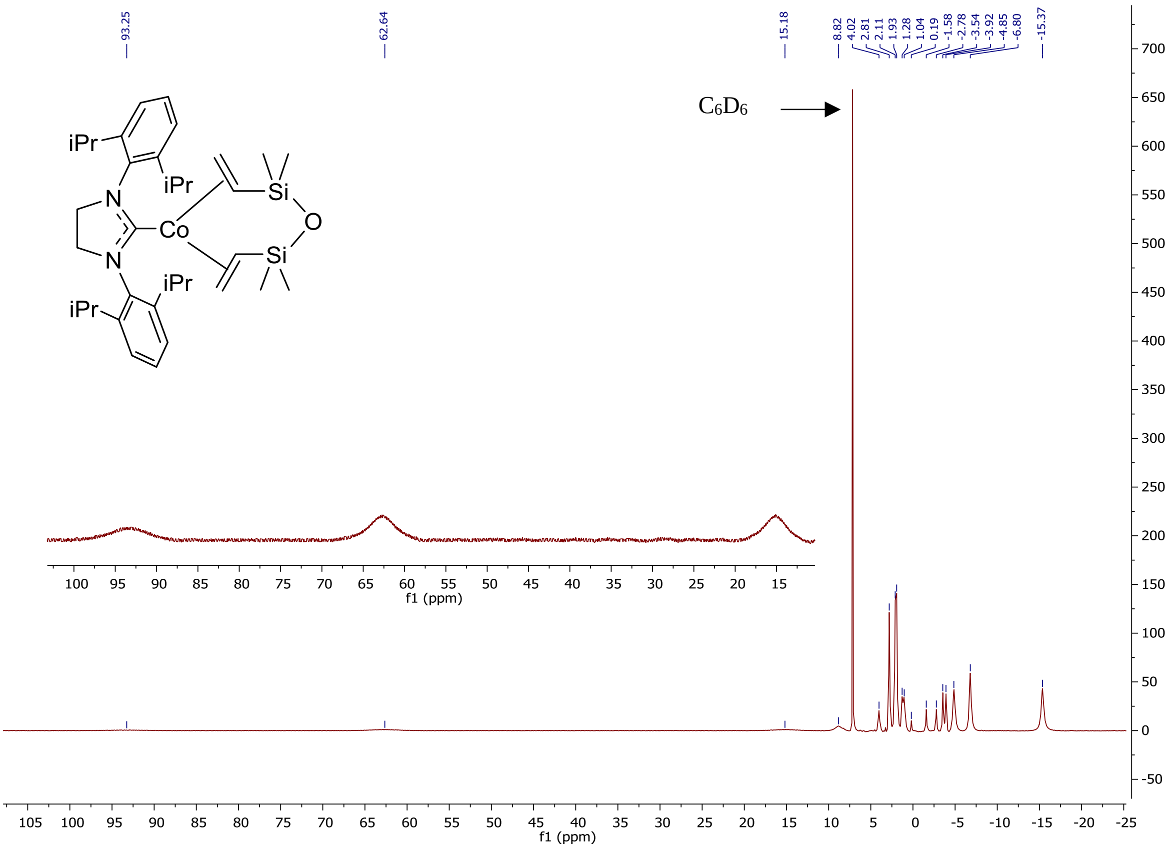

(b) Using ***1a*** *as a reducing agent*

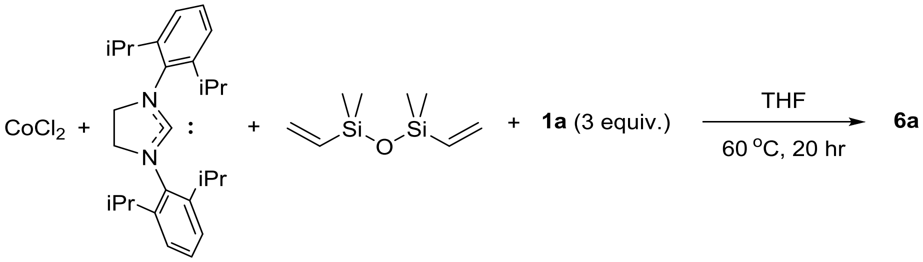

A mixture of $CoCl_2$ (0.013 g, 0.1 mmol) and SIPr (0.039 g, 0.1 mmol) were stirred in THF (1 ml) for 1 hour. To the resulting blue solution, dvtms (0.023 mL, 0.1 mmol) was added. A THF solution of **1a** (0.75 mmol), prepared as described above, was transferred to the reaction mixture. The reaction was stirred at room temperature for 1 hour and a green solution formed. The solution was filtered, the solvent removed under reduced pressure and a sample of the crude product was analysed by ^{1}H NMR spectroscopy, see Figure S2.

Figure S2. ^{1}H NMR spectra of **6a** by procedures (a) and (b).

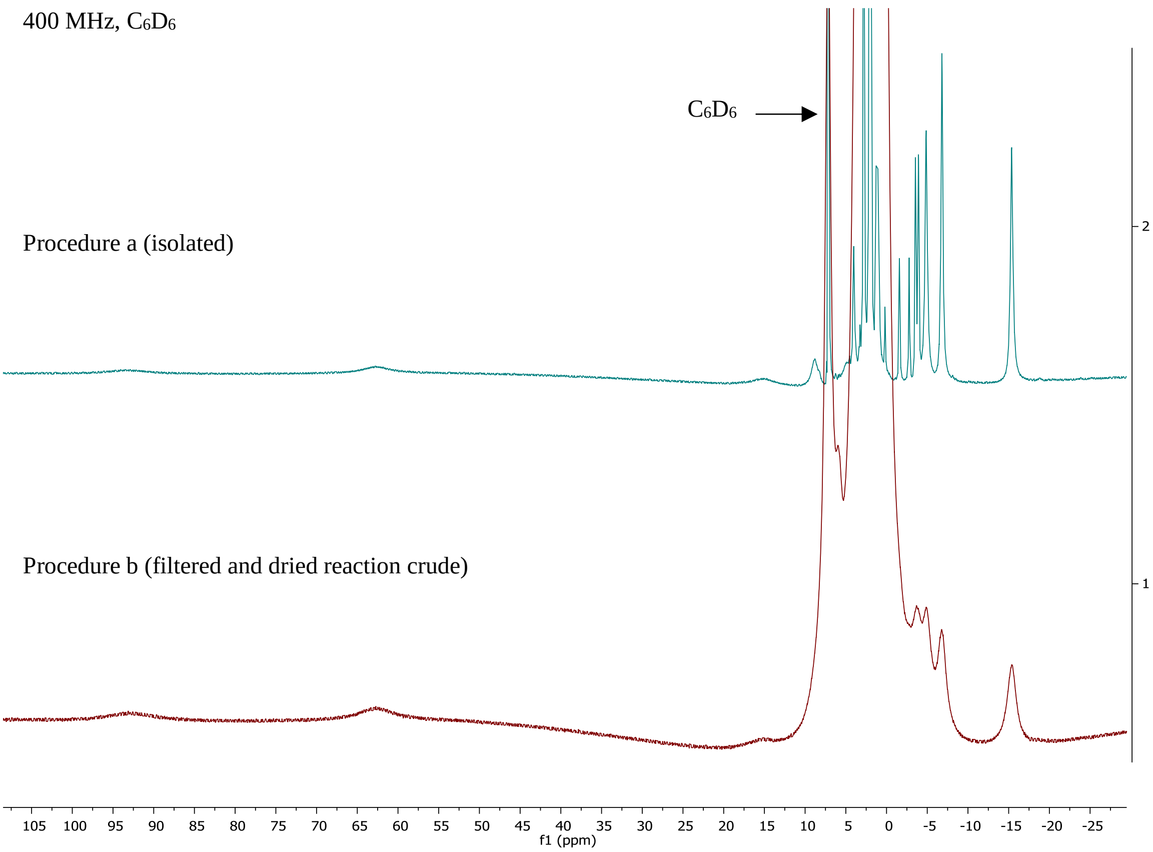

Preparation of [Co(SIPr)(norbornene)$_2$], 6b.

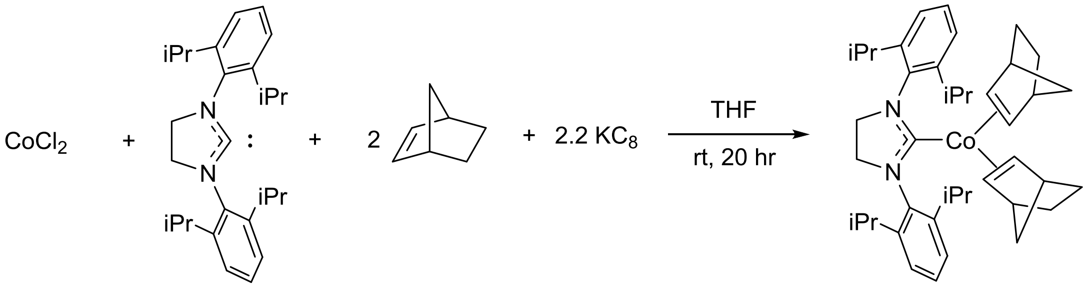

6b

$CoCl_2$ (0.083 g, 0.64 mmol) and SIPr (0.250 g, 0.64 mmol) were stirred in THF (4 ml) for 1 hour at room temperature to form a blue solution. Norbornene (0.121 g, 1.28 mmol) was added and the solution was stirred for a further 5 minutes, after which, a slurry of KC_8 (0.174 g, 1.30 mmol) in THF (2 ml) was transferred to via cannula. A green mixture was observed and the reaction was stirred at room temperature for 20 hours. The reaction mixture was then filtered through Celite, concentrated under vacuum to give a yellow-green solid that was washed with hexane (1 ml) and dried under vacuum to afford **6b** as a yellow-green powder (0.309 g, 76 %). Slow evaporation of a diethyl ether solution of **6b** at room temperature yielded single crystals suitable for x-ray crystallography. M.p. =

125-126 °C (decomp.). Magnetic susceptibility (C_6D_6, 298 K): μ_{eff} = 2.6(5) μB. UV-Vis absorption spectrum (THF): λ_{max}, nm (ε, M^{-1} cm^{-1}) = 255 (10700), 285 (5600), 379 (2240), 620 (82). ^{1}H NMR (400 MHz, C_6D_6, rt): δ 94.47 (br, 2H), 34.01 (br, 2H), 17.97 (br, 2H), 9.64 (br, 2H), 4.14-1.21 (br, 36H), -4.36 (br, 4H), -8.57 (br, 2H), -15.00 (br, 4H), -30.56 (br, 4H). The ^{1}H NMR spectrum is shown in Figure S3.

Figure S3. ^{1}H NMR spectrum of [CoSIPr(nbe)$_2$], **6b.**

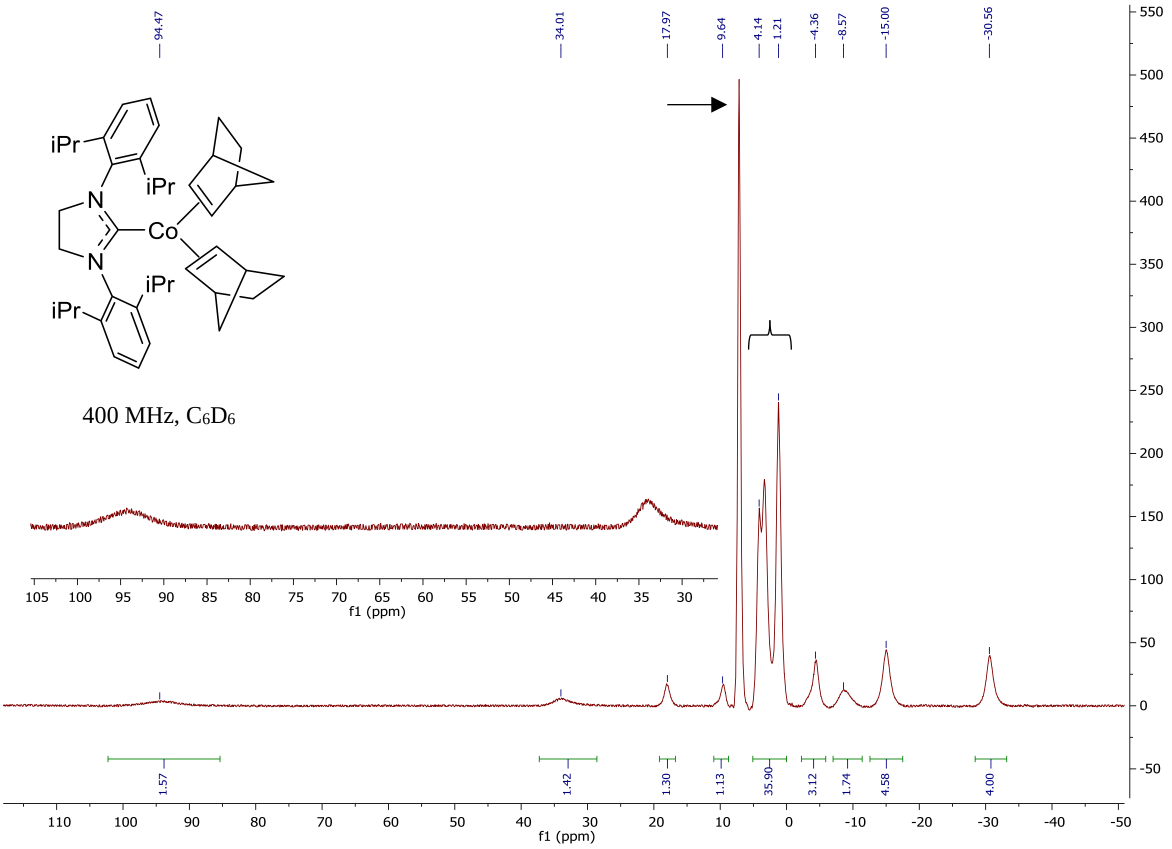

Cross-coupling using 6a as a pre-catalyst

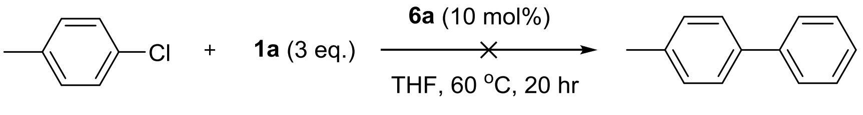

2a

To a THF (2 m) solution of **6a** (0.0159 g, 0.025 mmol), 4-chlorotoluene (0.0296 ml, 0.25 mmol) was added, followed by the transfer of **1a** (0.75 mmol). The reaction was stirred at 60 °C for 20 hours and then quenched with HCl (1 M in H_2O, 2 ml). 1,3,5-trimethoxybenzene (0.042 g, 0.25 mmol) was added as an internal standard. The work-up stated in the general procedure for cross-coupling reactions was followed. No cross-coupled product **2a** was observed.

Cross-coupling using dvtms as an additive.

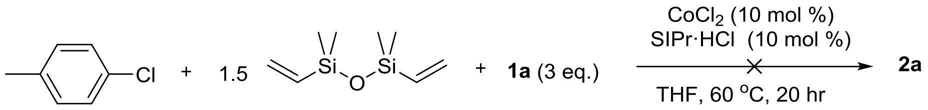

The procedure described for the cross-coupling of 4-chlorotoluene (0.0296 ml, 0.25 mmol) and **1a** (0.75 mmol), using $CoCl_2$ (0.0033 g, 0.025 mmol) as the pre-catalyst and SIPr·HCl (0.0107 g, 0.025 mmol) as the ligand was followed. However, before the addition of **1a**, dvtms (0.0864 mL, 0.375 mmol) was added. The work-up was carried out following the general procedure. No cross-coupled product **2a** was observed.

Cross-coupling using 6b as a pre-catalyst.

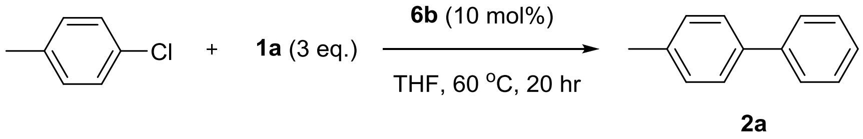

The procedure described for the cross-coupling using **6a** as a catalyst was followed, however, **6a** was replaced with **6b** (0.0159 g, 0.025 mmol). ^{1}H NMR spectroscopy showed a 60% spectroscopic yield of **2a**.

2.8 ^{1}H and ^{13}C NMR Spectra of Cross-Coupled Products.

^{1}H NMR of Ethyl [1,1'-biphenyl]-4-carboxylate (Table 2, Entry 1)

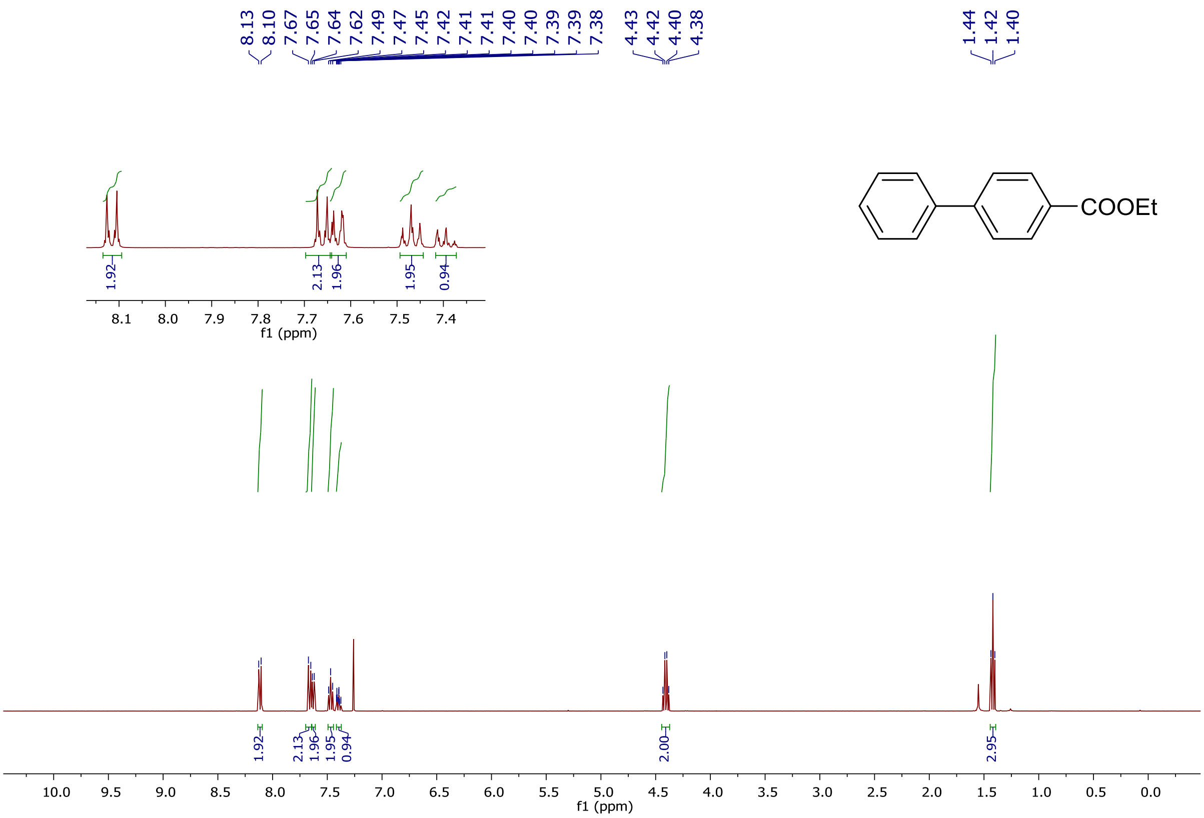

^{13}C NMR of Ethyl [1,1'-biphenyl]-4-carboxylate (Table 2, Entry 1

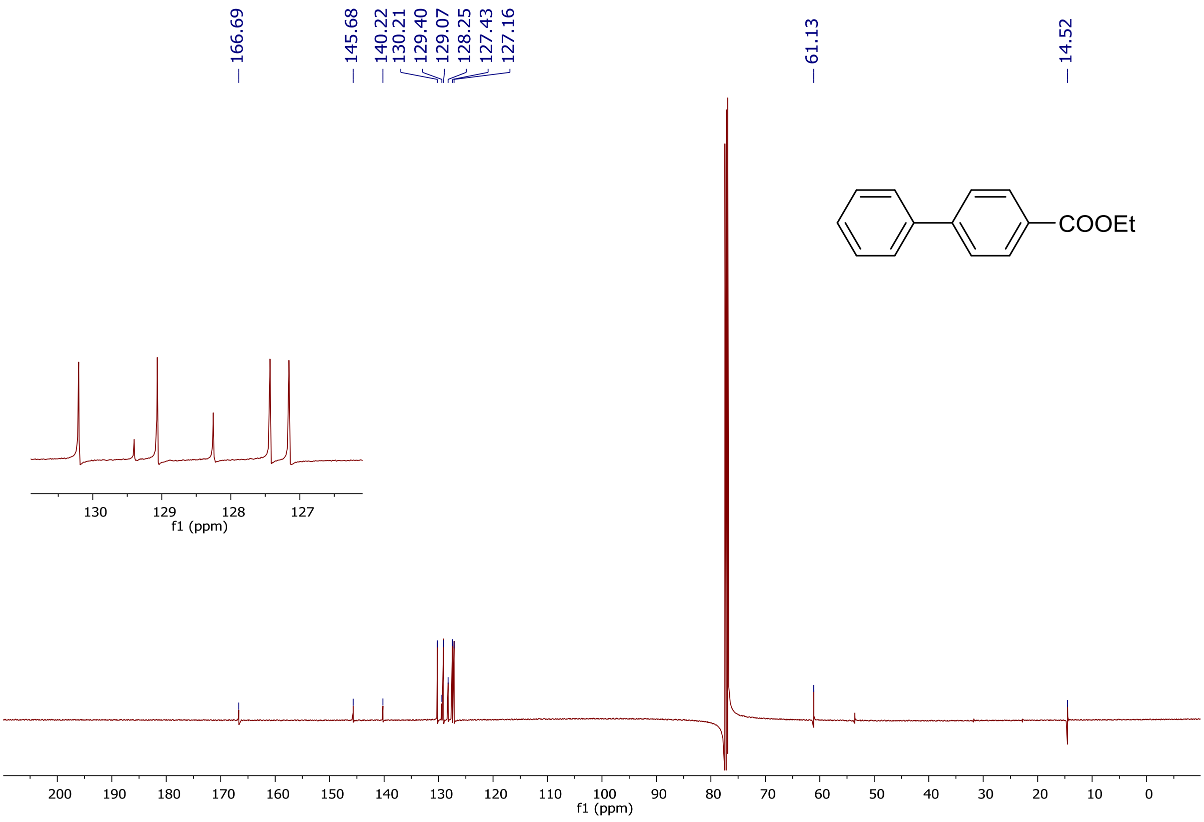

^{1}H NMR of 4-(Trifluoromethyl)-1,1'-biphenyl (Table 2, Entry 2)

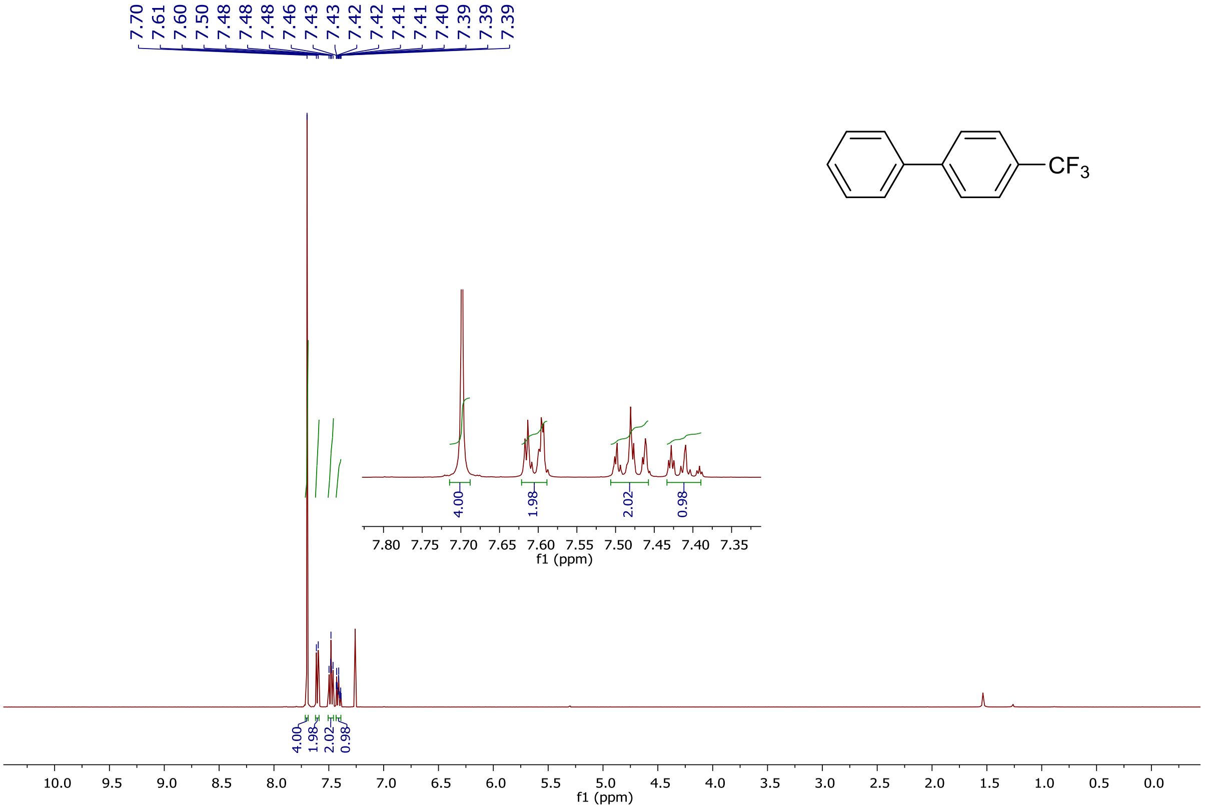

^{13}C NMR of 4-(Trifluoromethyl)-1,1'-biphenyl (Table 2, Entry 2)

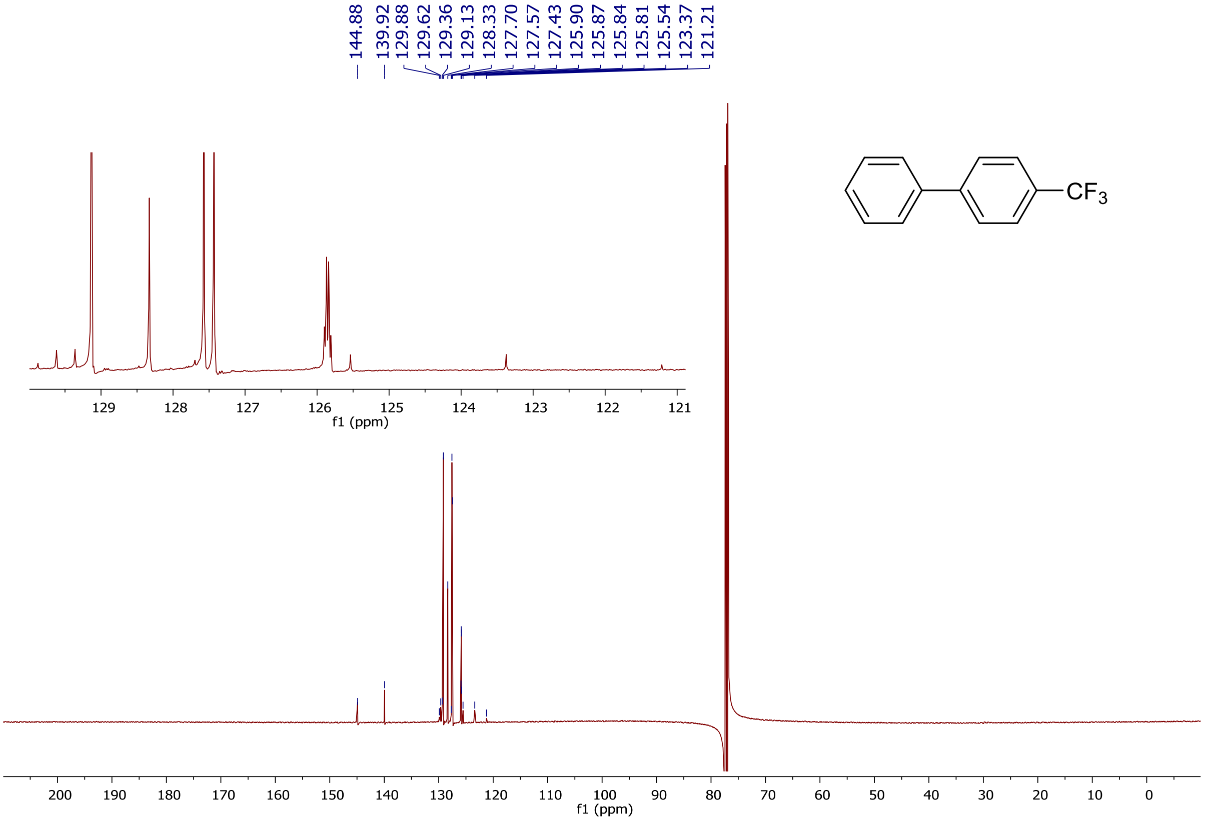

^{1}H NMR of 4-Fluoro-1,1'-biphenyl (Table 2, Entry 3)

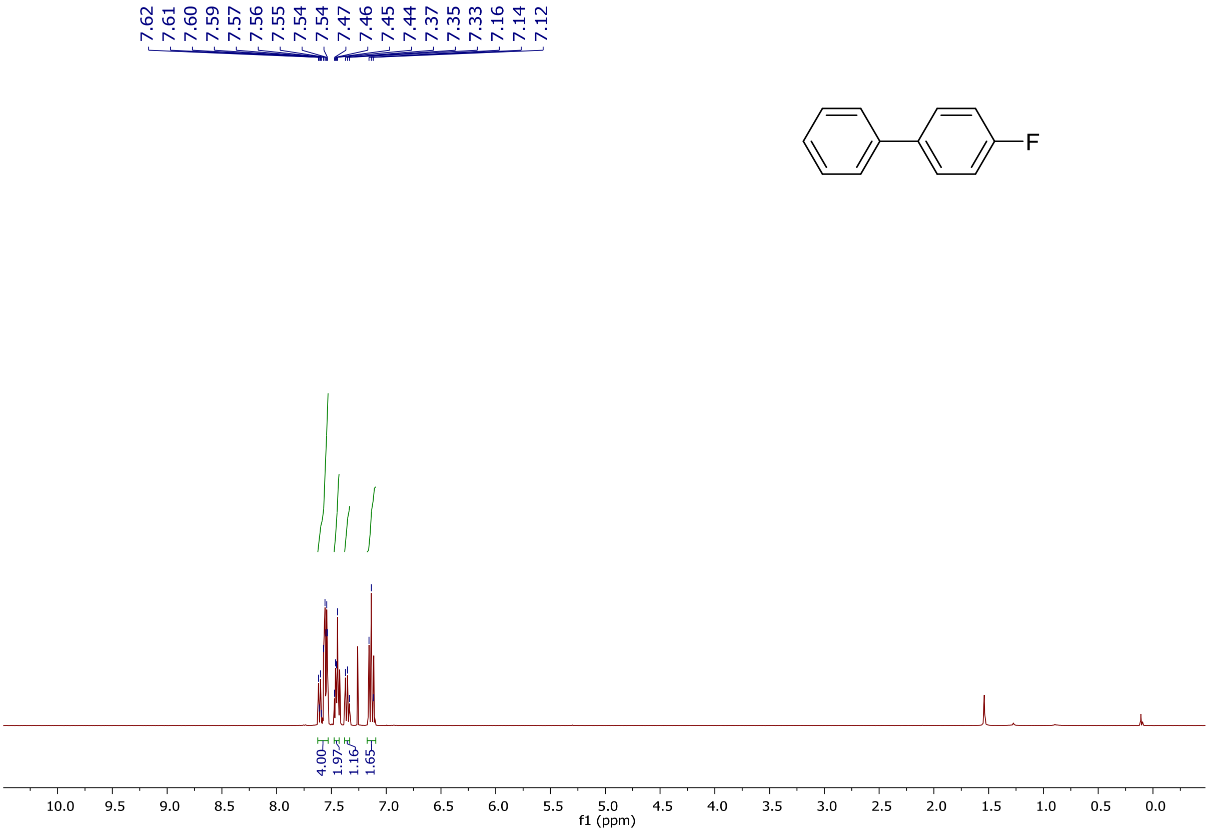

^{13}C NMR of 4-Fluoro-1,1'-biphenyl (Table 2, Entry 3)

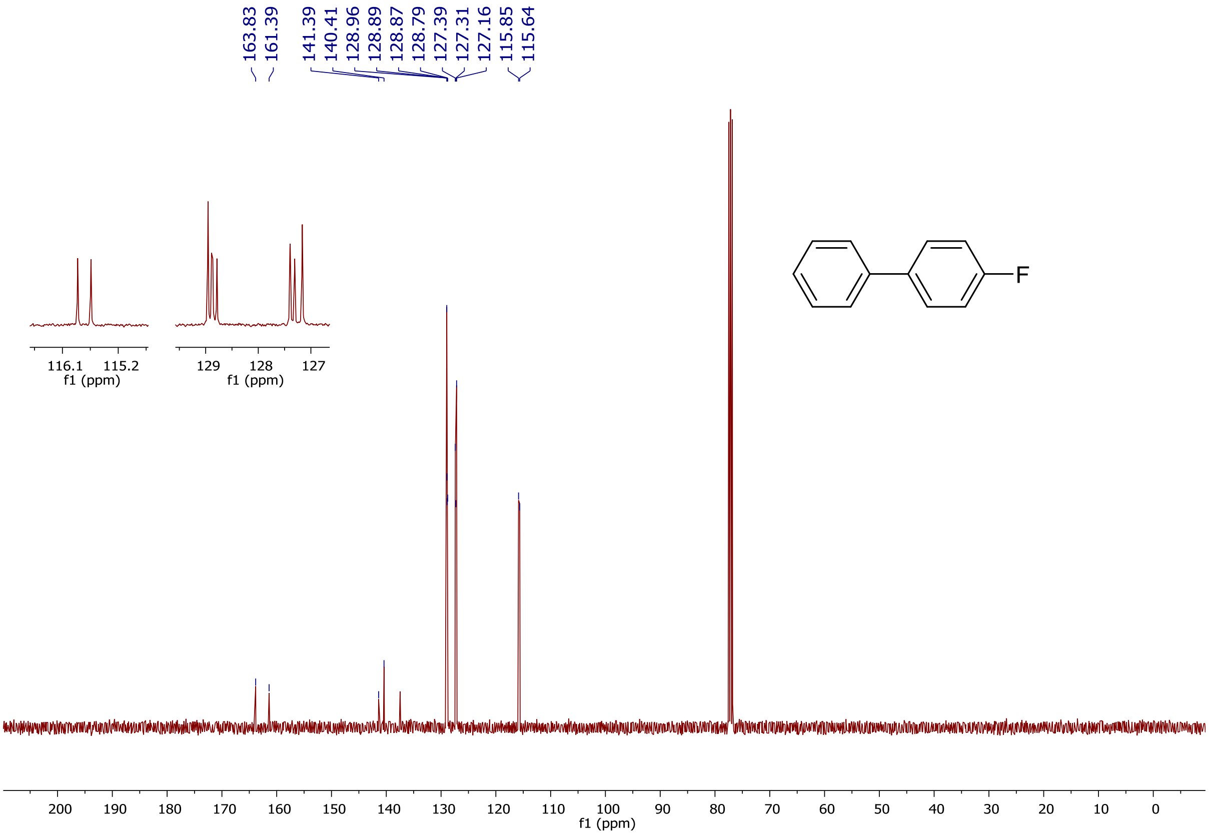

^{1}H NMR of *N*-([1,1'-Biphenyl]-4-yl)acetamide (Table 2, Entry 4)

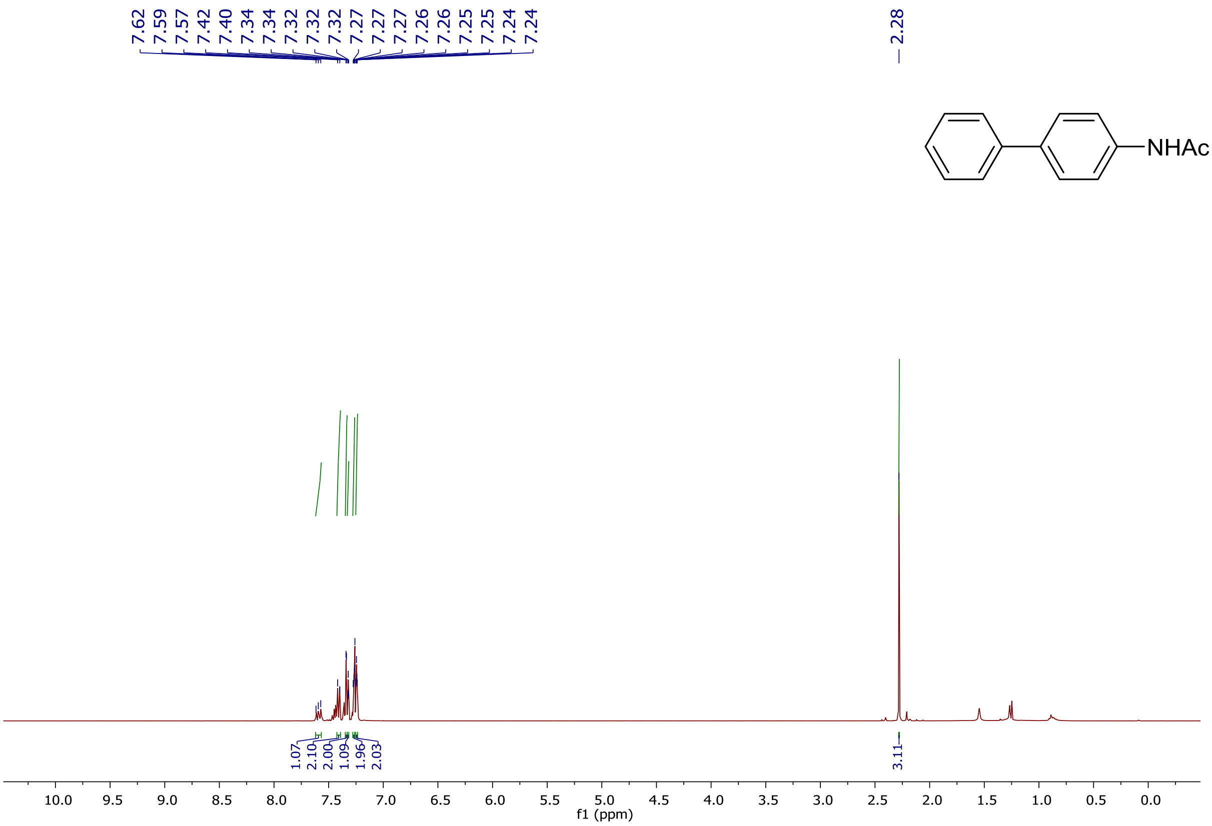

^{13}C NMR of *N*-([1,1'-Biphenyl]-4-yl)acetamide (Table 2, Entry 4)

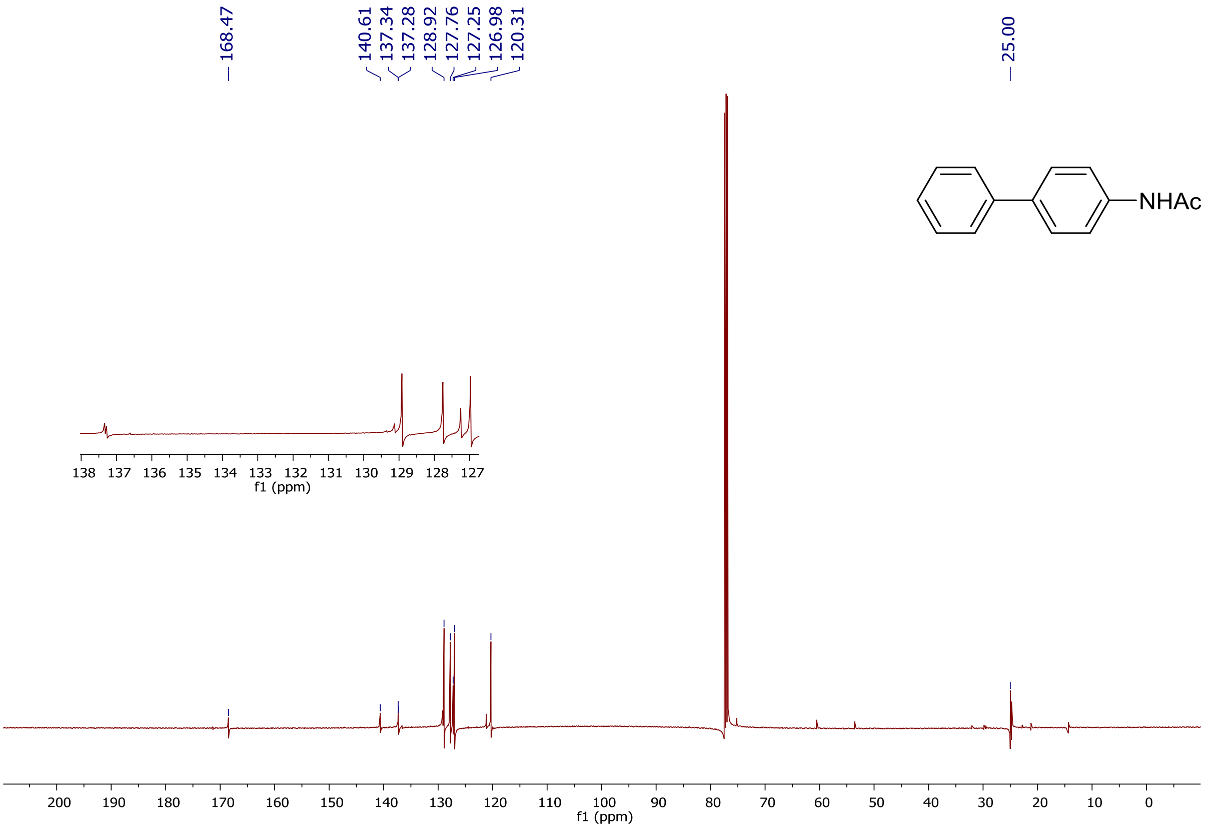

^{1}H NMR of *N*,*N*-Dimethyl-[1,1'-biphenyl]-4-carboxamide (Table 2, Entry 5)

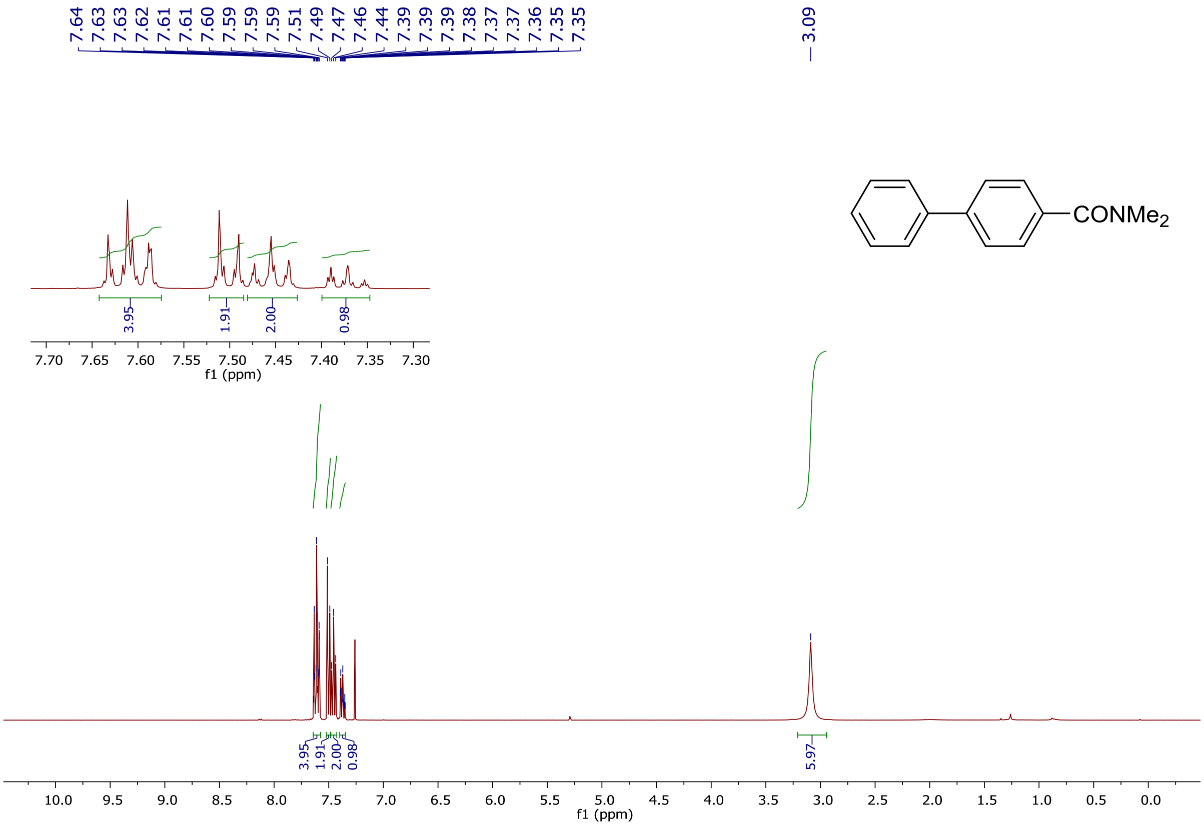

^{13}C NMR of *N*,*N*-Dimethyl-[1,1'-biphenyl]-4-carboxamide (Table 2, Entry 5)

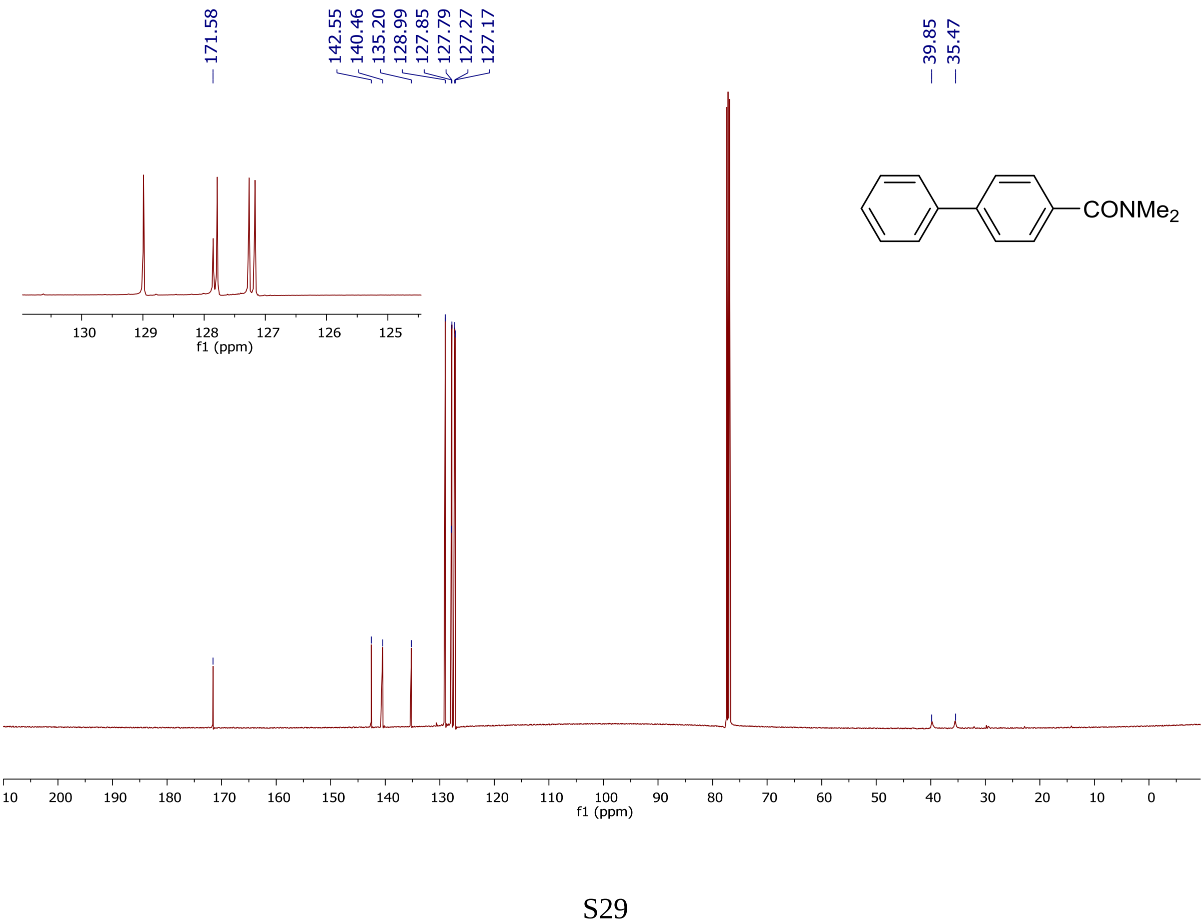

^{1}H NMR of 4-Methyl-1,1'-biphenyl (Table 2, Entry 6)

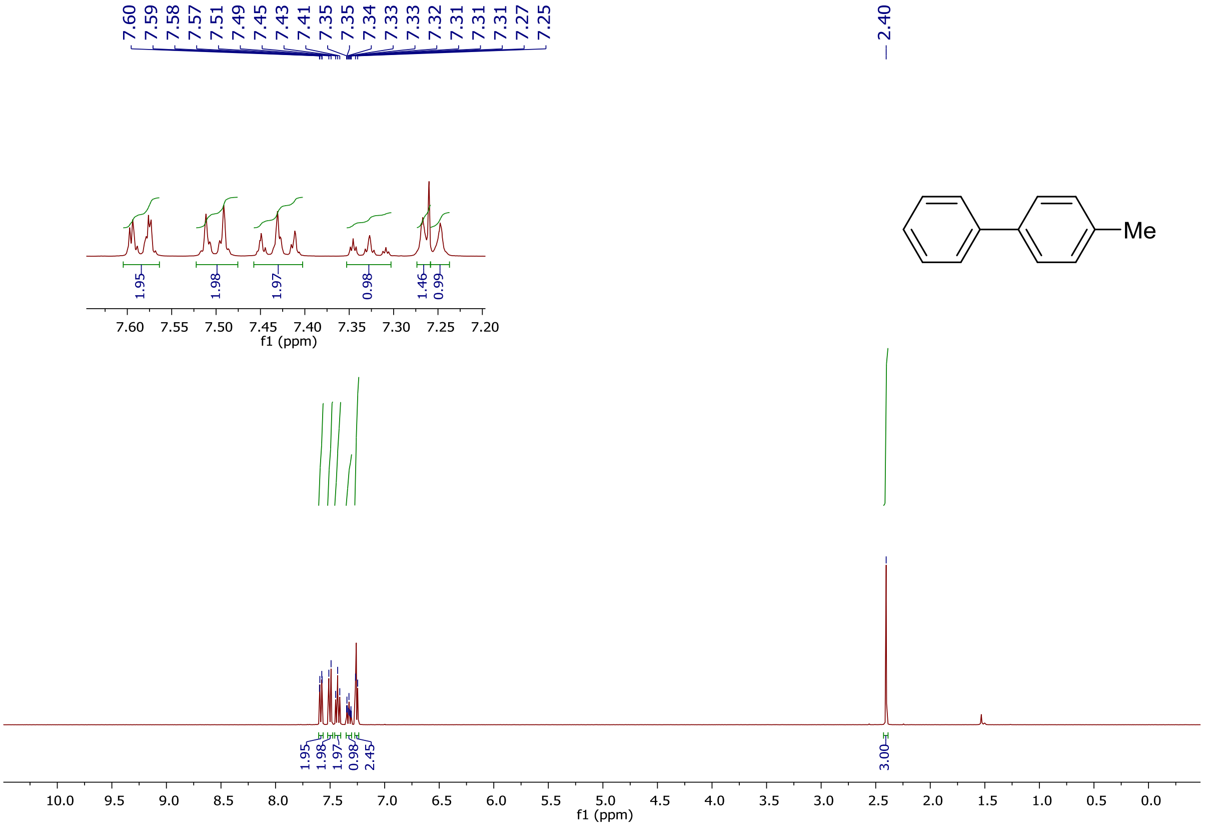

^{13}C NMR of 4-Methyl-1,1'-biphenyl (Table 2, Entry 6)

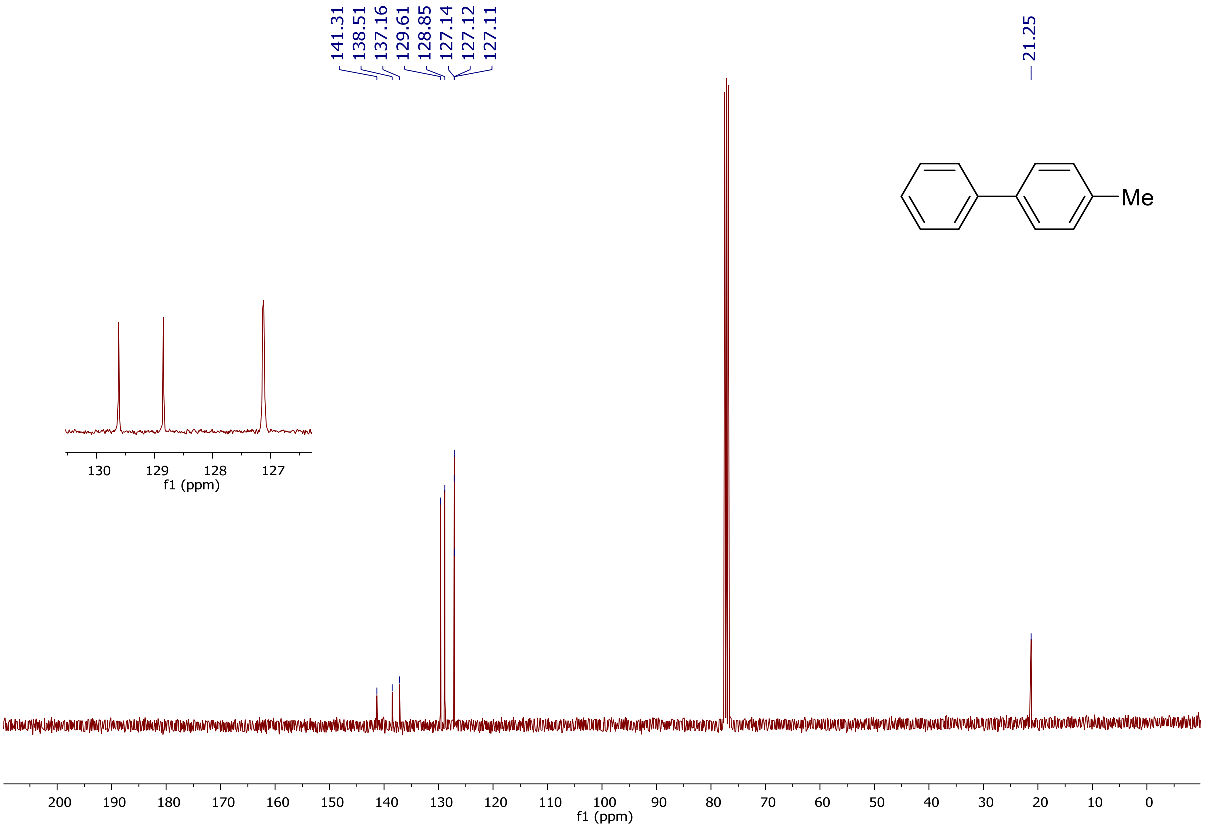

^{1}H NMR of 4-Ethyl-1,1'-biphenyl (Table 2, Entry 7)

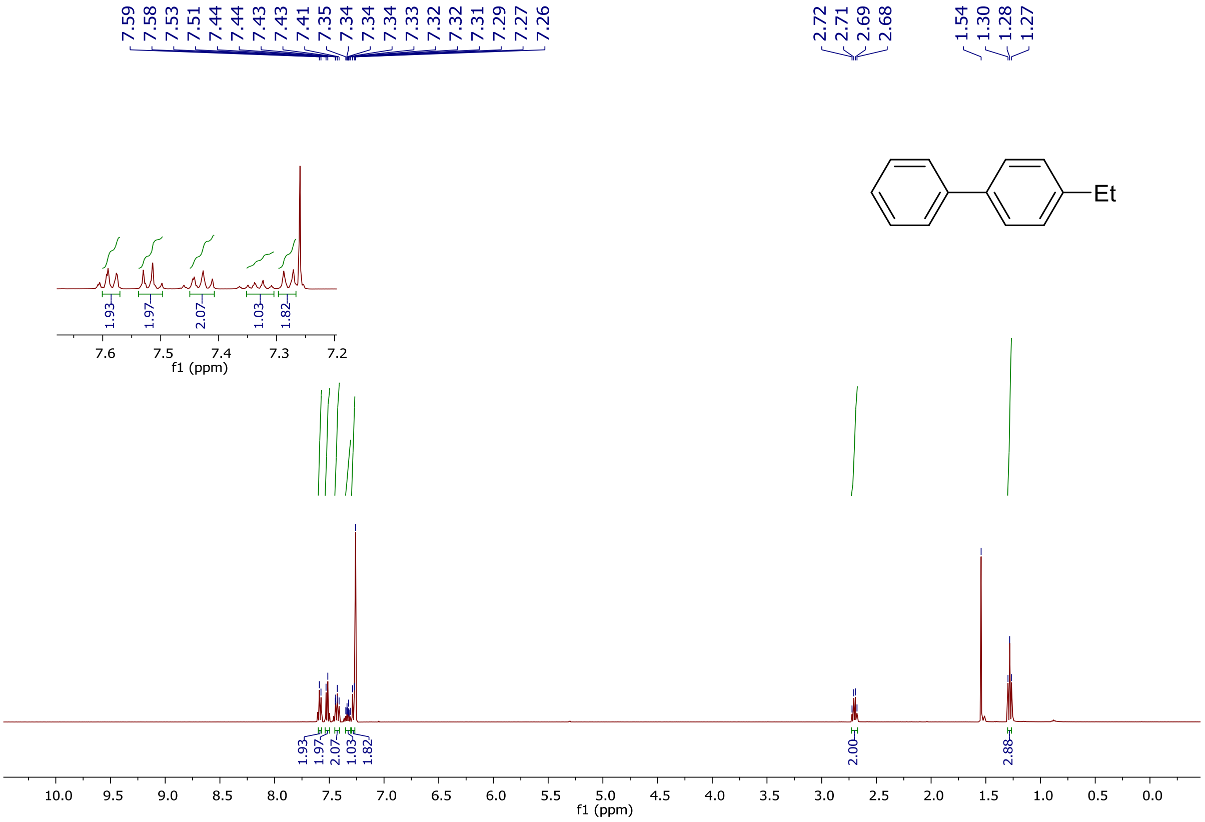

^{13}C NMR of 4-Ethyl-1,1'-biphenyl (Table 2, Entry 7)

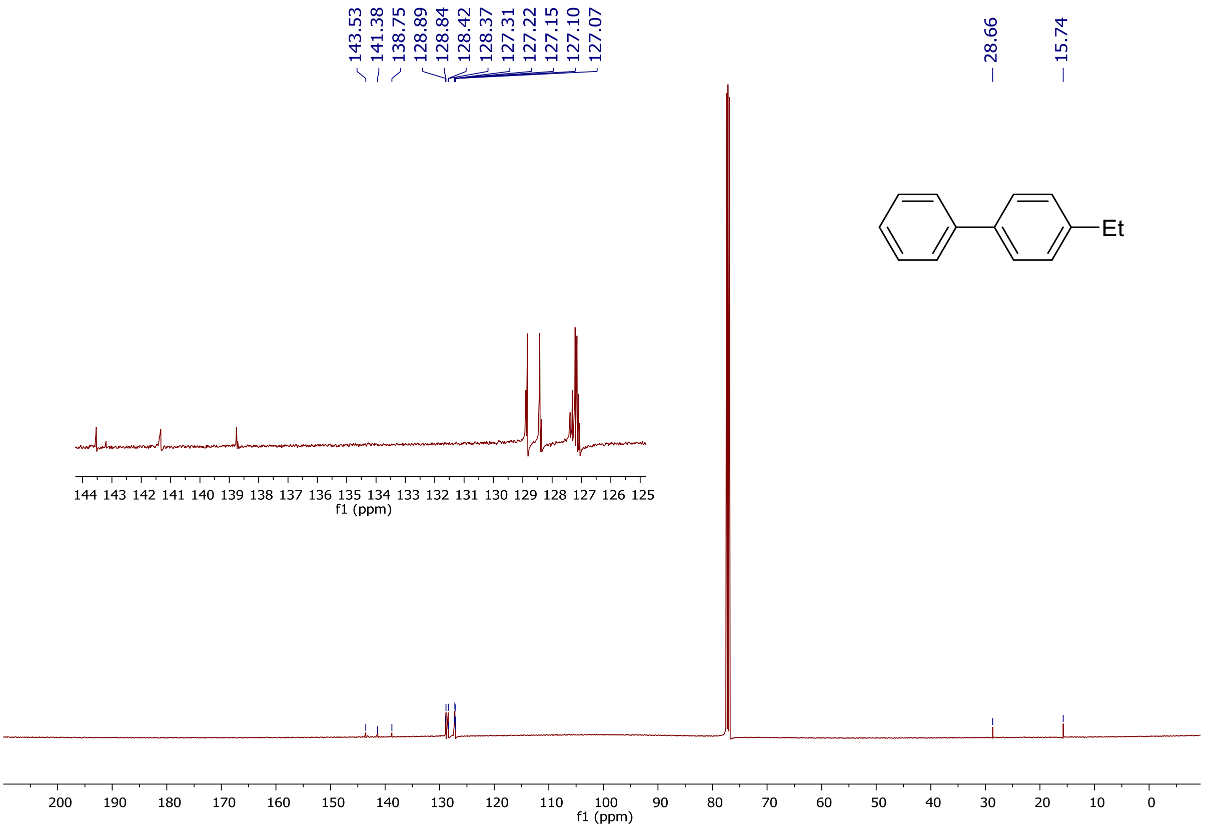

^{1}H NMR of 4-Ethyl-1,1'-biphenyl (Table 2, Entry 8)

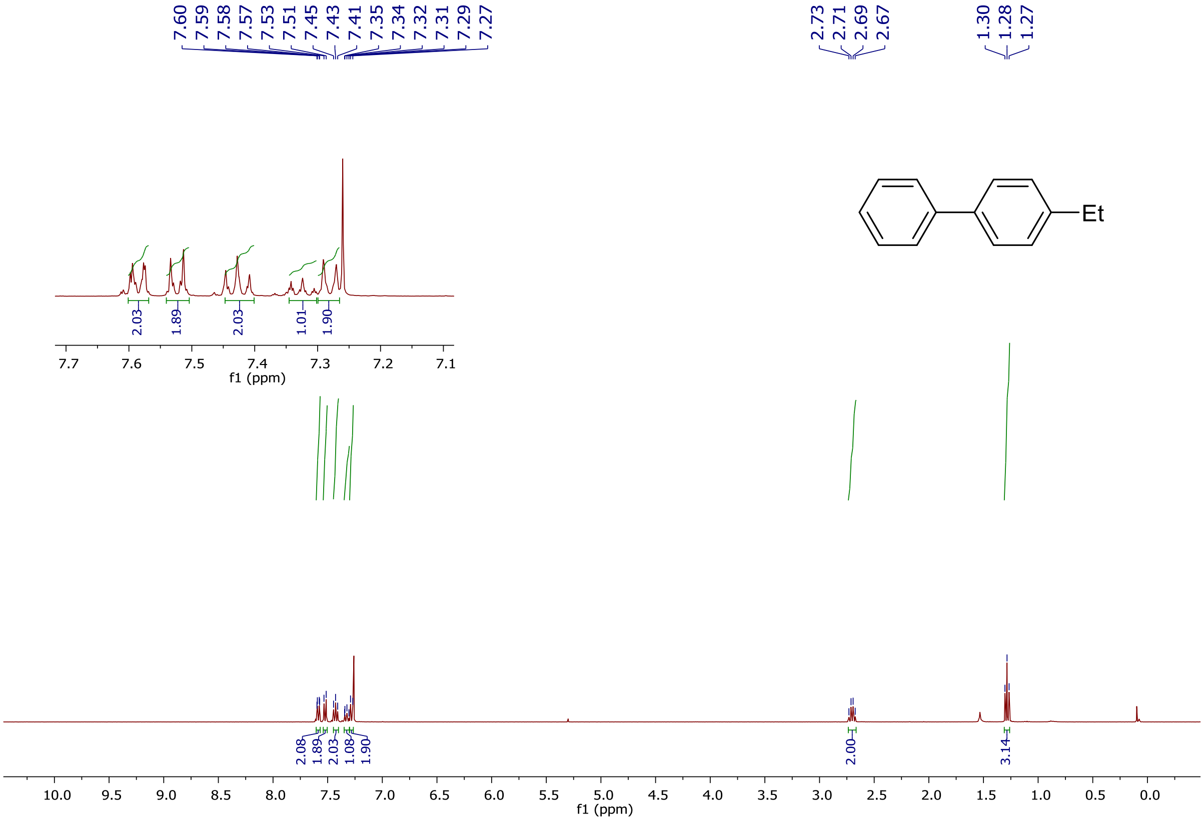

^{13}C NMR of 4-Ethyl-1,1'-biphenyl (Table 2, Entry 8)

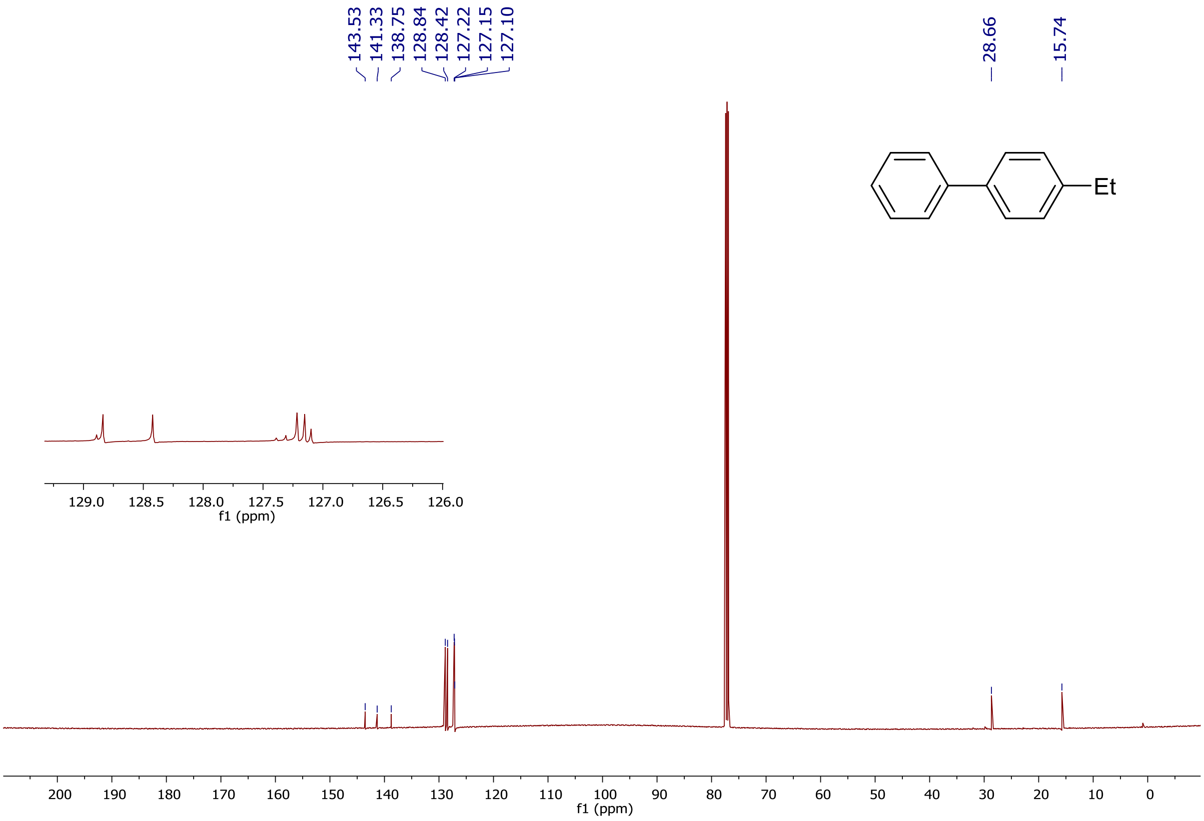

^{1}H NMR of *N*,*N*-Dimethyl-[1,1'-biphenyl]-4-amine (Table 2, Entry 9)

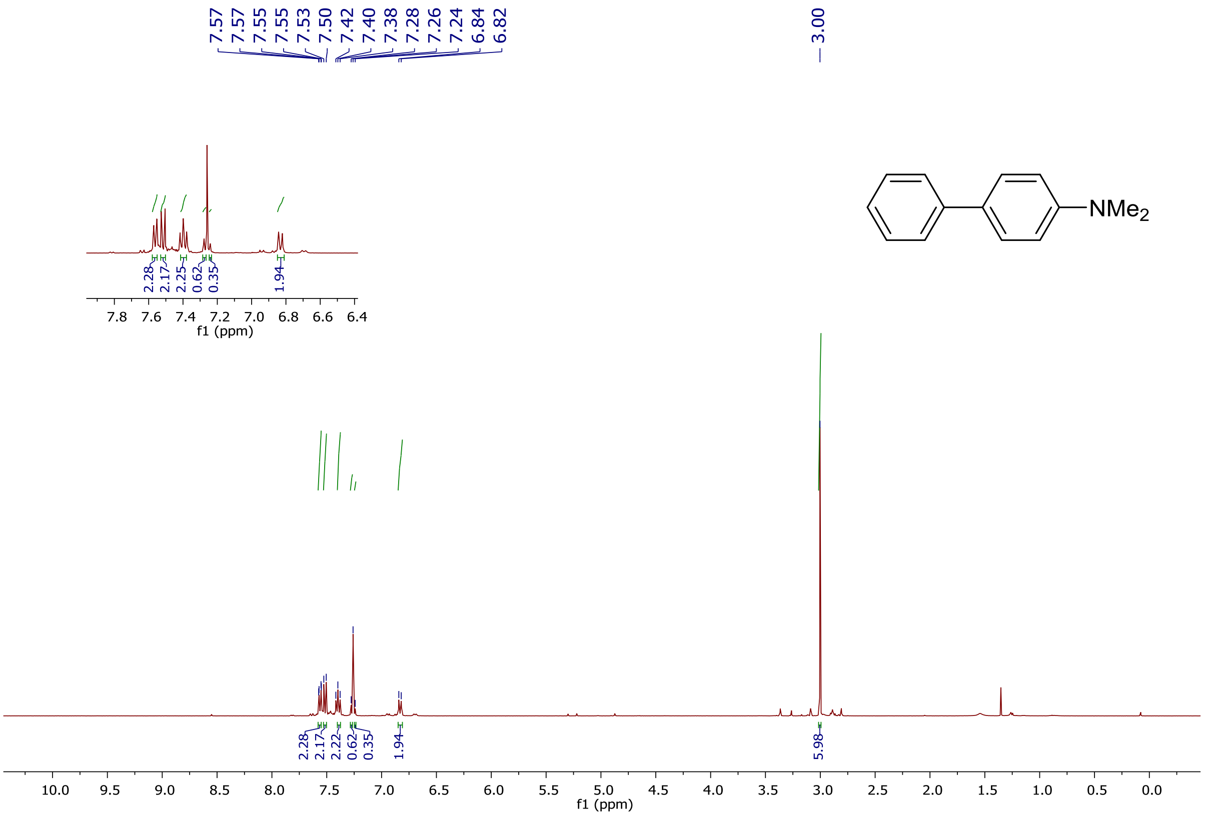

^{13}C NMR of *N*,*N*-Dimethyl-[1,1'-biphenyl]-4-amine (Table 2, Entry 9)

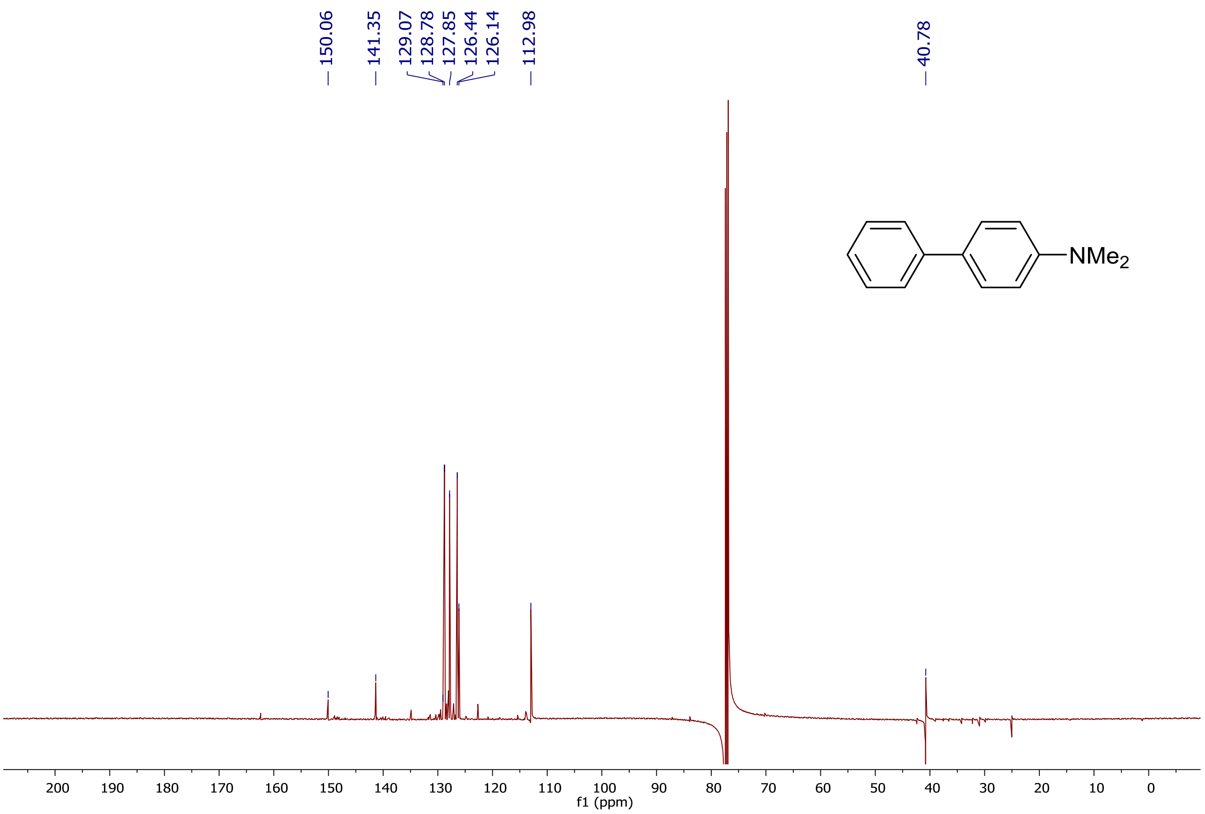

^{1}H NMR of 4-Methoxy-1,1'-biphenyl (Table 2, Entry 10)

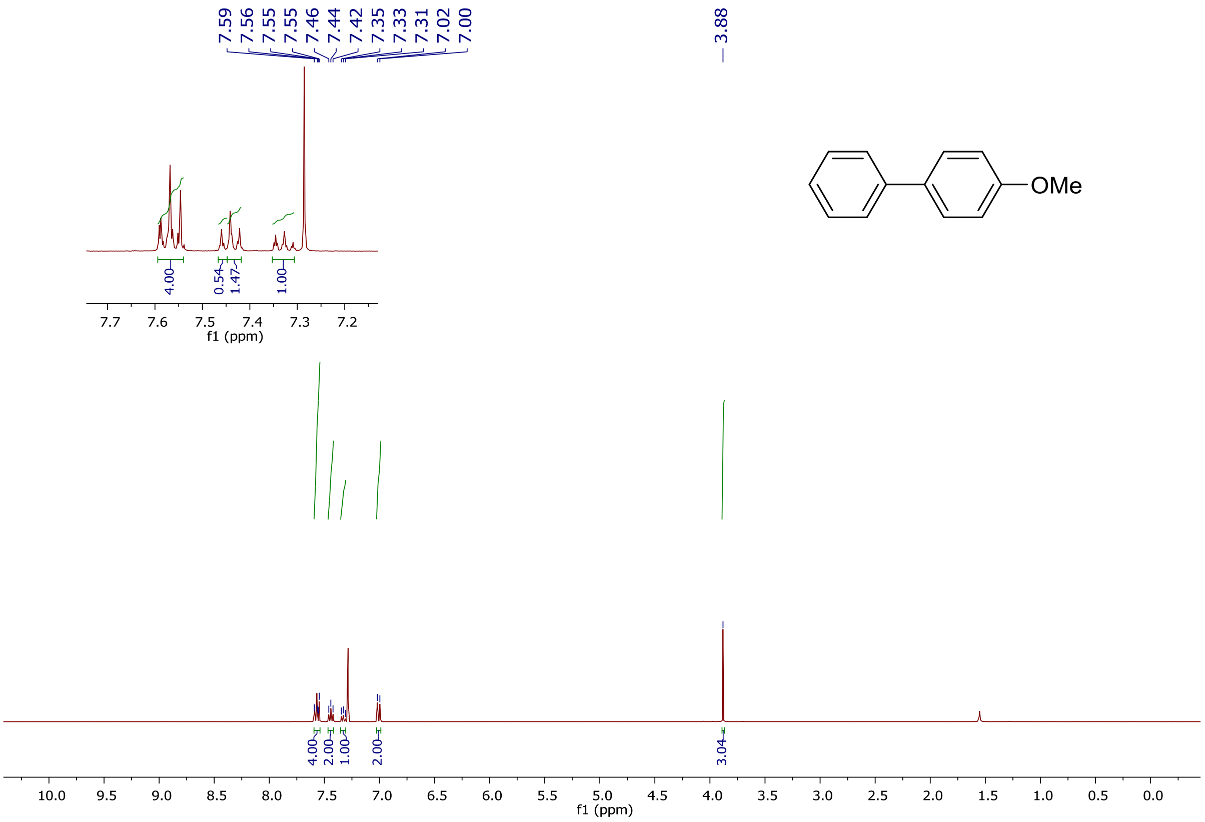

^{13}C NMR of 4-Methoxy-1,1'-biphenyl (Table 2, Entry 10)

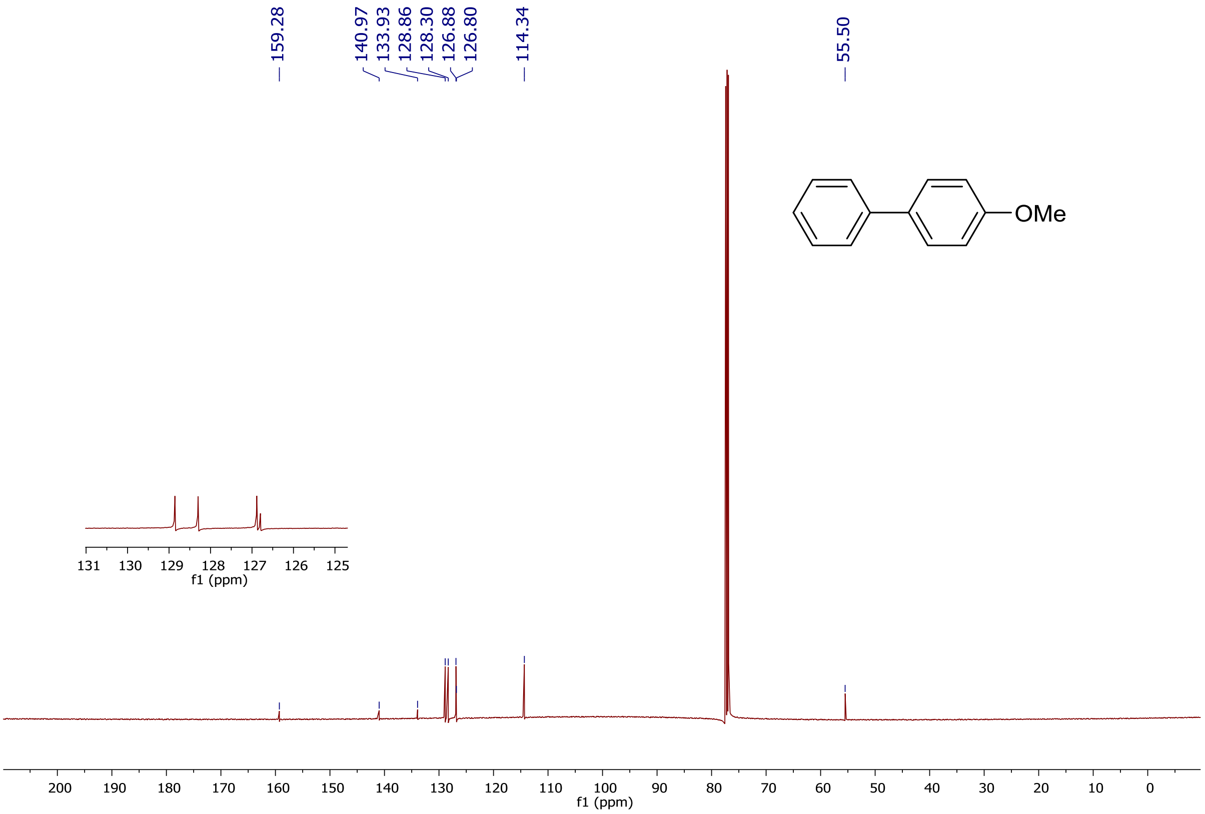

^{1}H NMR of 3-Methyl-1,1'-biphenyl (Table 2, Entry 11)

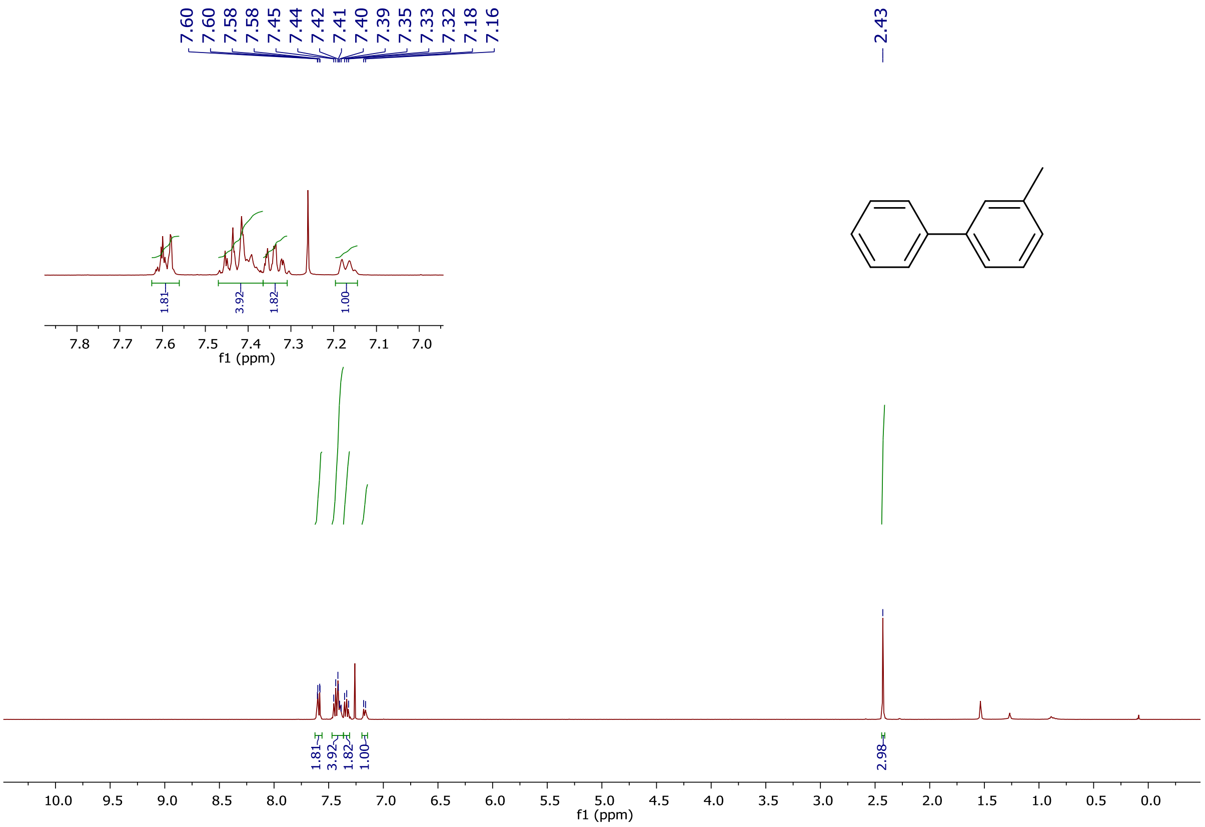

^{13}C NMR of 3-Methyl-1,1'-biphenyl (Table 2, Entry 11)

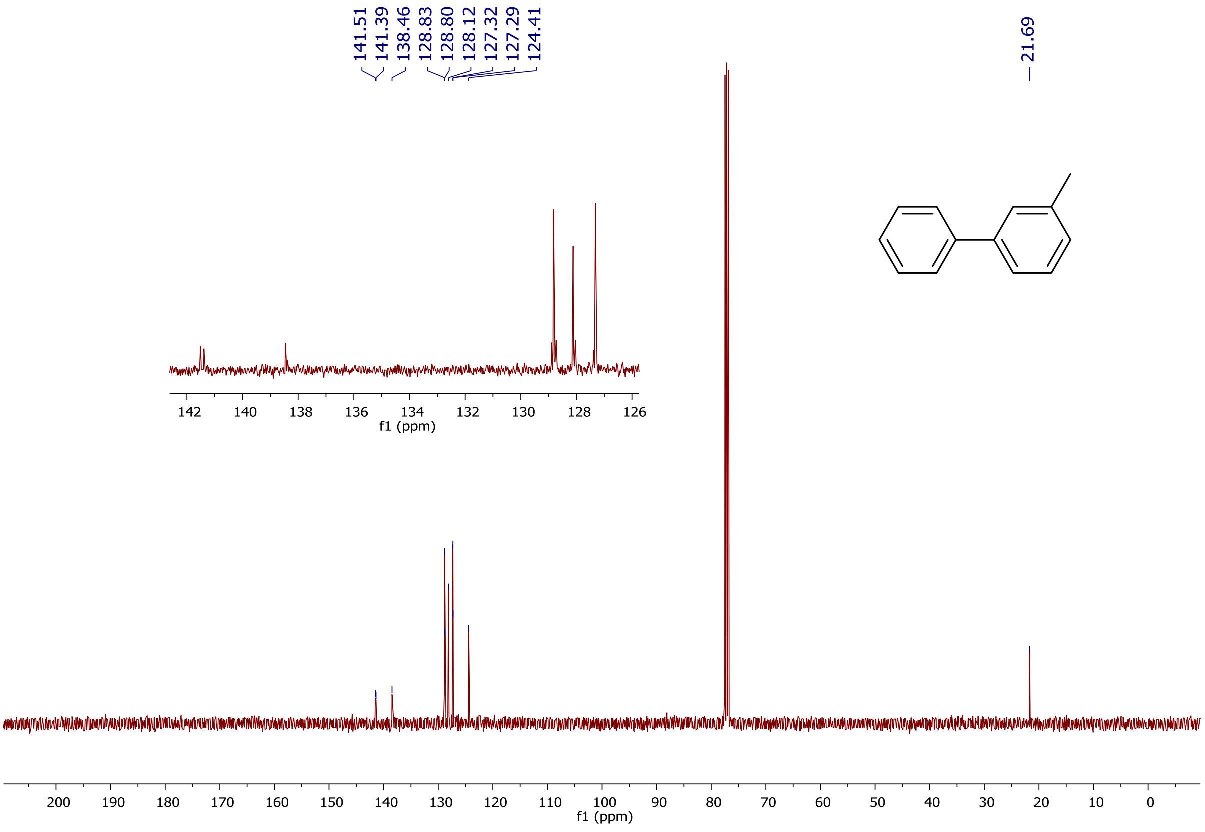

^{1}H NMR of 3,5-Dimethyl-1,1'-biphenyl (Table 2, Entry 12)

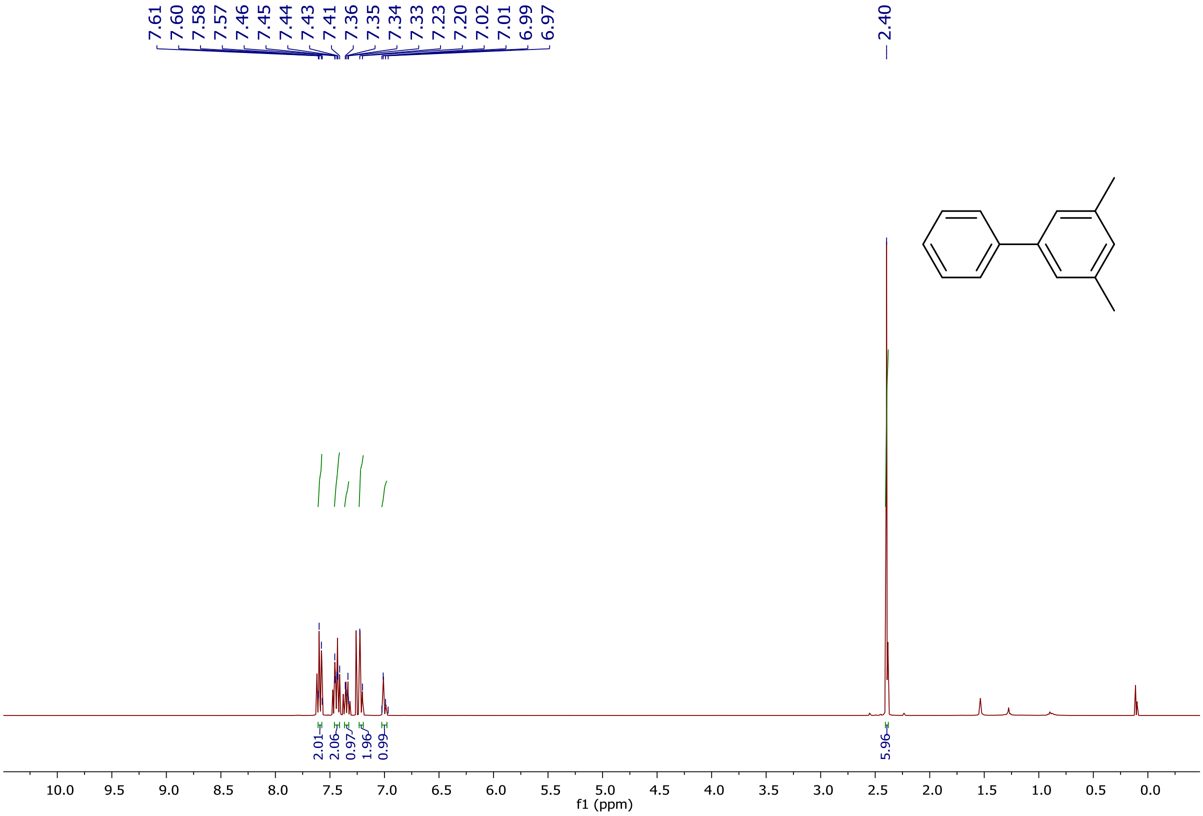

^{13}C NMR of 3,5-Dimethyl-1,1'-biphenyl (Table 2, Entry 12)

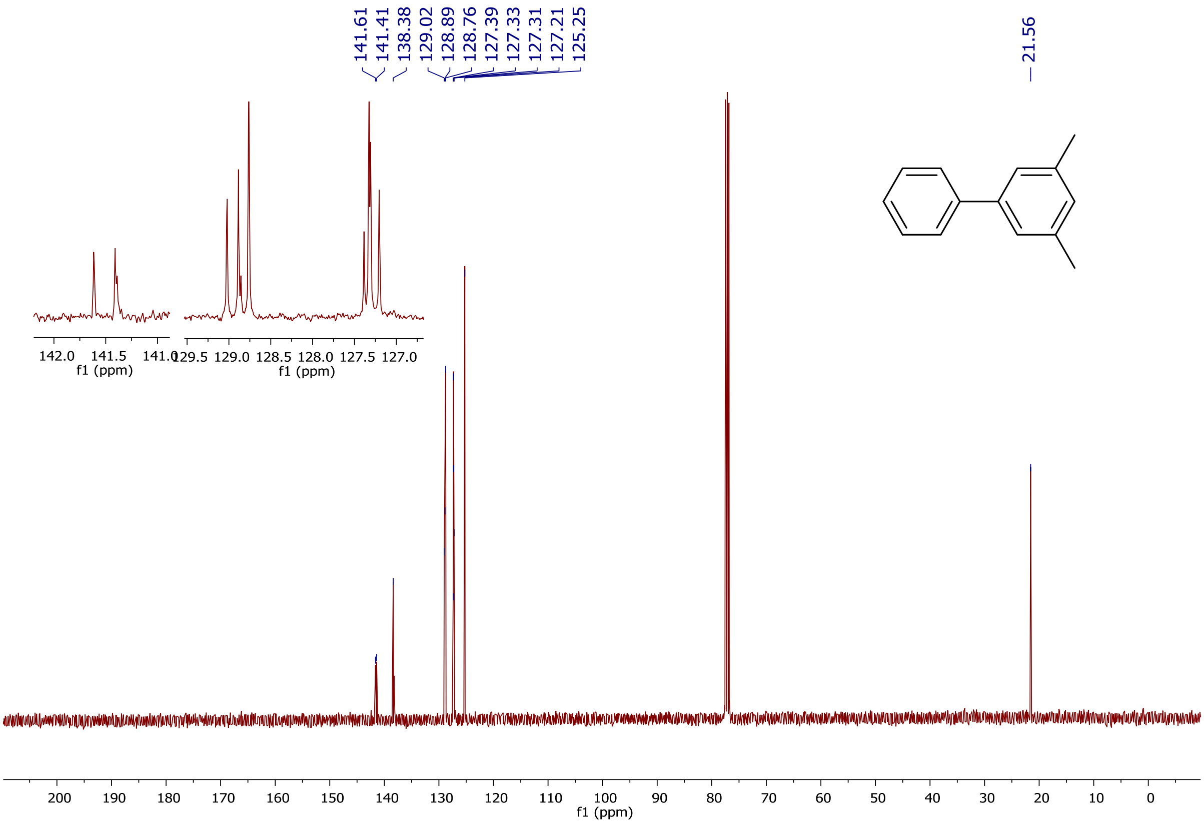

^{1}H NMR of 2-Methyl-1,1'-biphenyl (Table 2, Entry 13)

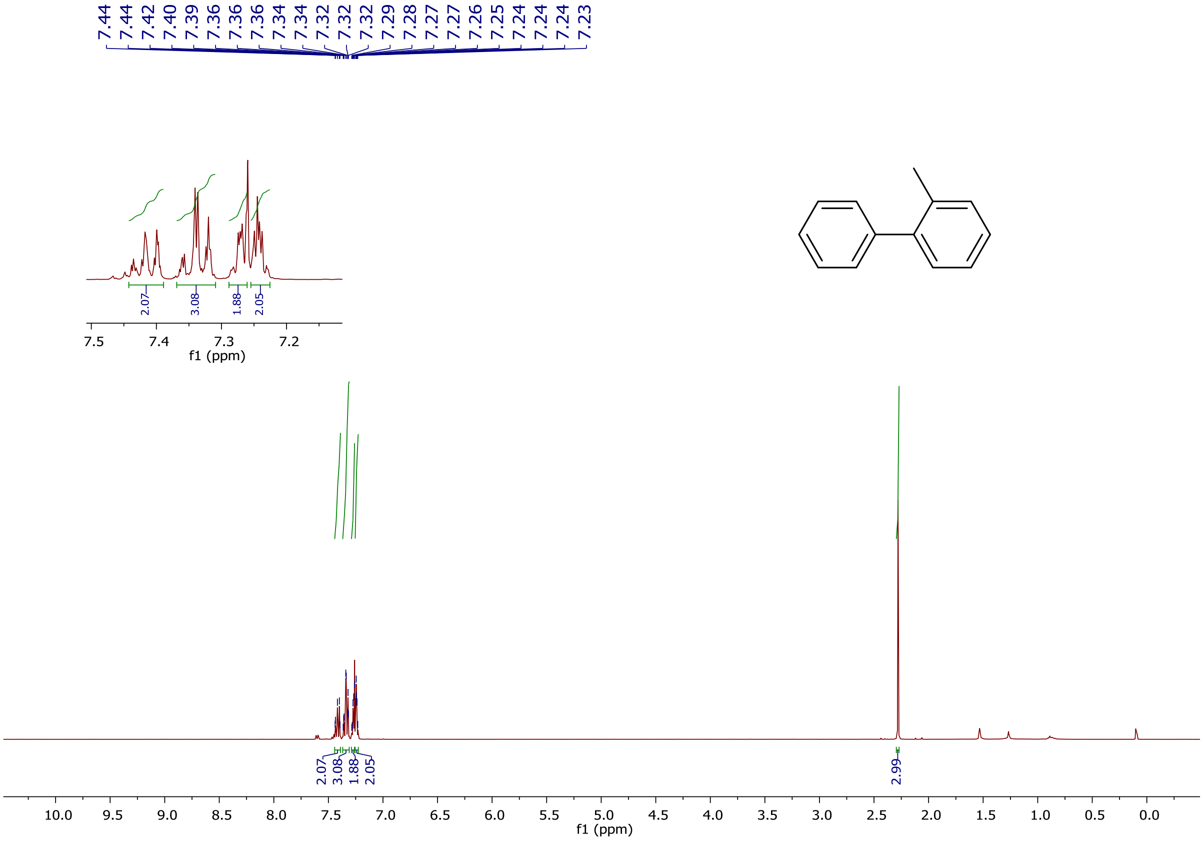

^{13}C NMR of 2-Methyl-1,1'-biphenyl (Table 2, Entry 13)

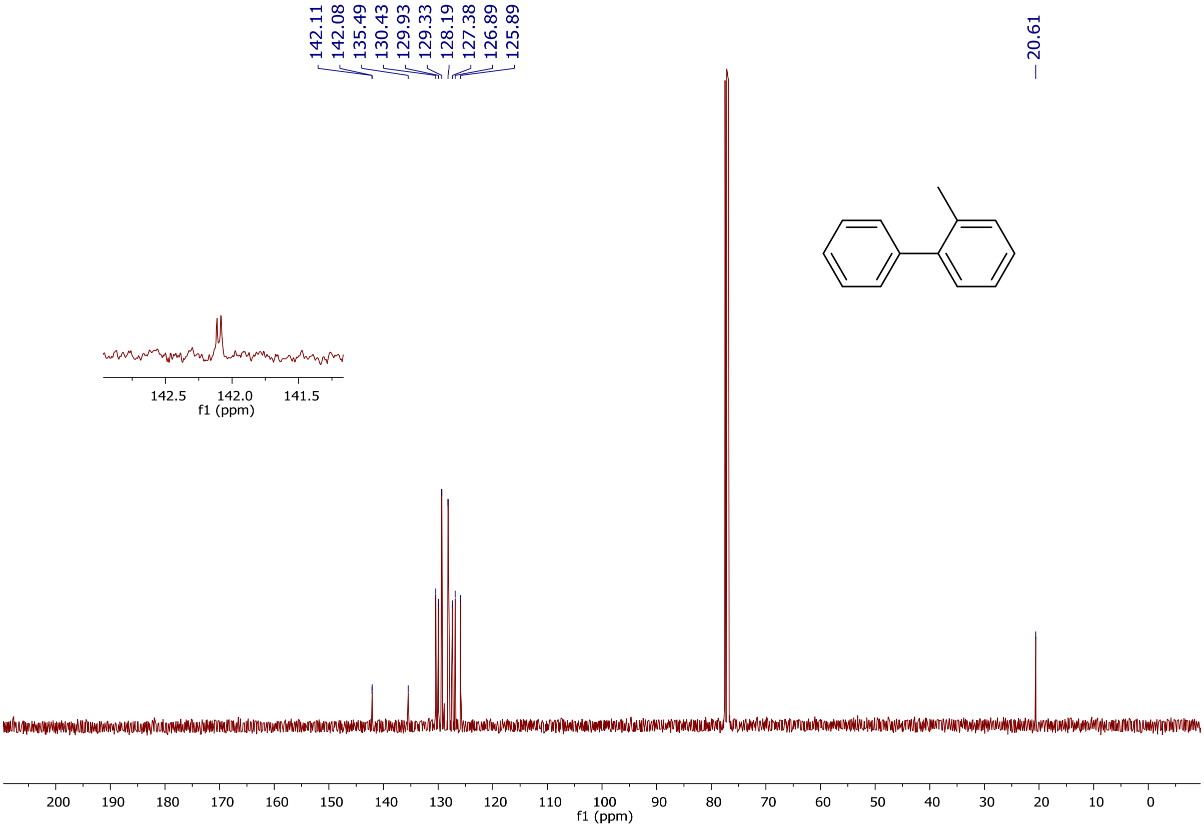

^{1}H NMR of 2-Methoxy-1,1'-biphenyl (Table 2, Entry 14)

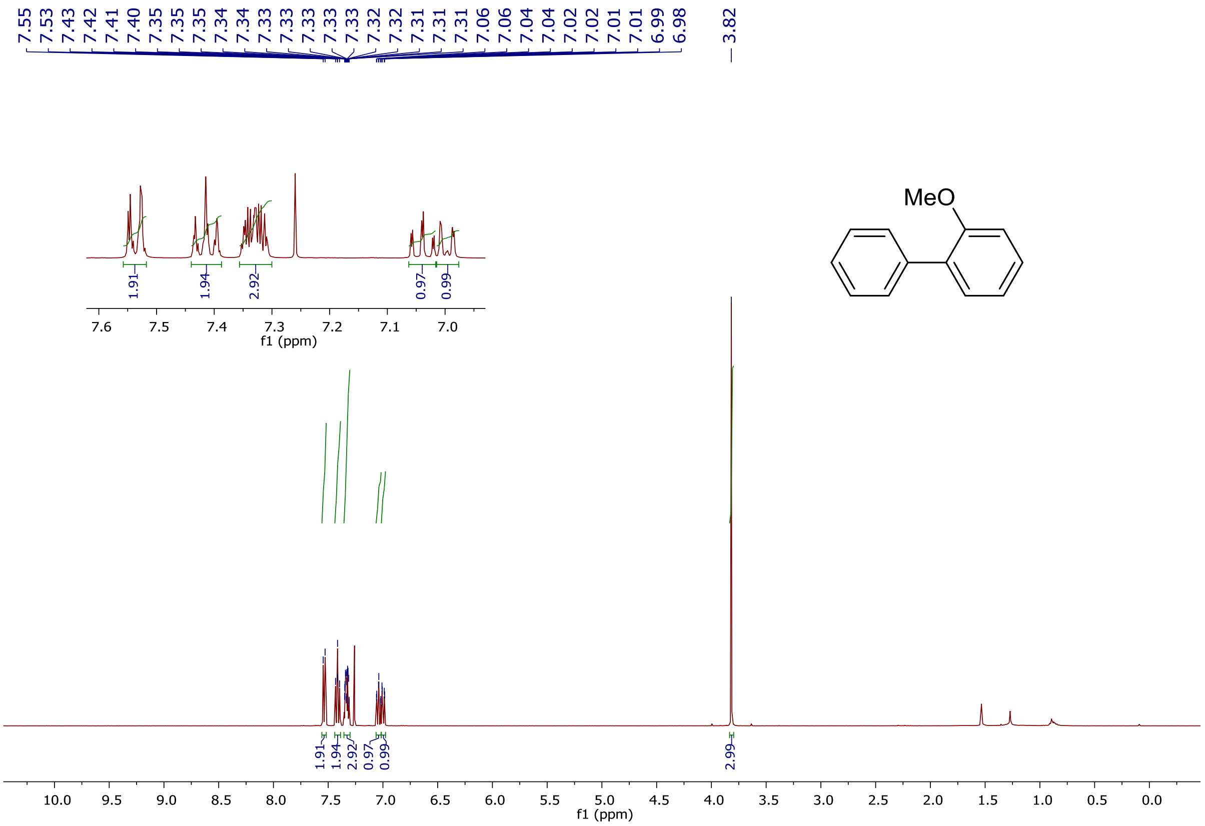

^{13}C NMR of 2-Methoxy-1,1'-biphenyl (Table 2, Entry 14)

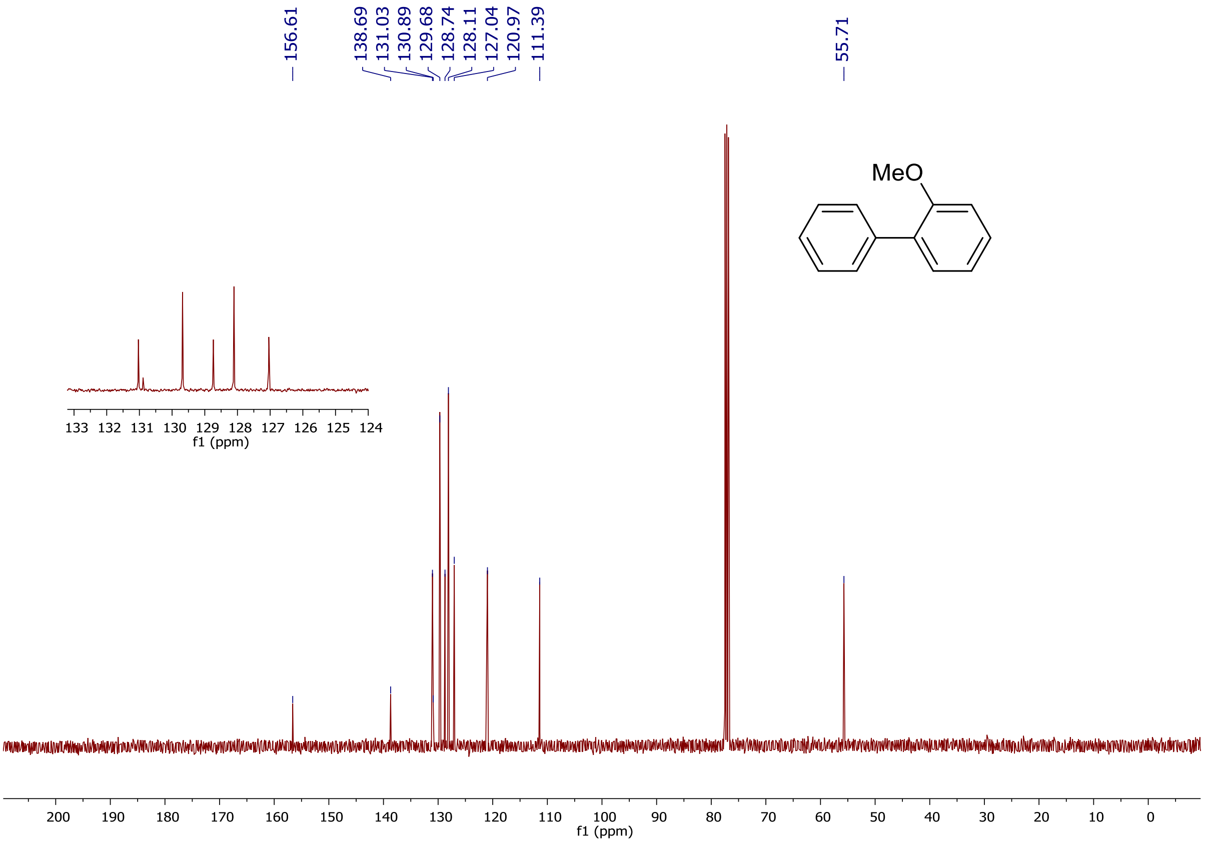

^{1}H NMR of 1-Phenylnaphthalene (Table 2, Entry 15)

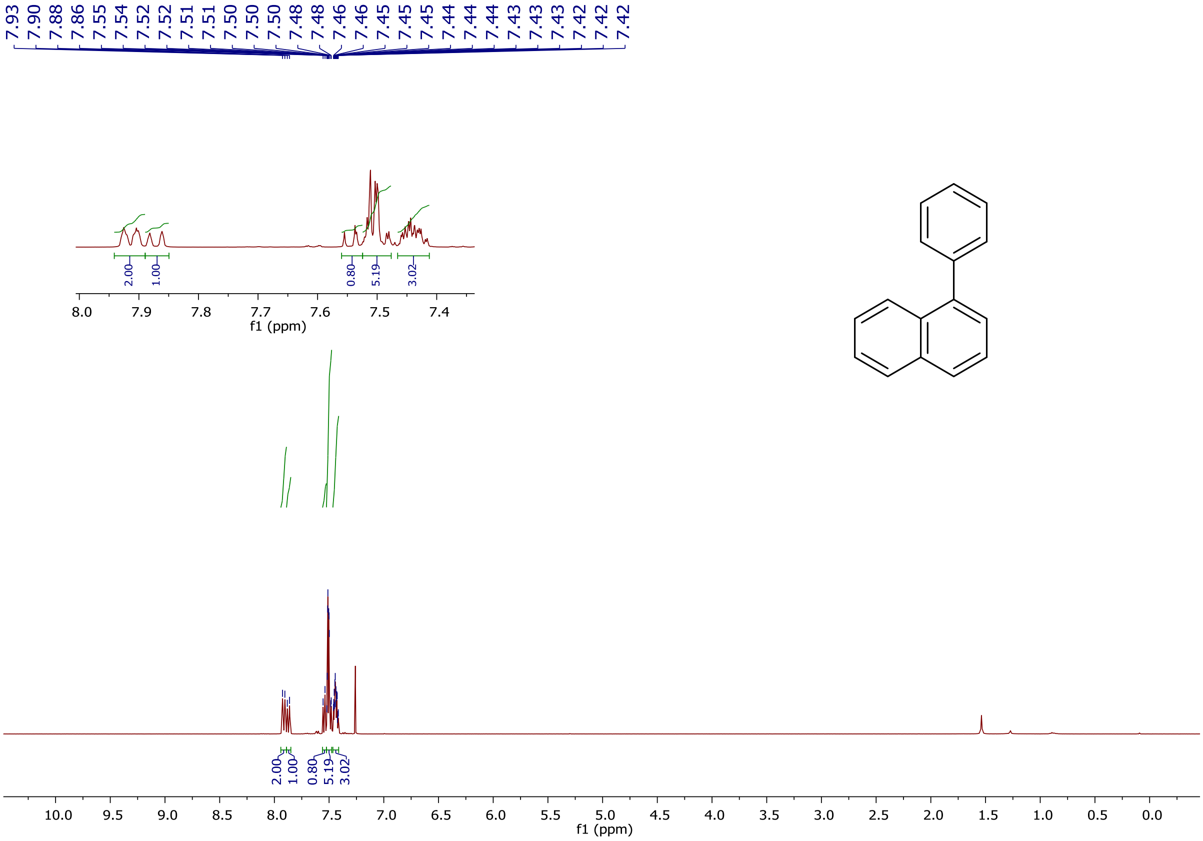

^{13}C NMR of 1-Phenylnaphthalene (Table 2, Entry 15)

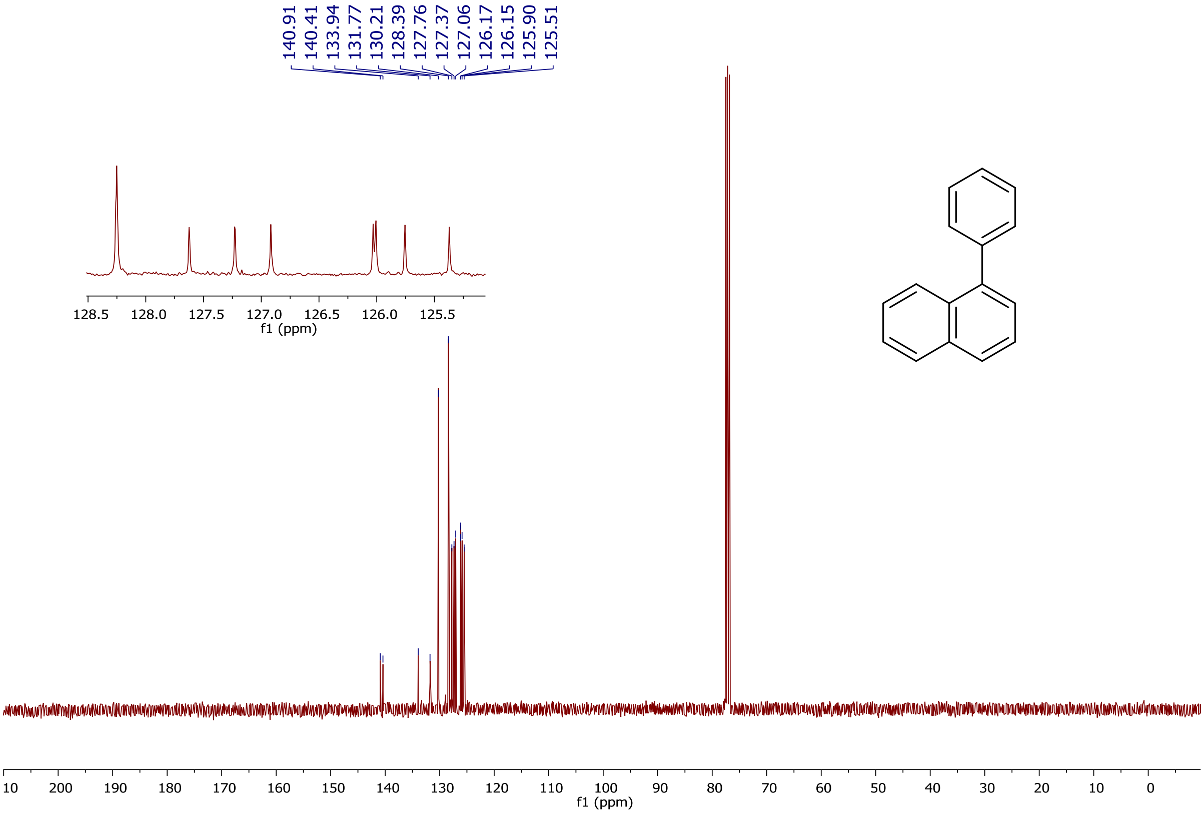

^{1}H NMR of *N*,*N*-Diethyl-[1,1'-biphenyl]-2-carboxamide (Table 2, Entry 16)

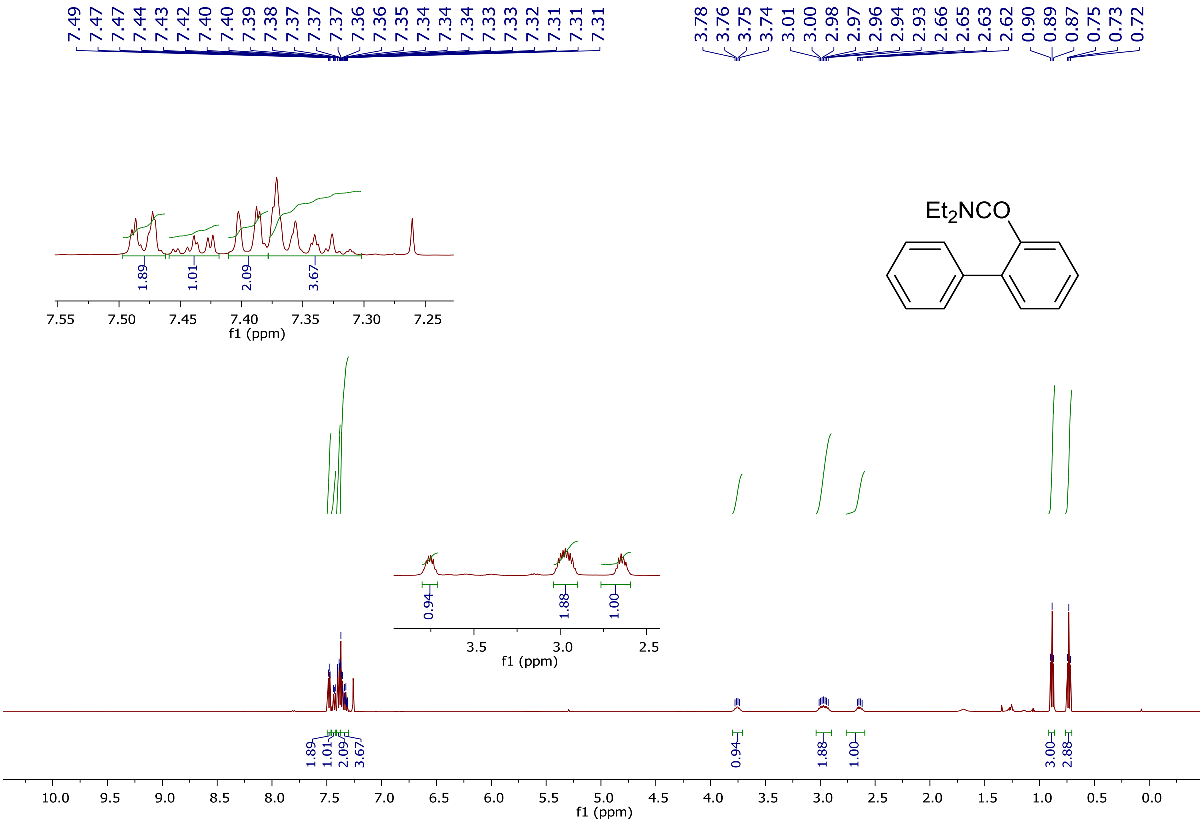

^{13}C NMR of *N*,*N*-Diethyl-[1,1'-biphenyl]-2-carboxamide (Table 2, Entry 16)

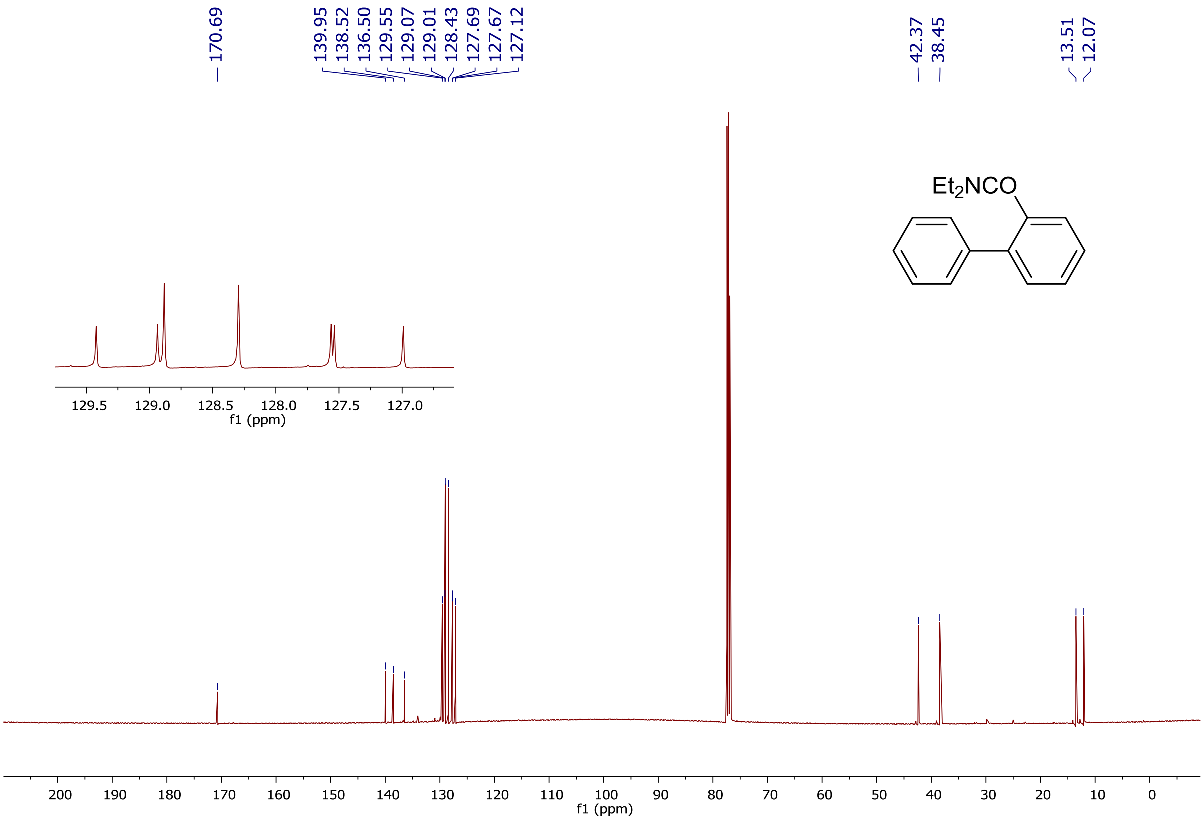

^{1}H NMR of 4-Methoxy-4'-(trifluoromethyl)-1,1'-biphenyl (Table 2, Entry 17)

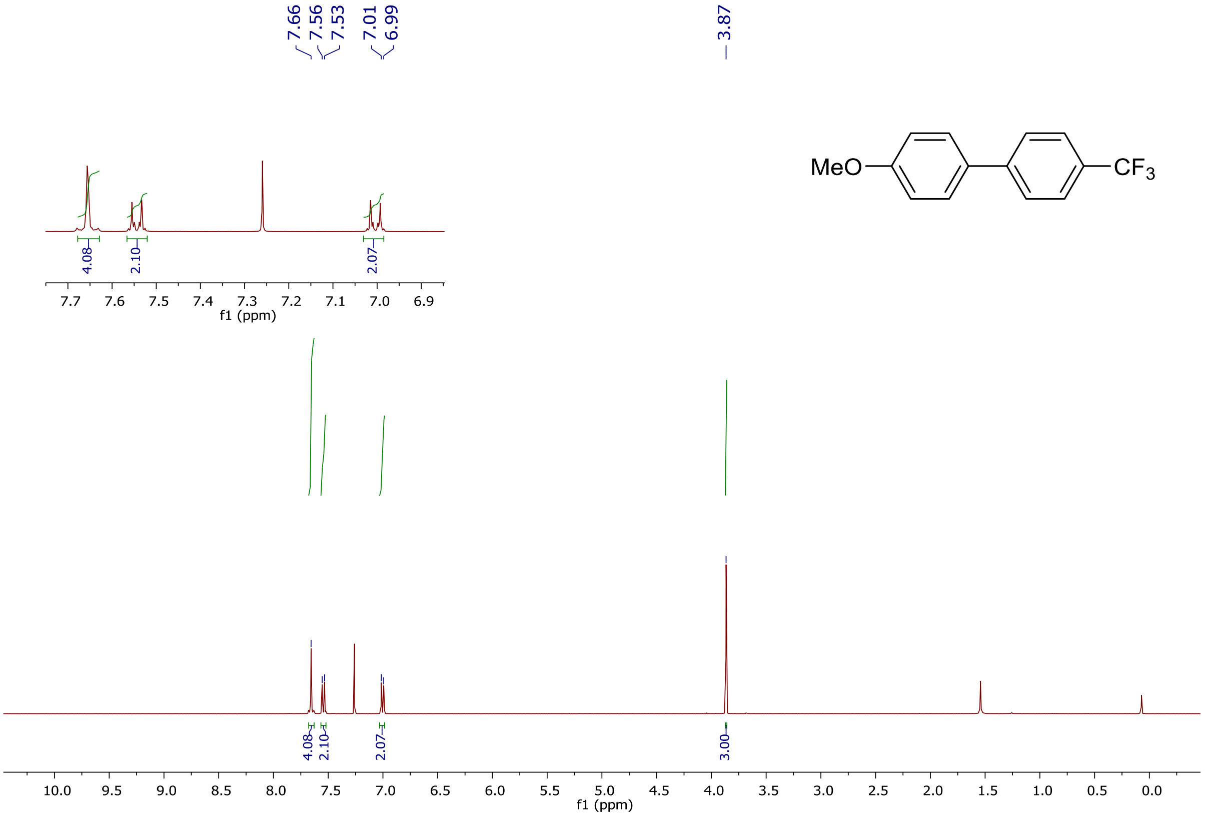

^{13}C NMR of 4-Methoxy-4'-(trifluoromethyl)-1,1'-biphenyl (Table 2, Entry 17

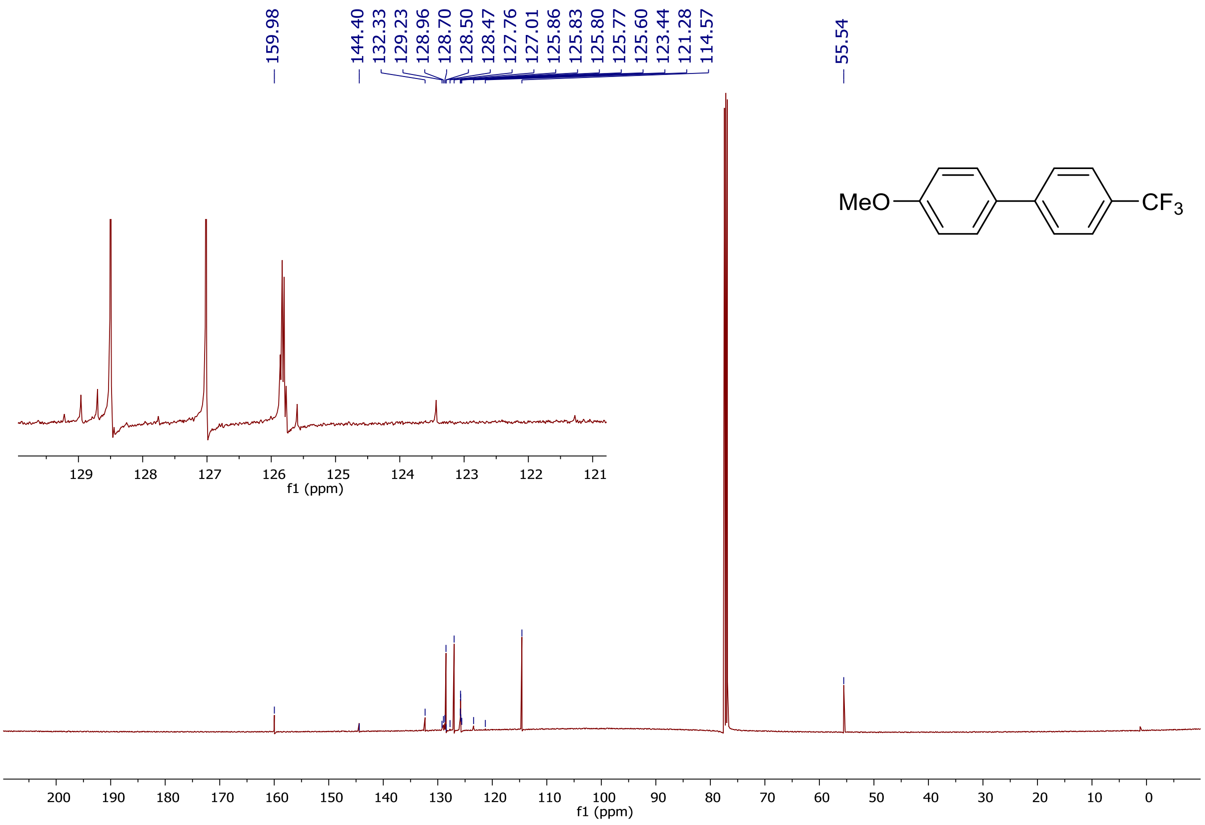

^{1}H NMR of 4-Ethyl-4'-(trifluoromethyl)-1,1'-biphenyl (Table 2, Entry 18)

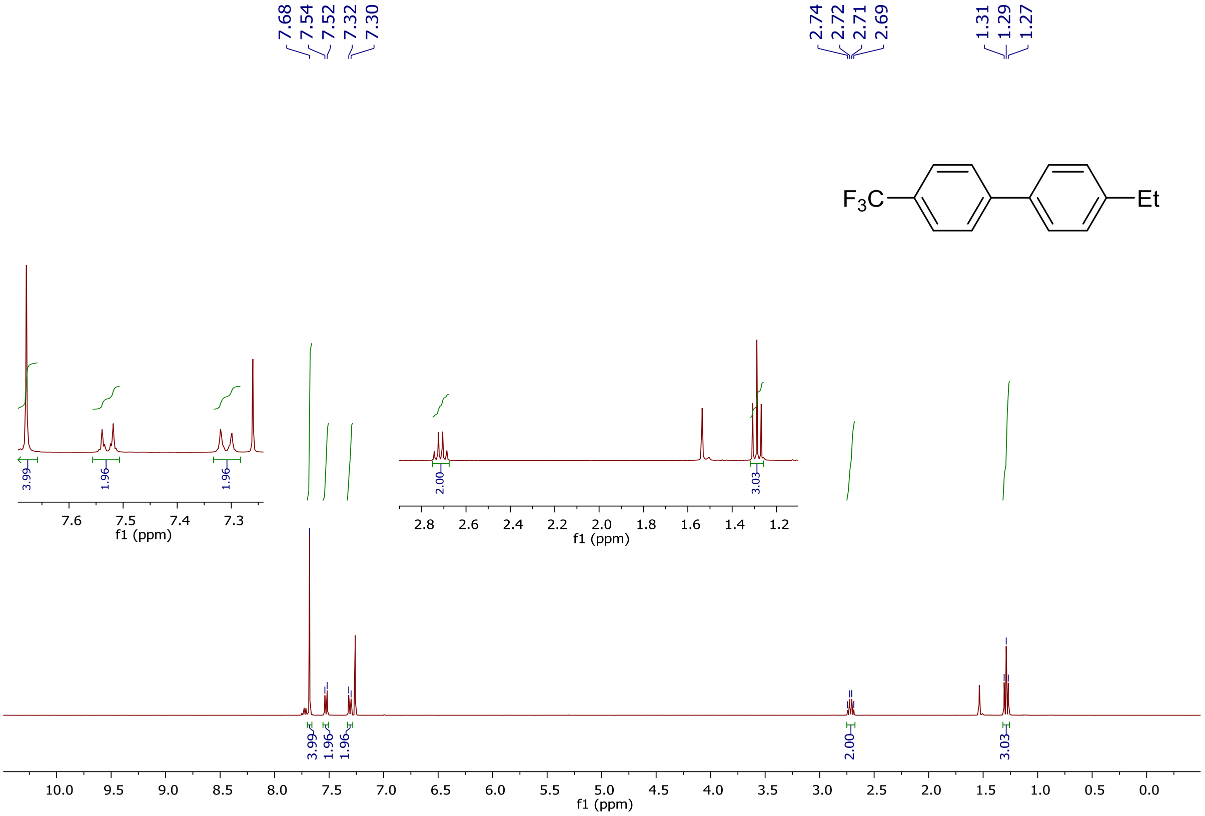

^{13}C NMR of 4-Ethyl-4'-(trifluoromethyl)-1,1'-biphenyl (Table 2, Entry 18)

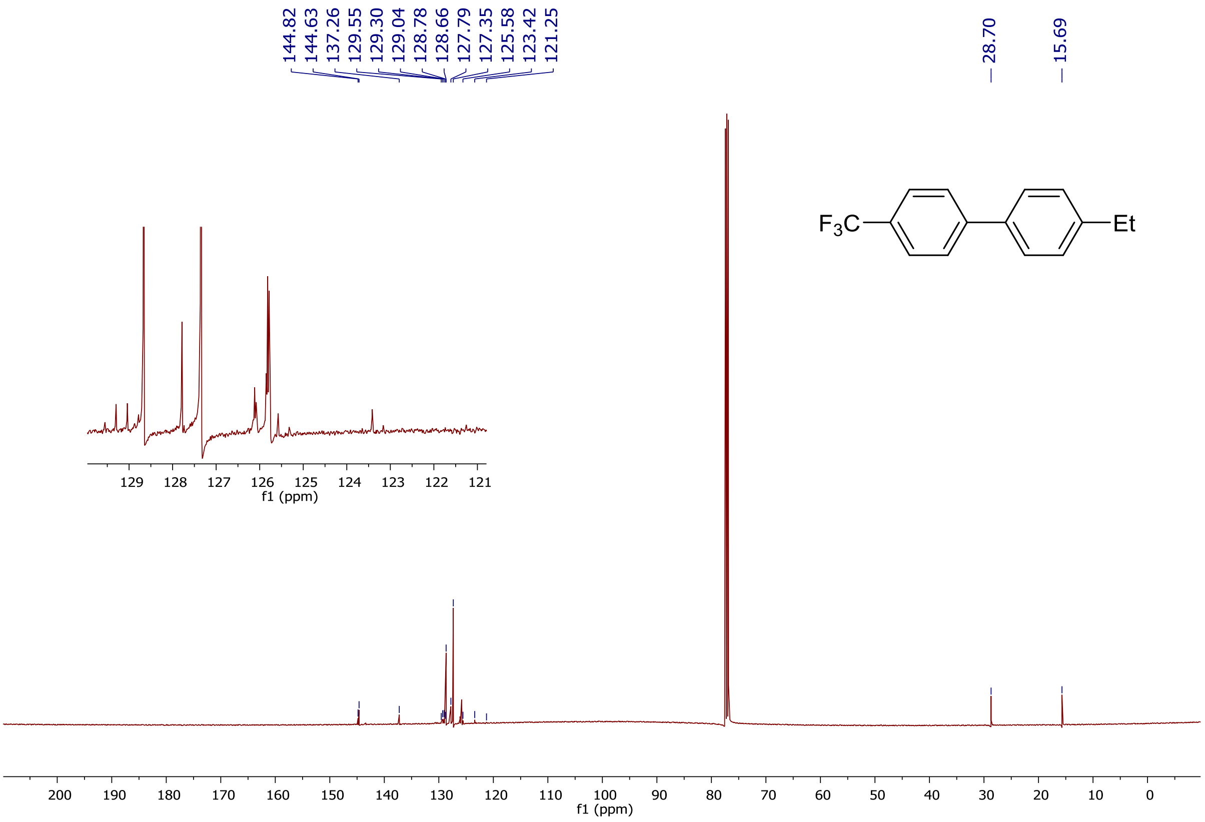

^{1}H NMR of 4-(4'-Methyl-[1,1'-biphenyl]-4-yl)morpholine (Table 2, Entry 19)

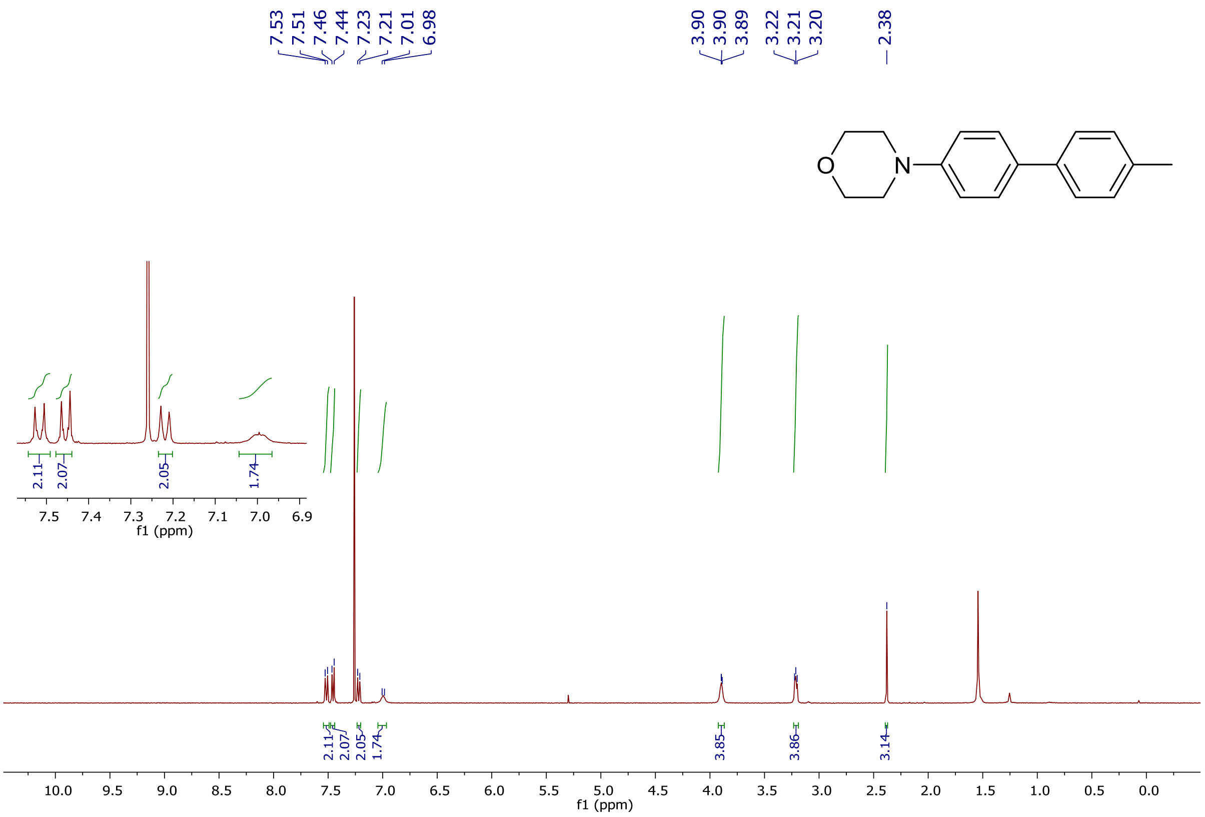

^{13}C NMR of 4-(4'-Methyl-[1,1'-biphenyl]-4-yl)morpholine (Table 2, Entry 19)

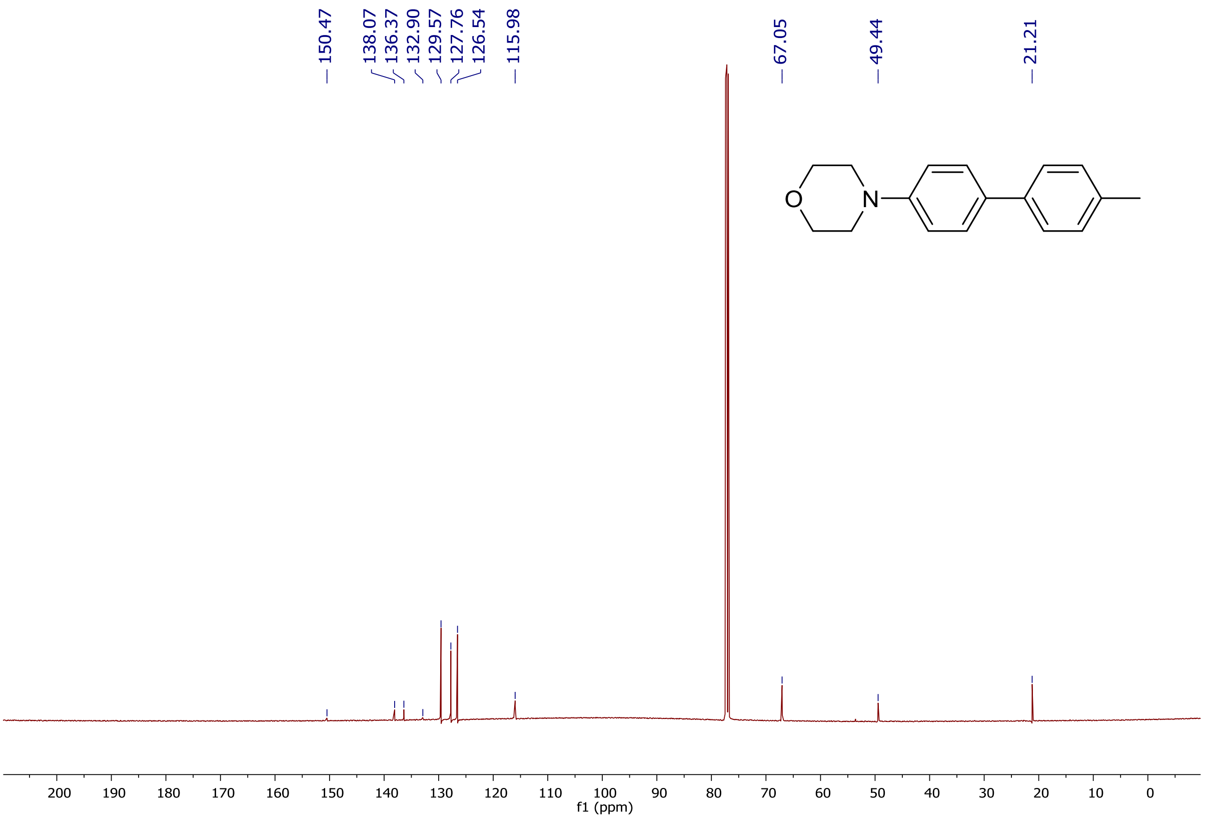

^{1}H NMR of 4-Fluoro-4'-methoxy-1,1'-biphenyl (Table 2, Entry 20)

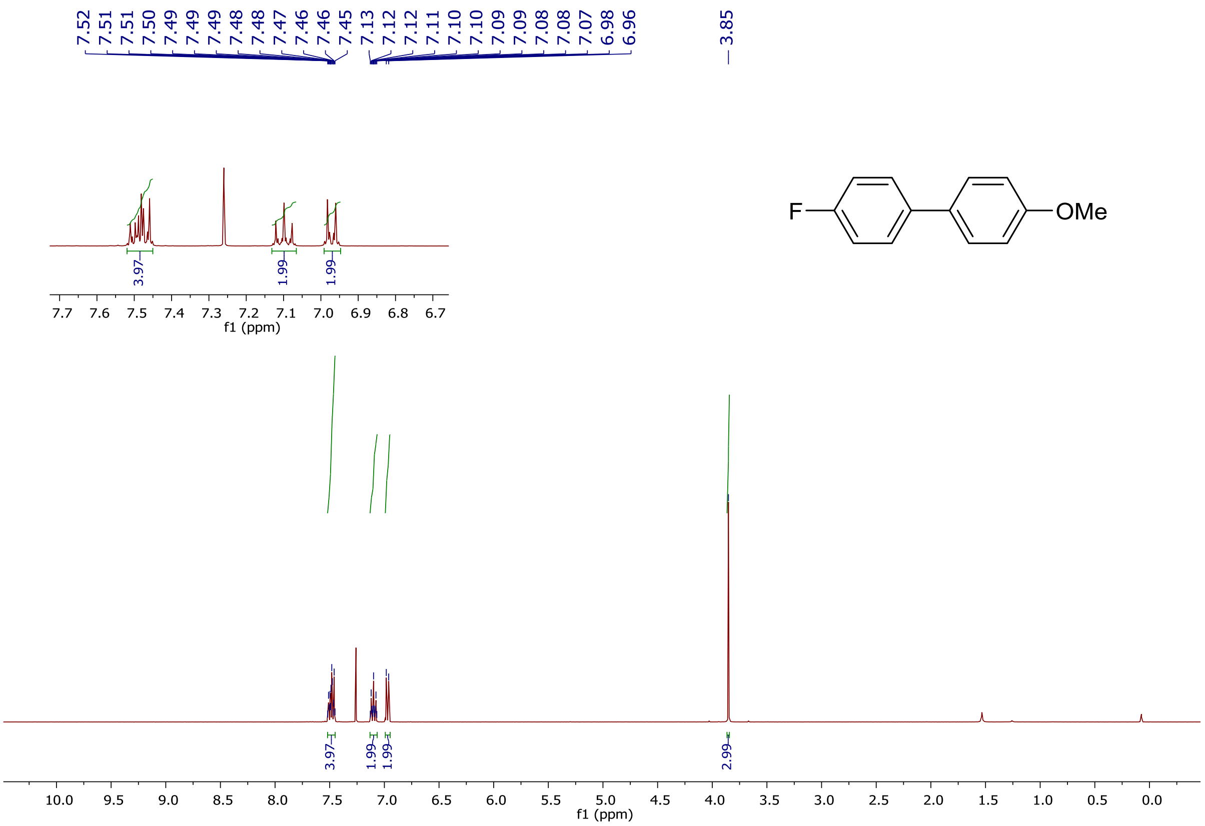

^{13}C NMR of 4-Fluoro-4'-methoxy-1,1'-biphenyl (Table 2, Entry 20)

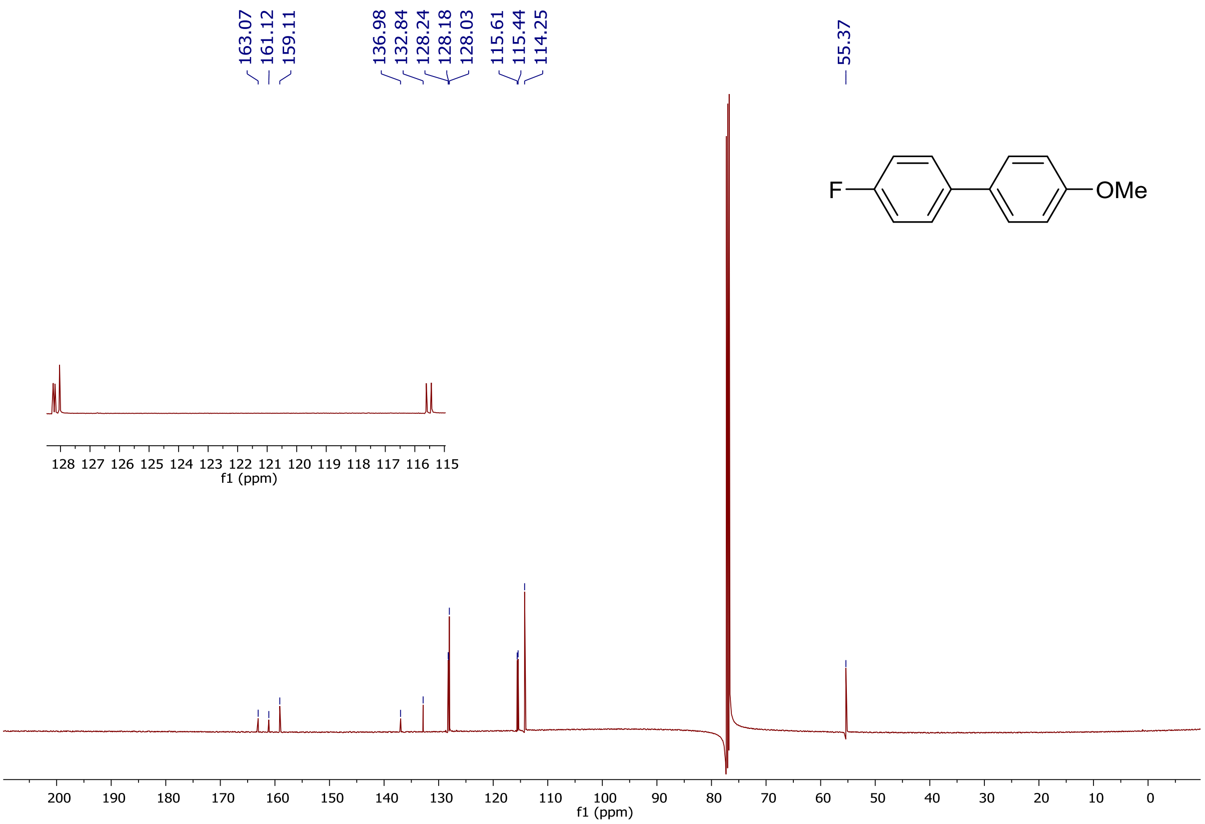

^{1}H NMR of 2,4'-Dimethoxy-1,1'-biphenyl (Table 2, Entry 21)

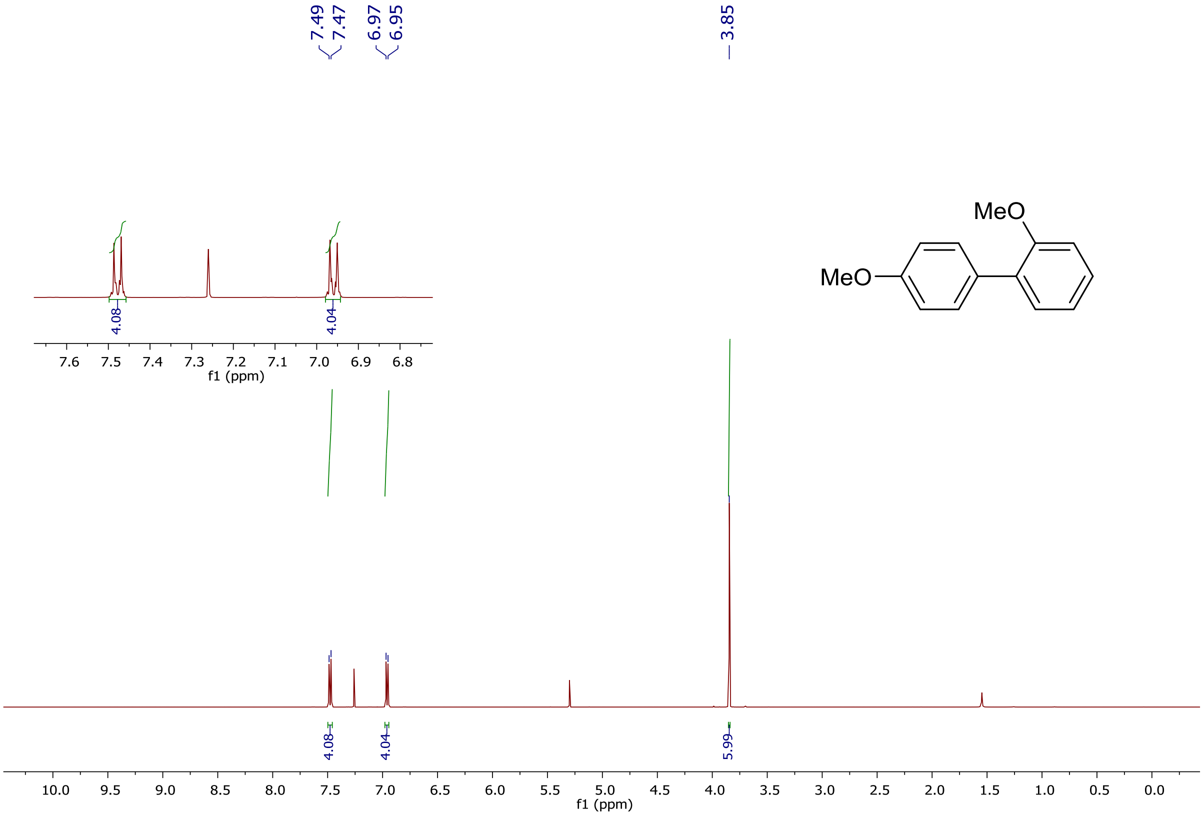

^{13}C NMR of 2,4'-Dimethoxy-1,1'-biphenyl (Table 2, Entry 21)

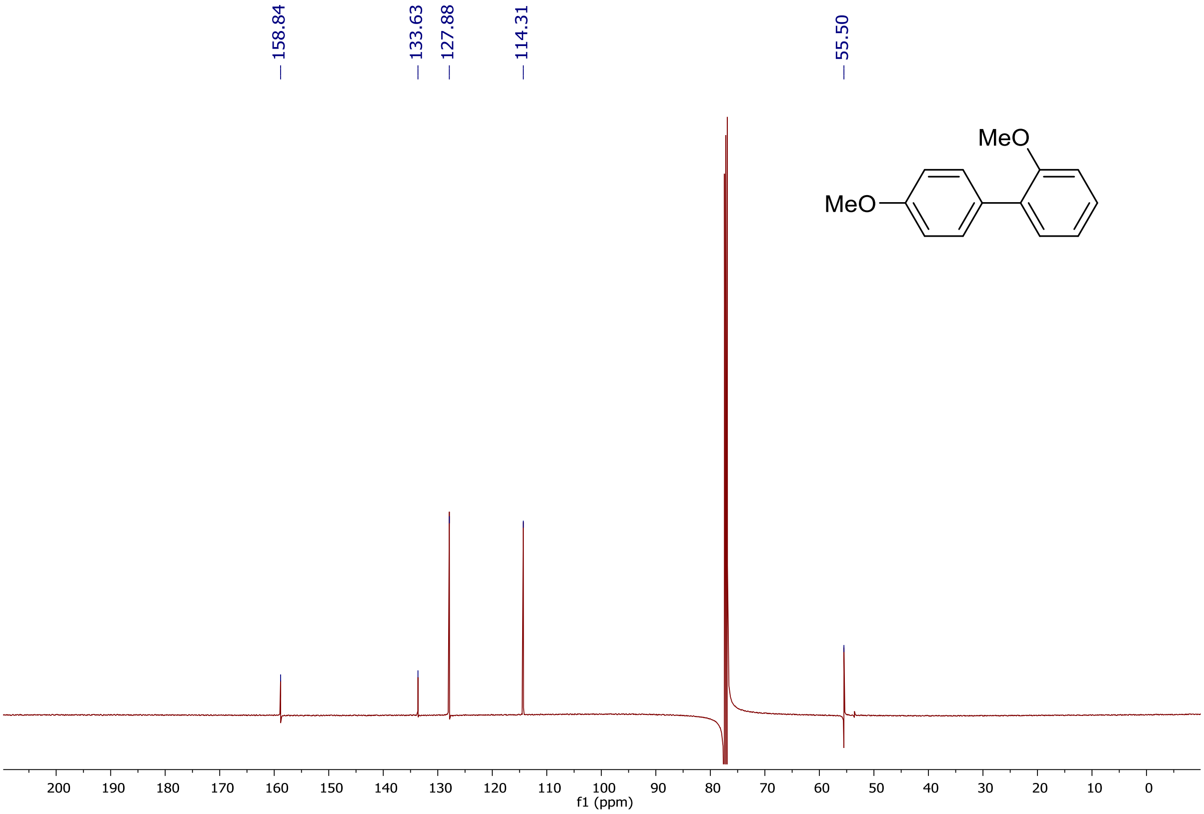

^{1}H NMR of 4-(Benzyloxy)-1,1'-biphenyl (Table 2, Entry 22)

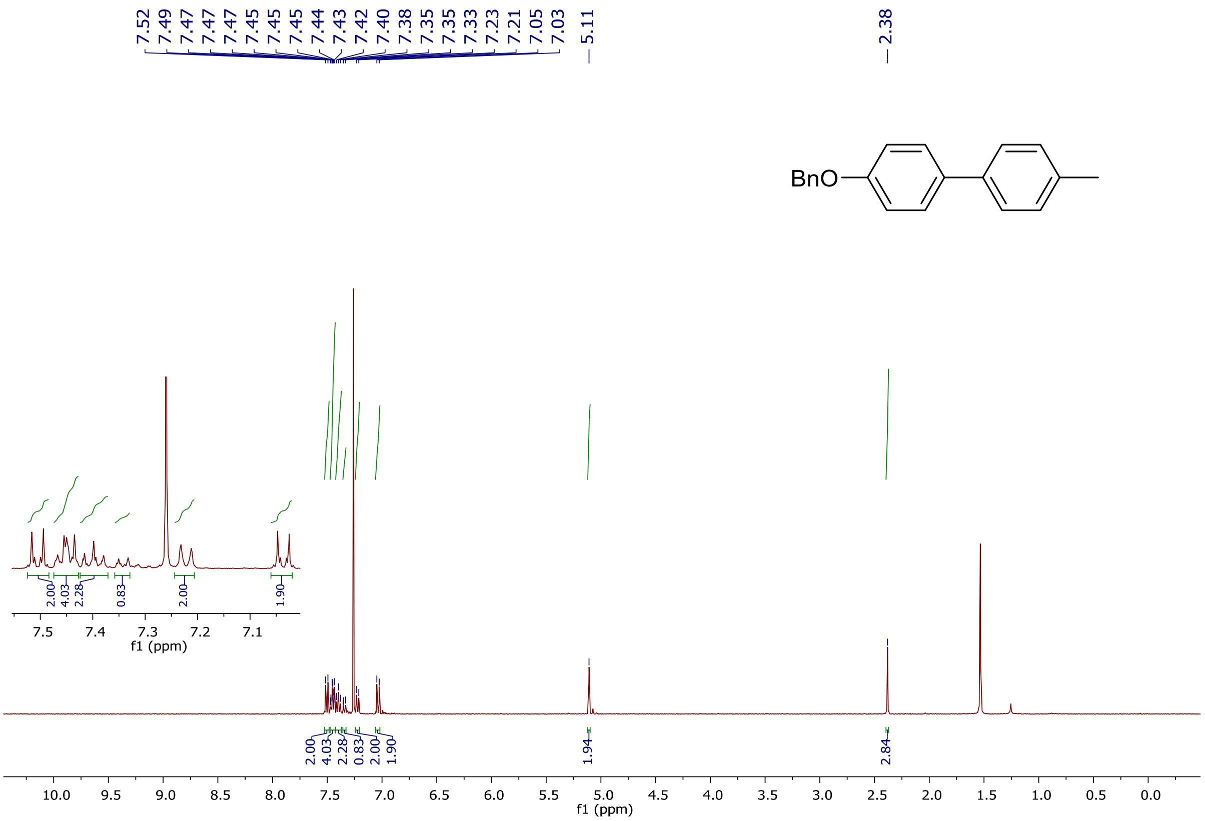

^{13}C NMR of 4-(Benzyloxy)-1,1'-biphenyl (Table 2, Entry 22)

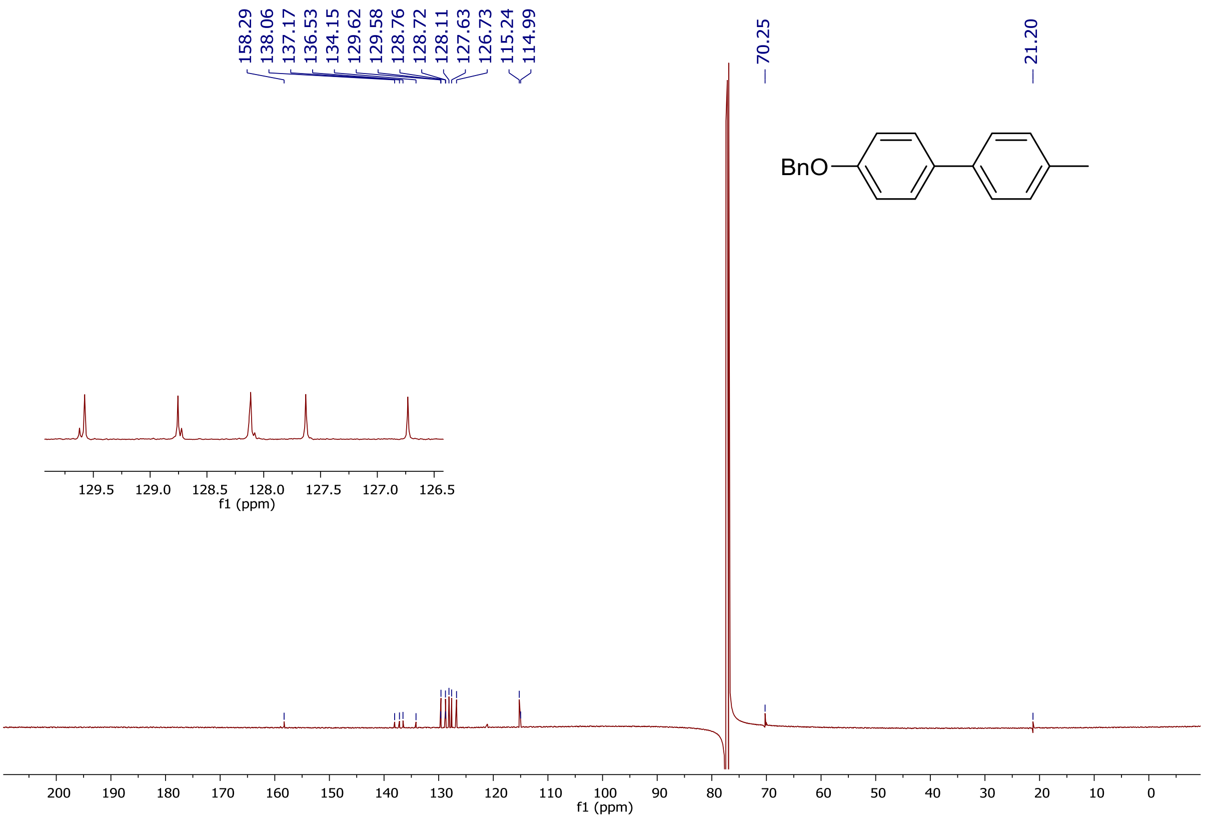

^{1}H NMR of 3,5-Dimethoxy-4'-(trifluoromethyl)-1,1'-biphenyl (Table 2, Entry 23)

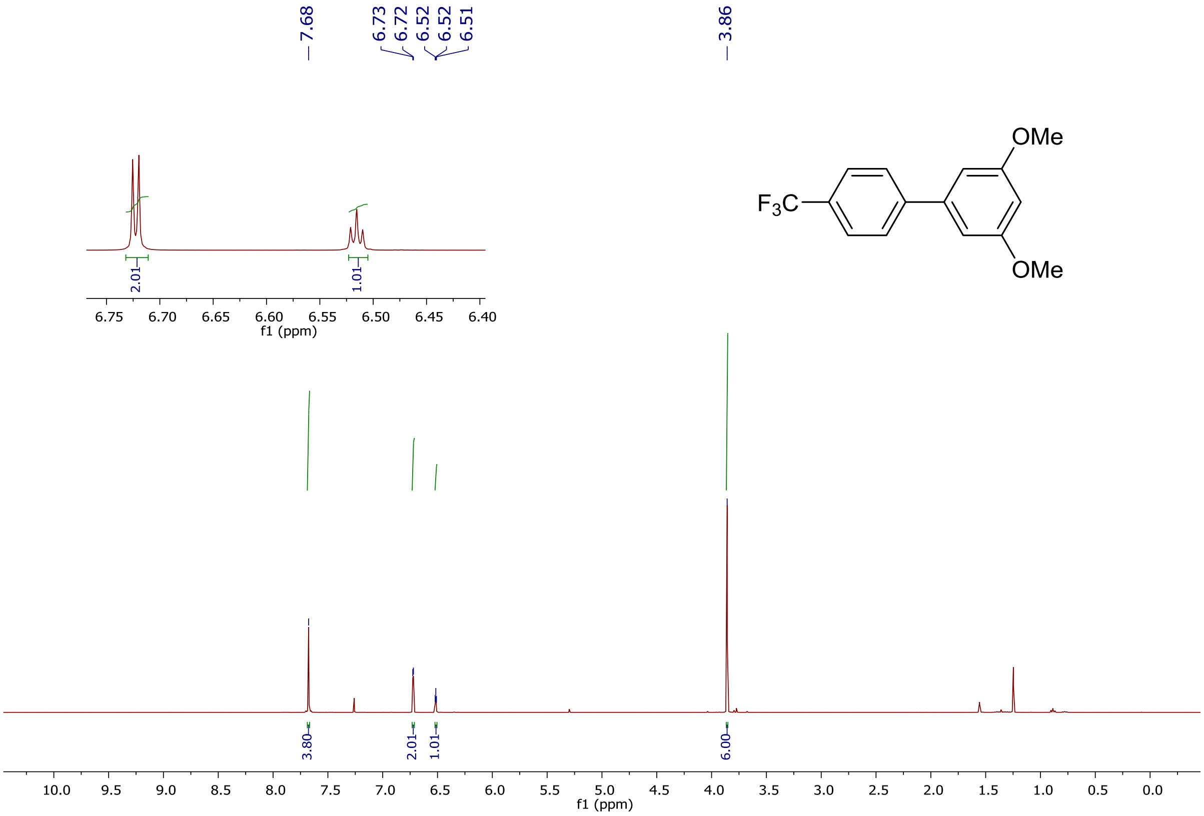

^{13}C NMR of 3,5-Dimethoxy-4'-(trifluoromethyl)-1,1'-biphenyl (Table 2, Entry 23)

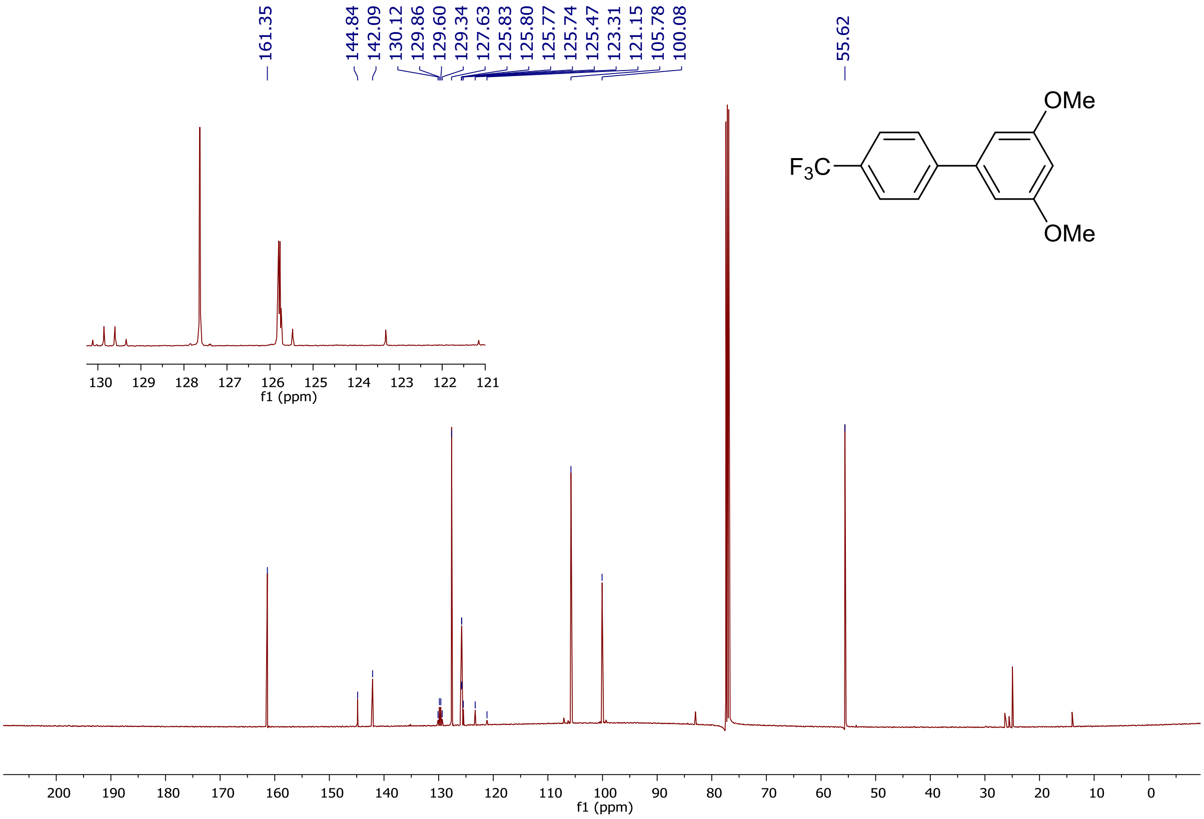

^{1}H NMR of 4-(Trifluoromethoxy)-1,1'-biphenyl (Table 2, Entry 24)

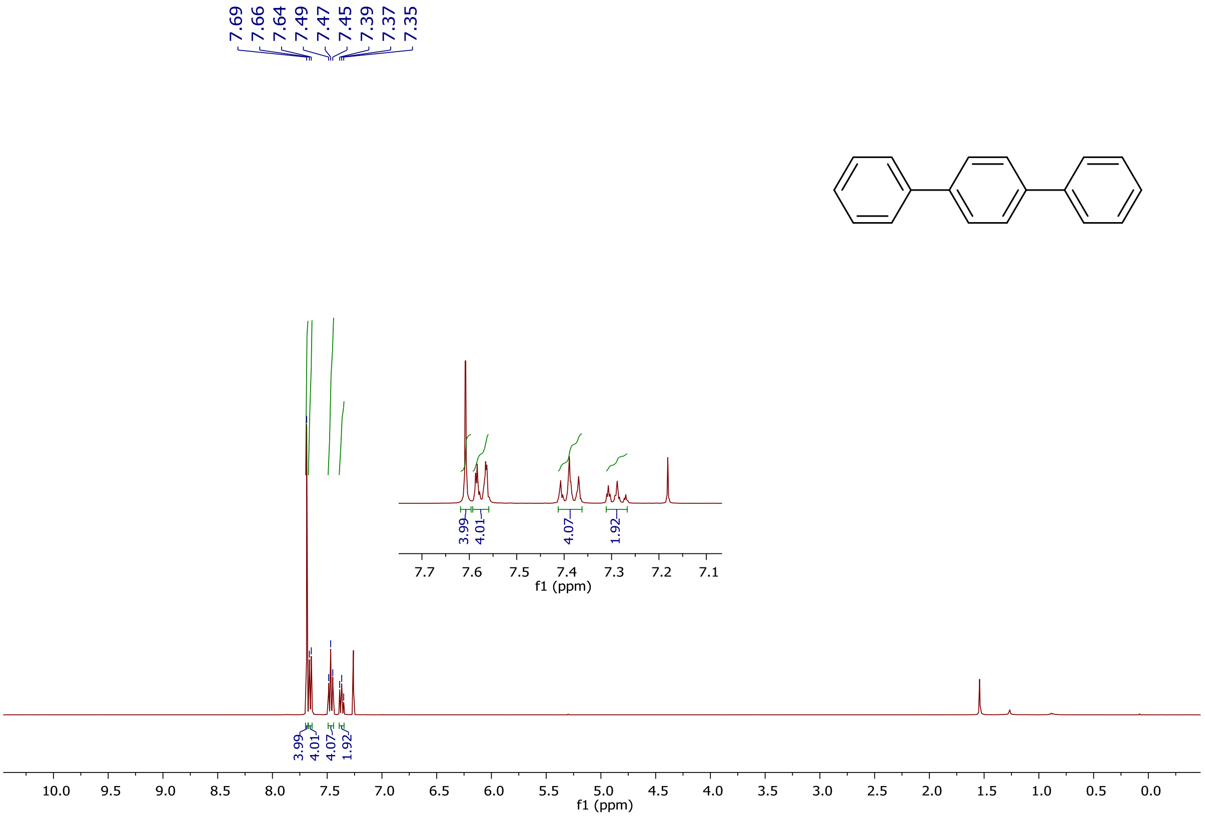

^{13}C NMR of 4-(Trifluoromethoxy)-1,1'-biphenyl (Table 2, Entry 24)

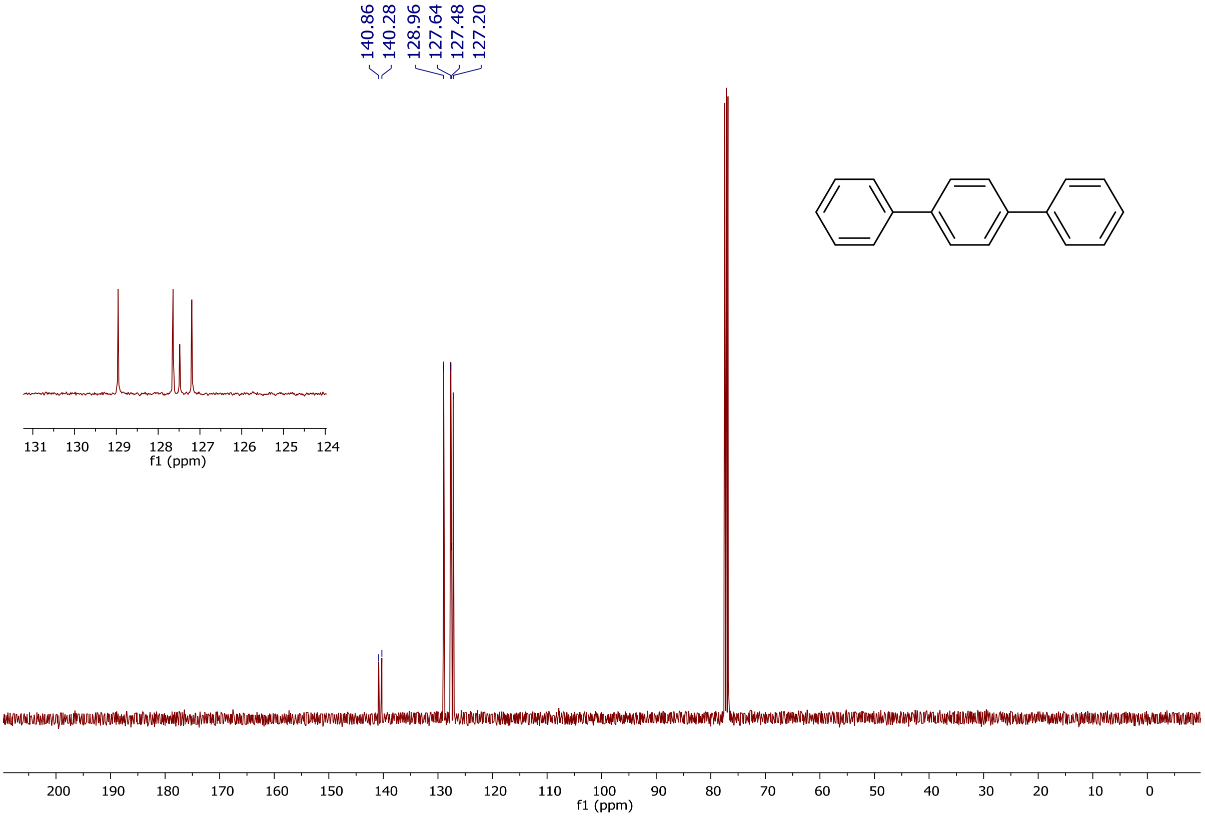

^{1}H NMR of 3-Phenylquinoline (Table 2, Entry 25)

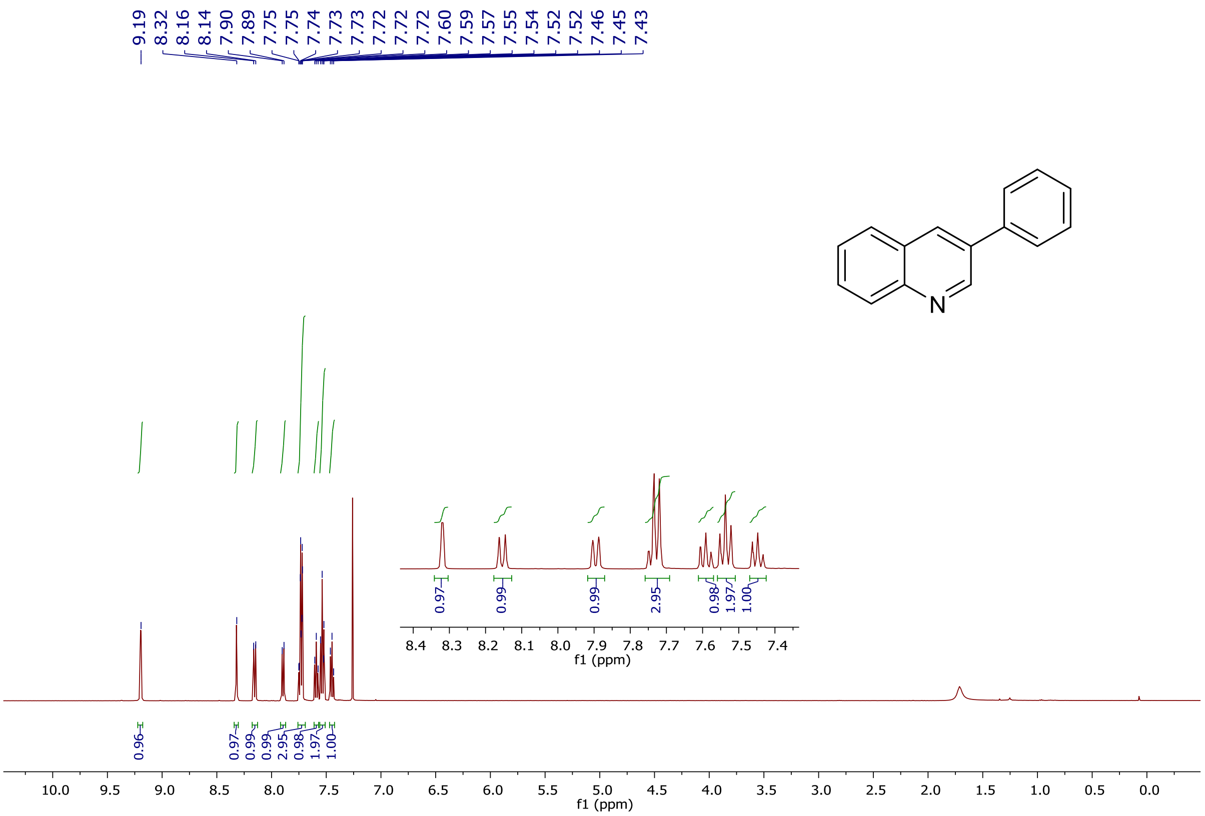

^{13}C NMR of 3-Phenylquinoline (Table 2, Entry 25)

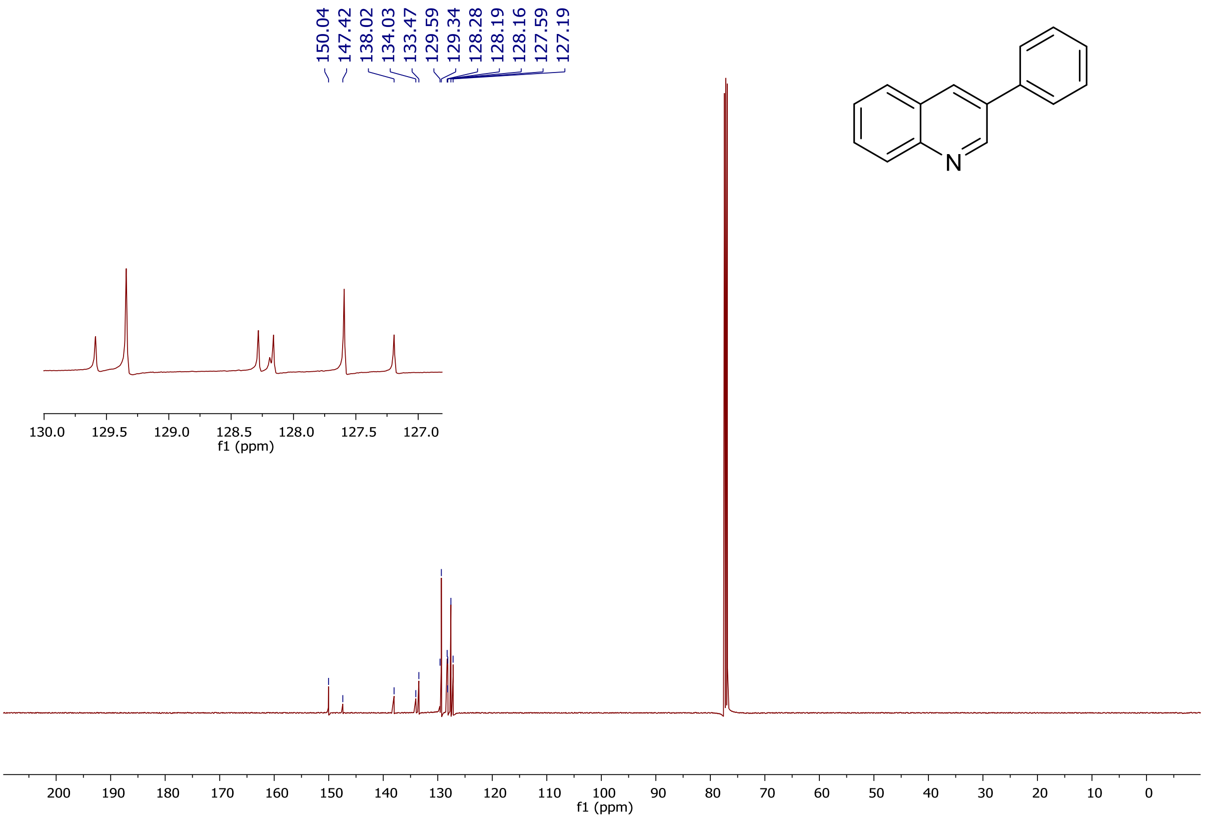

^{1}H NMR of 8-Phenylquinoline (Table 2, Entry 26)

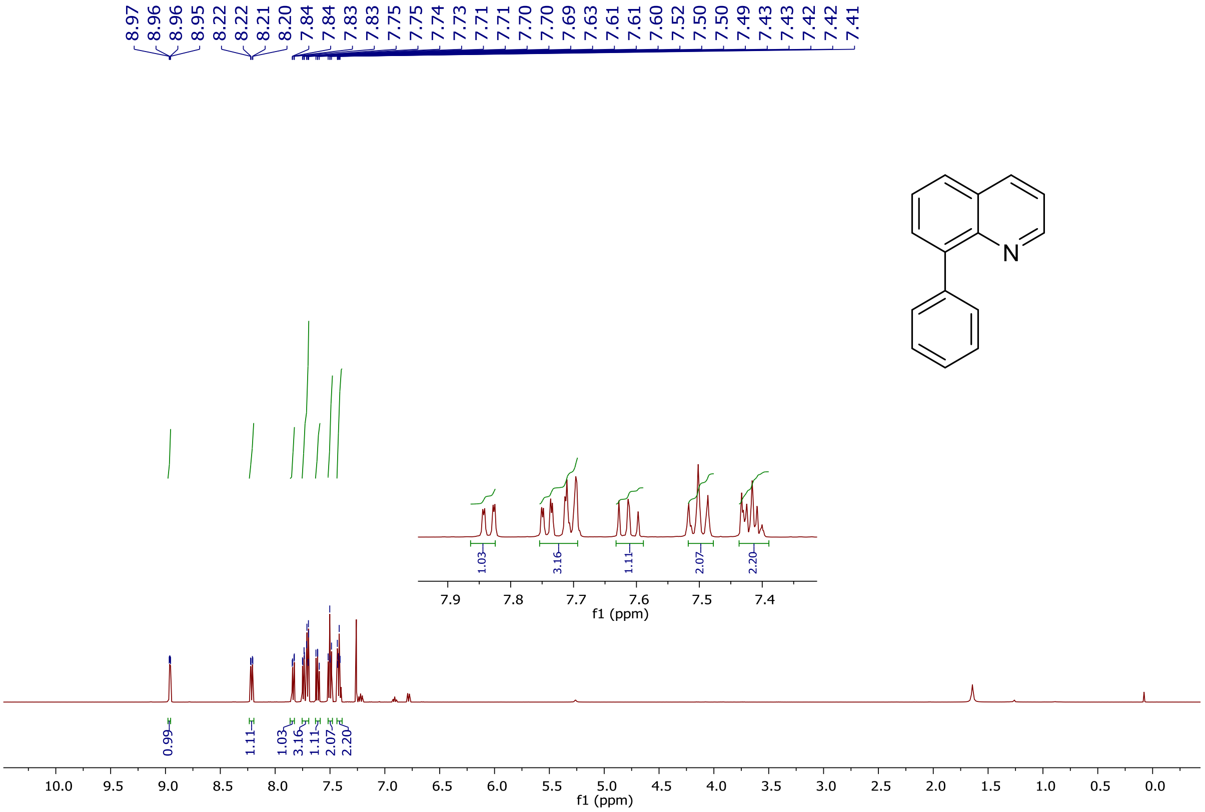

^{13}C NMR of 8-Phenylquinoline (Table 2, Entry 26)

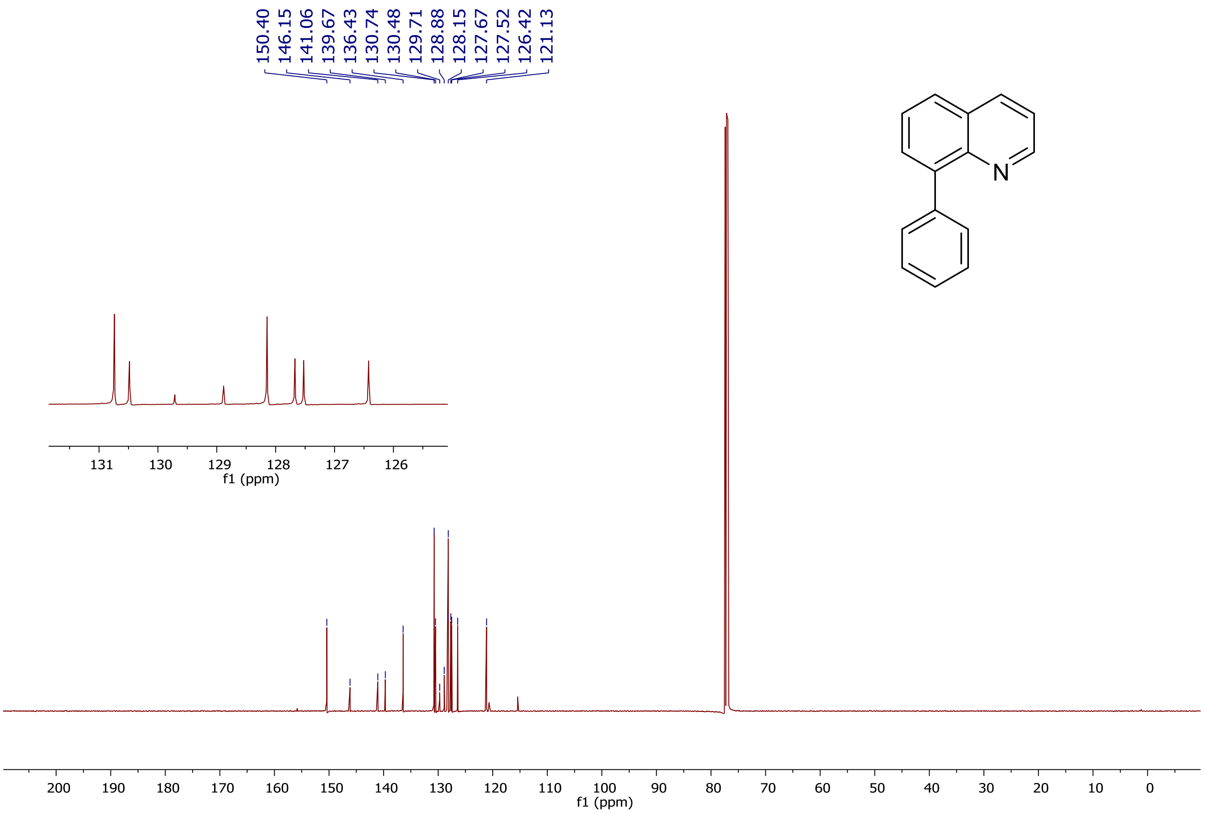

^{1}H NMR of 3-Phenylbenzo[b]thiophene (Table 2, Entry 27)

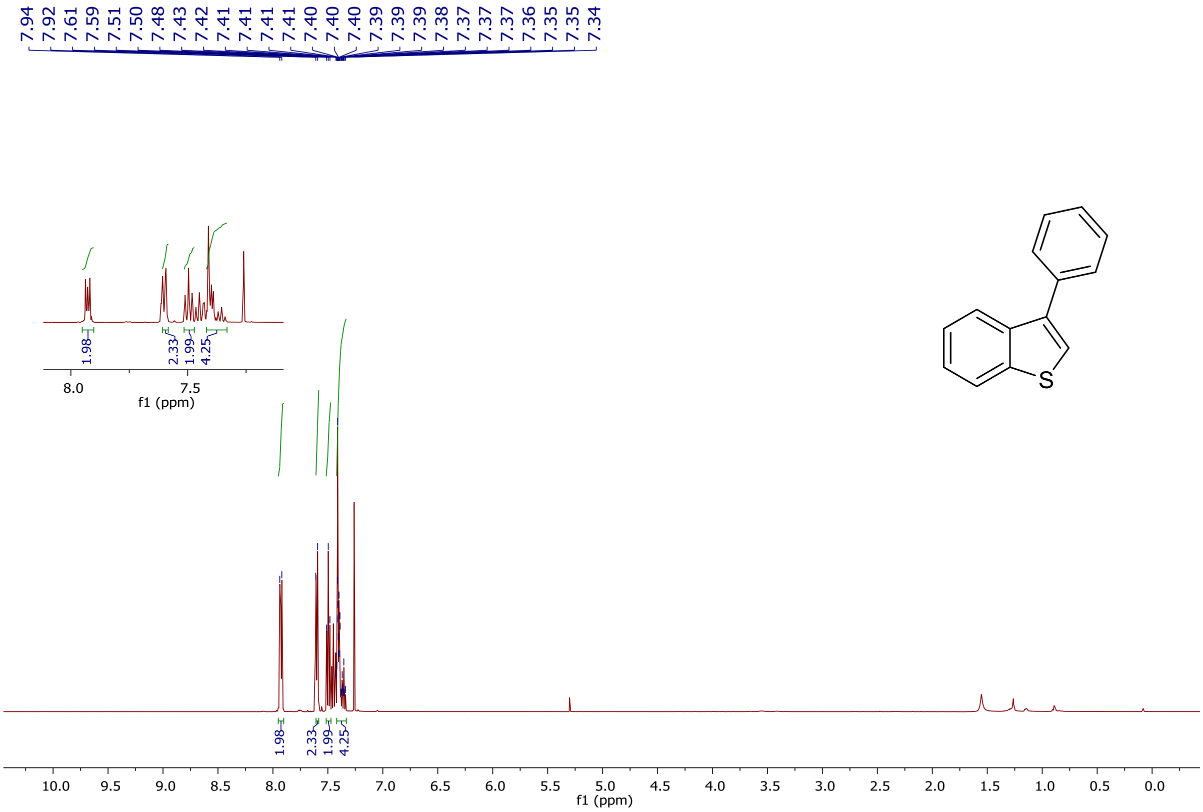

^{13}C NMR of 3-Phenylbenzo[b]thiophene (Table 2, Entry 27)

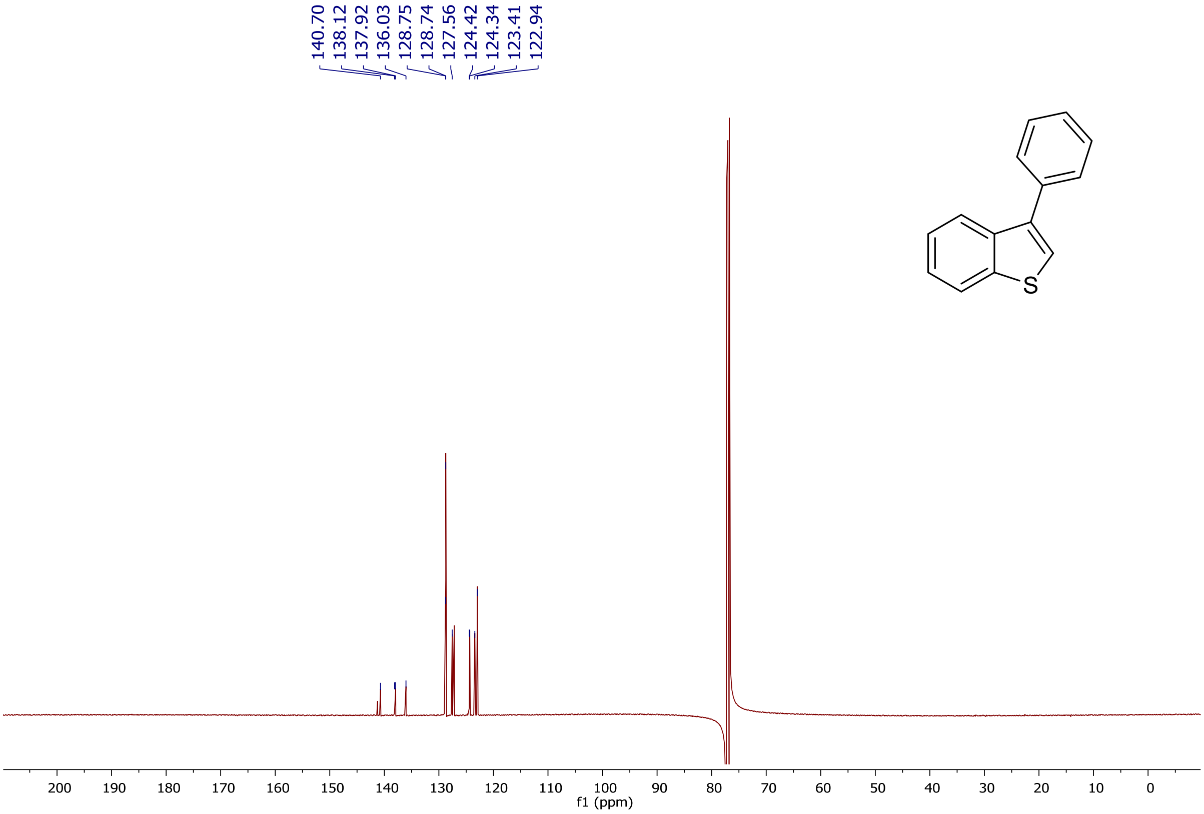

^{1}H NMR of 2-([1,1'-Biphenyl]-2-yl)-5-methyl-2,4-dihydro-3H-pyrazol-3-one (5a)

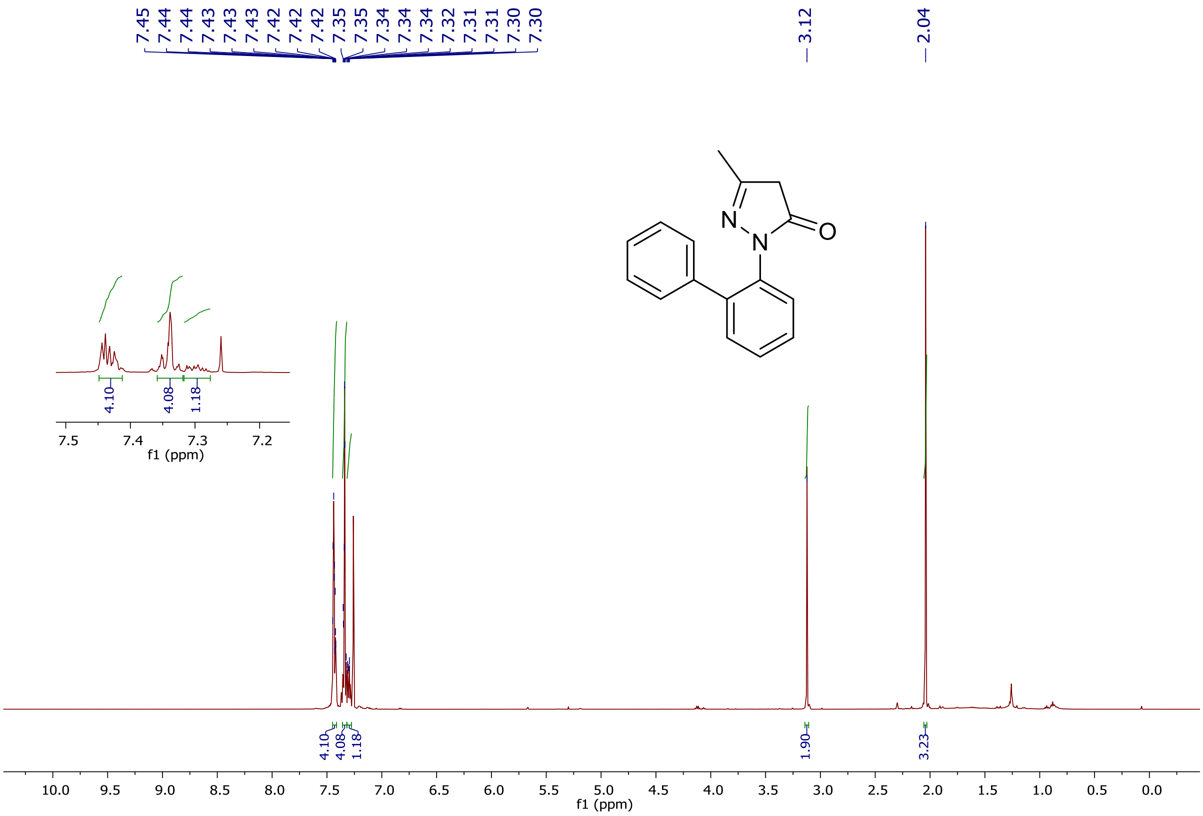

^{13}C NMR of 2-([1,1'-Biphenyl]-2-yl)-5-methyl-2,4-dihydro-3H-pyrazol-3-one (5a)

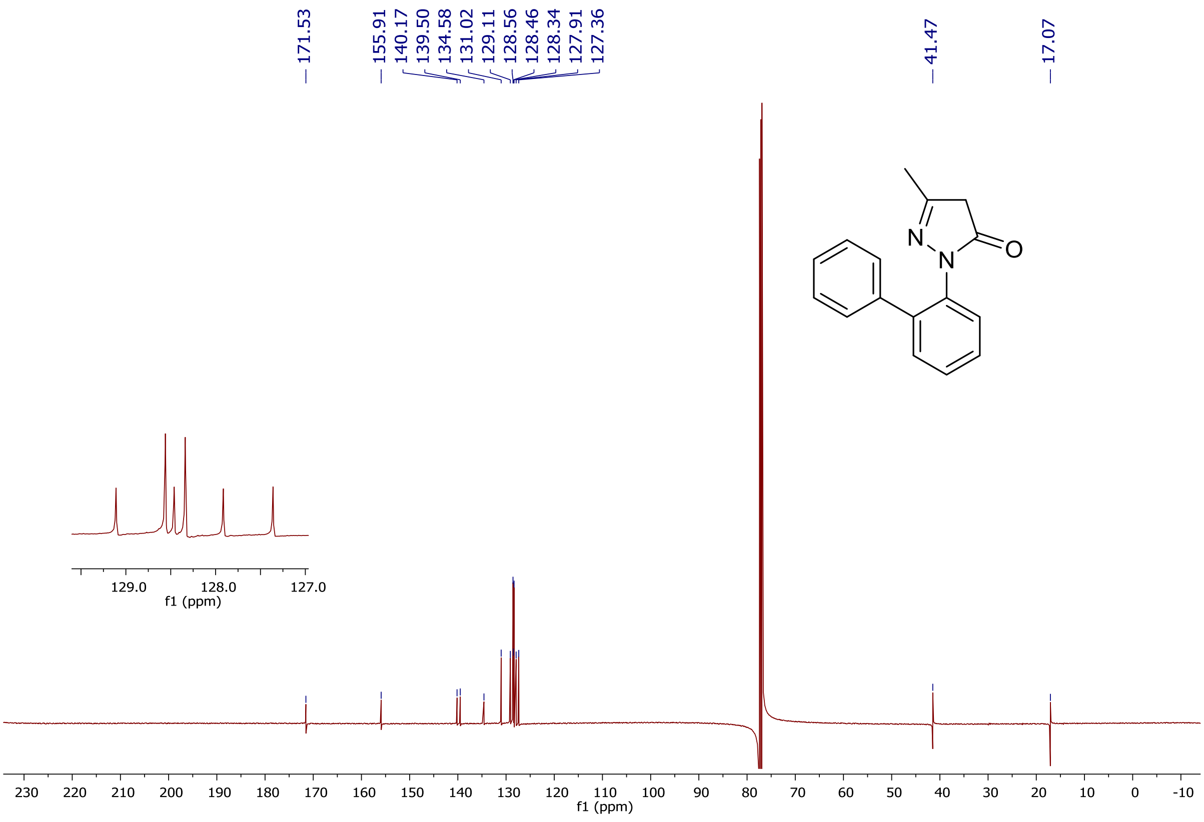

^{1}H NMR of 5-Methyl-2-(4'-methyl-[1,1'-biphenyl]-2-yl)-2,4-dihydro-3H-pyrazol-3-one (5b)

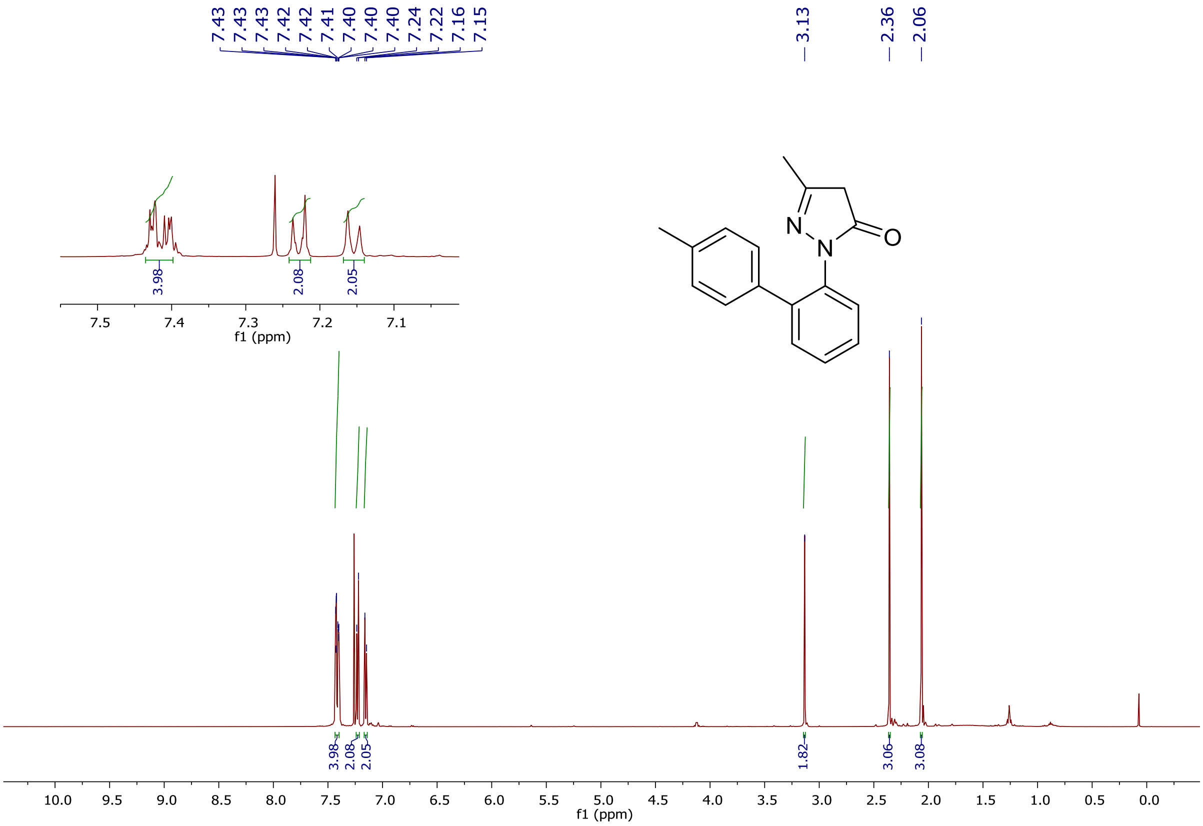

^{13}C NMR of 5-Methyl-2-(4'-methyl-[1,1'-biphenyl]-2-yl)-2,4-dihydro-3H-pyrazol-3-one (5b)

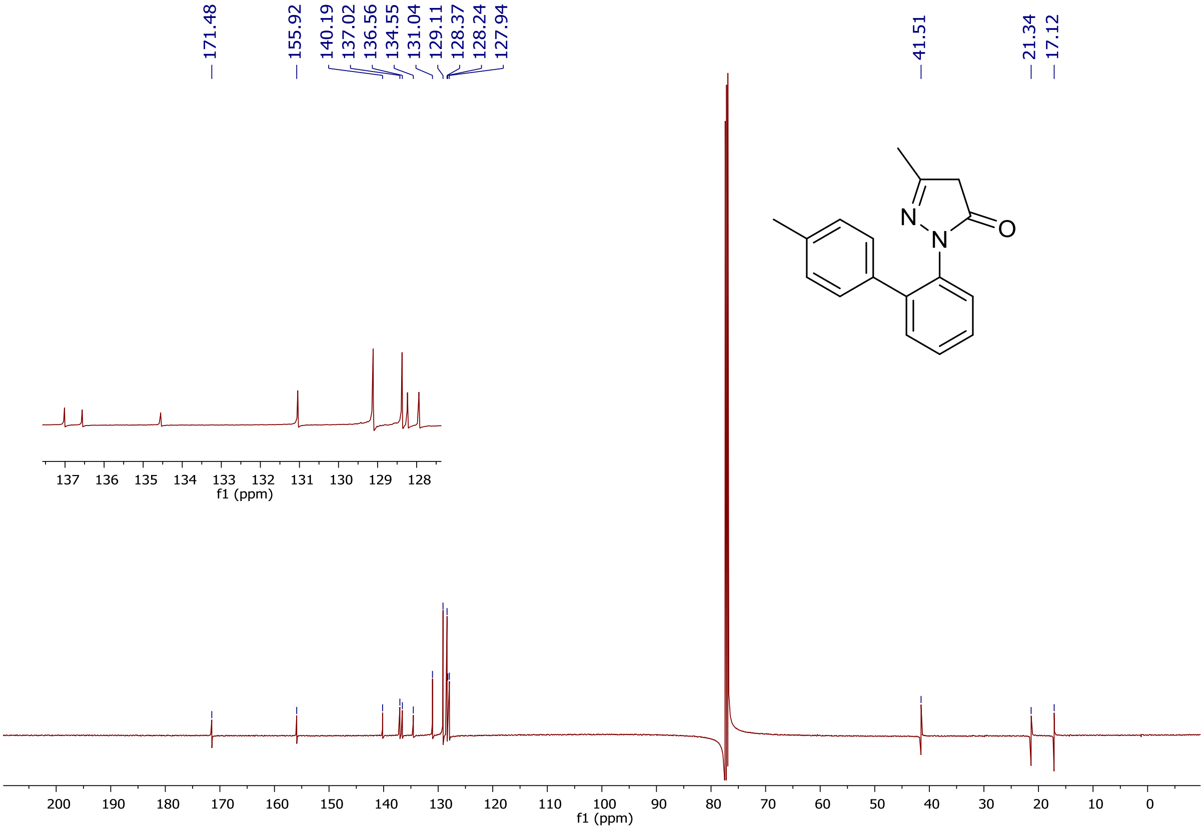

^{1}H NMR of 2-(4'-Methoxy-[1,1'-biphenyl]-2-yl)-5-methyl-2,4-dihydro-3H-pyrazol-3-one (5c)

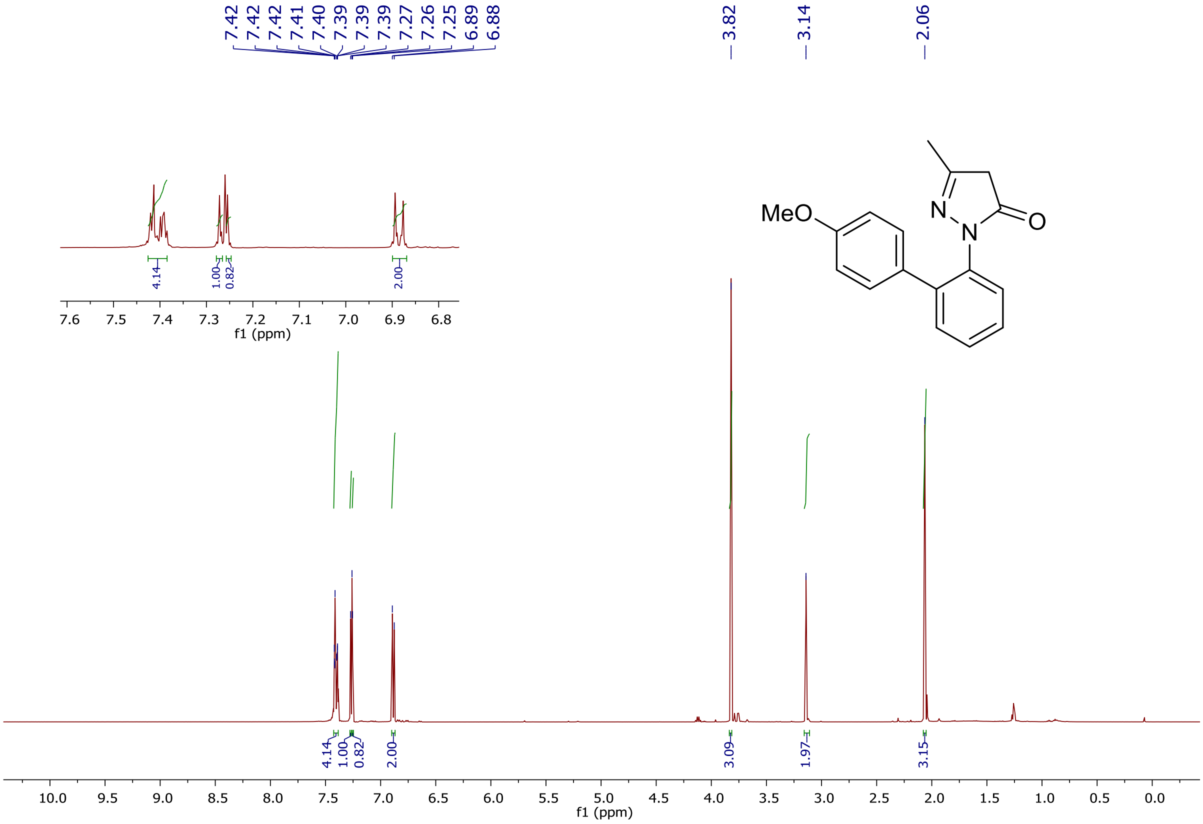

^{13}C NMR of 2-(4'-Methoxy-[1,1'-biphenyl]-2-yl)-5-methyl-2,4-dihydro-3H-pyrazol-3-one (5c)

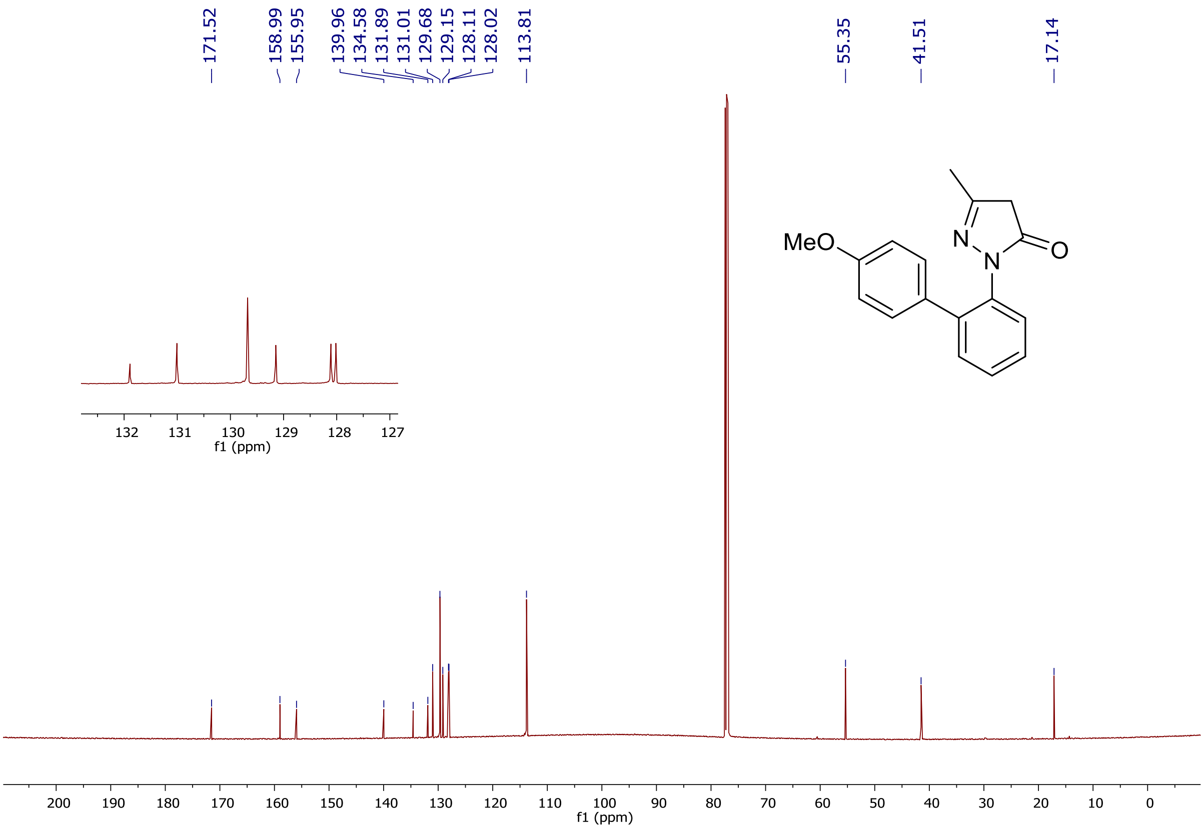

^{1}H NMR of 2-(4'-(Benzyloxy)-[1,1'-biphenyl]-2-yl)-5-methyl-2,4-dihydro-3H-pyrazol-3-one (5d)

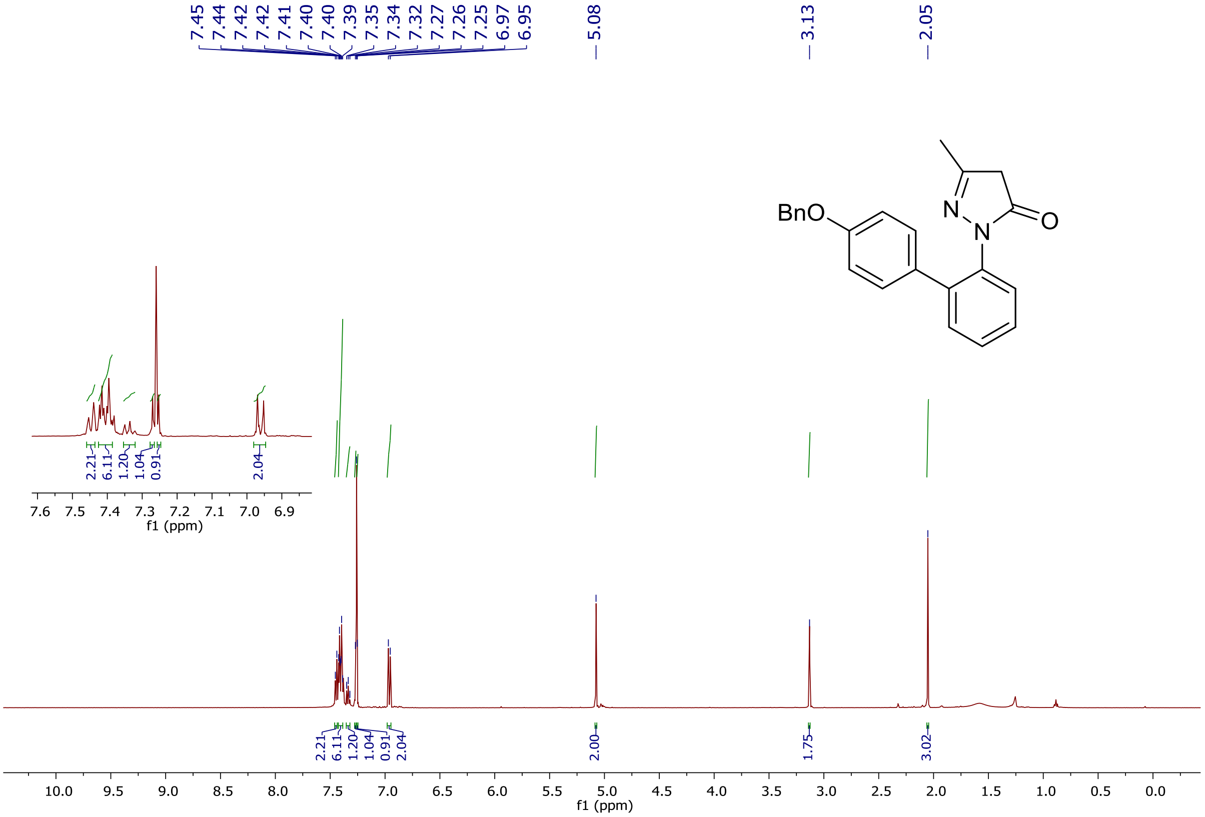

^{13}C NMR of 2-(4'-(Benzyloxy)-[1,1'-biphenyl]-2-yl)-5-methyl-2,4-dihydro-3H-pyrazol-3-one (5d)

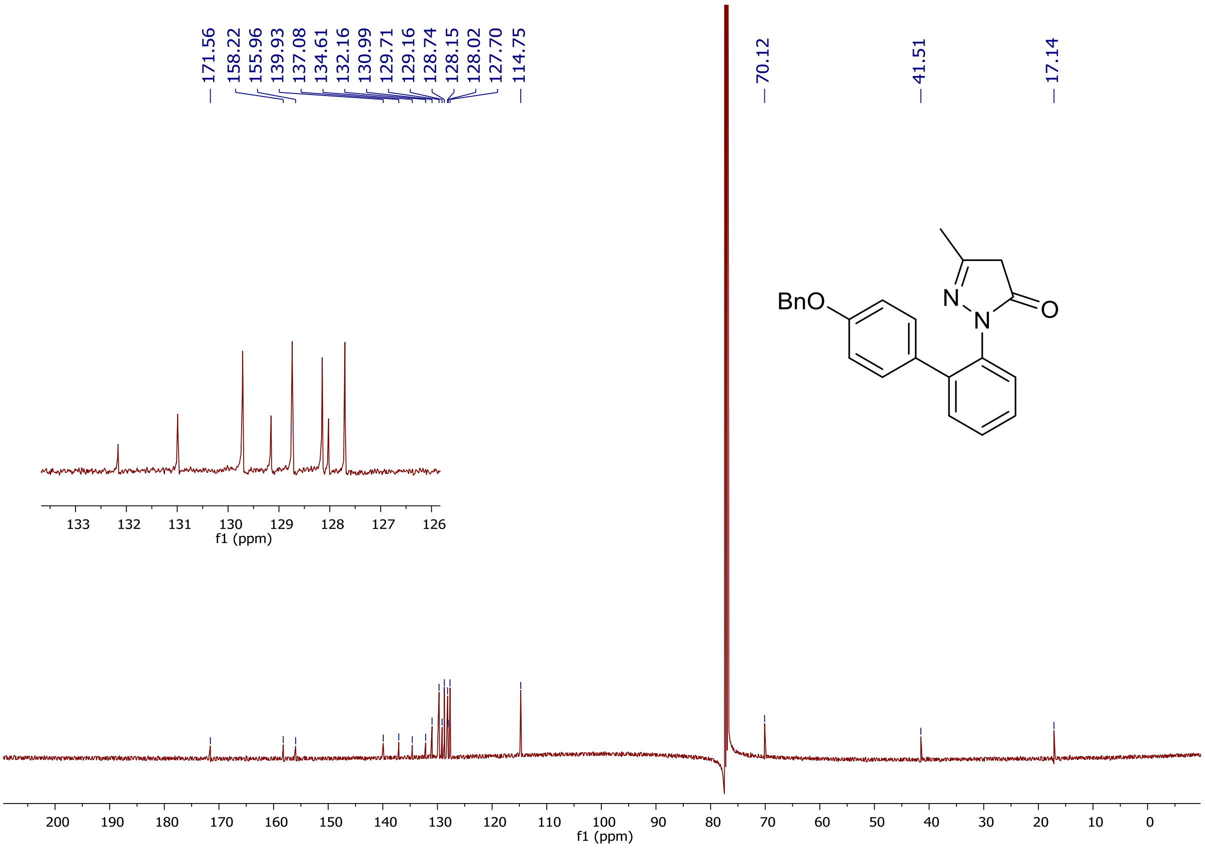

^{1}H NMR of 5-Methyl-2-(4'-(trifluoromethyl)-[1,1'-biphenyl]-2-yl)-2,4-dihydro-3H-pyrazol-3-one(5e)

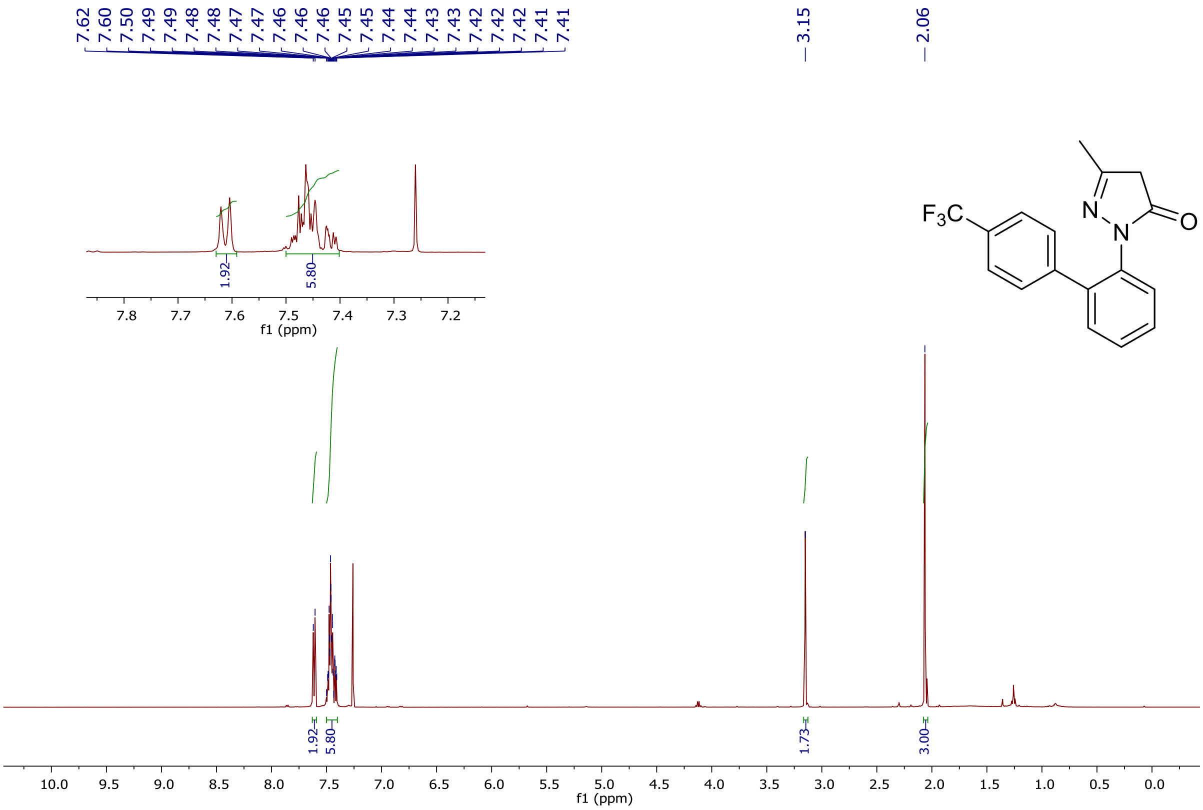

^{13}C NMR of 5-Methyl-2-(4'-(trifluoromethyl)-[1,1'-biphenyl]-2-yl)-2,4-dihydro-3H-pyrazol-3-one (5e)

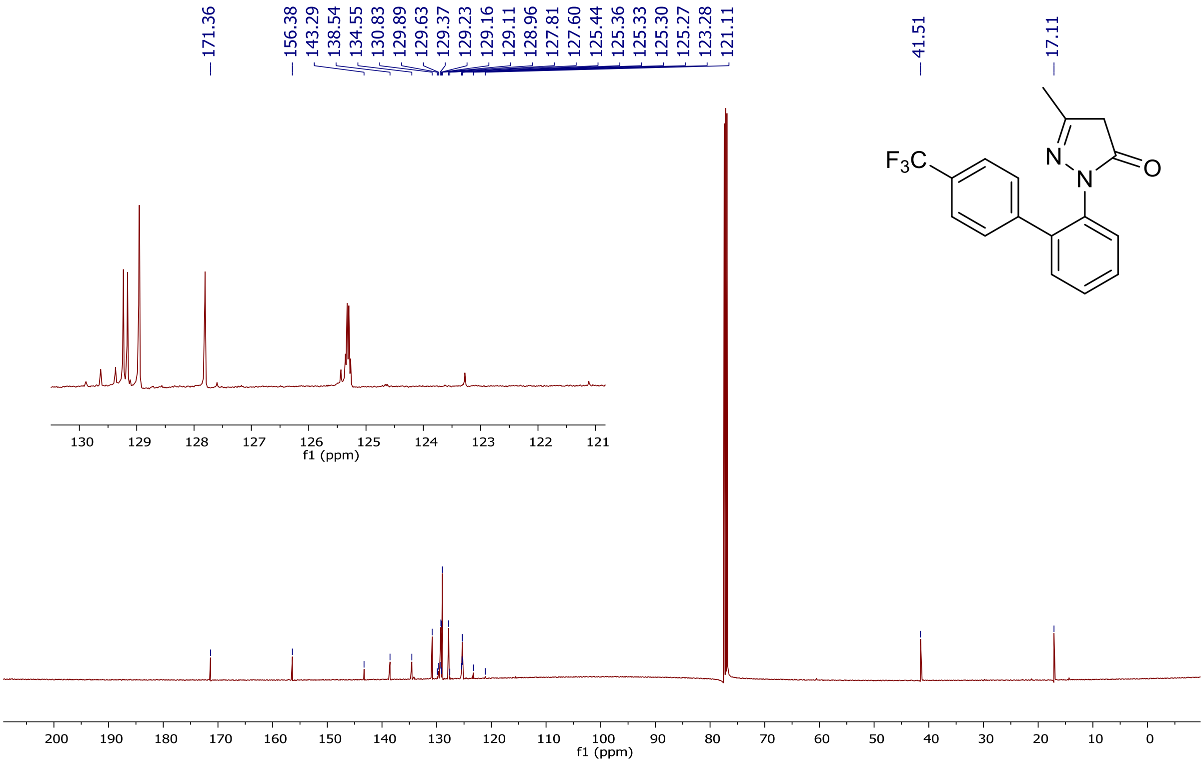

^{1}H NMR of 2-(4'-Fluoro-[1,1'-biphenyl]-2-yl)-5-methyl-2,4-dihydro-3H-pyrazol-3-one (5f)

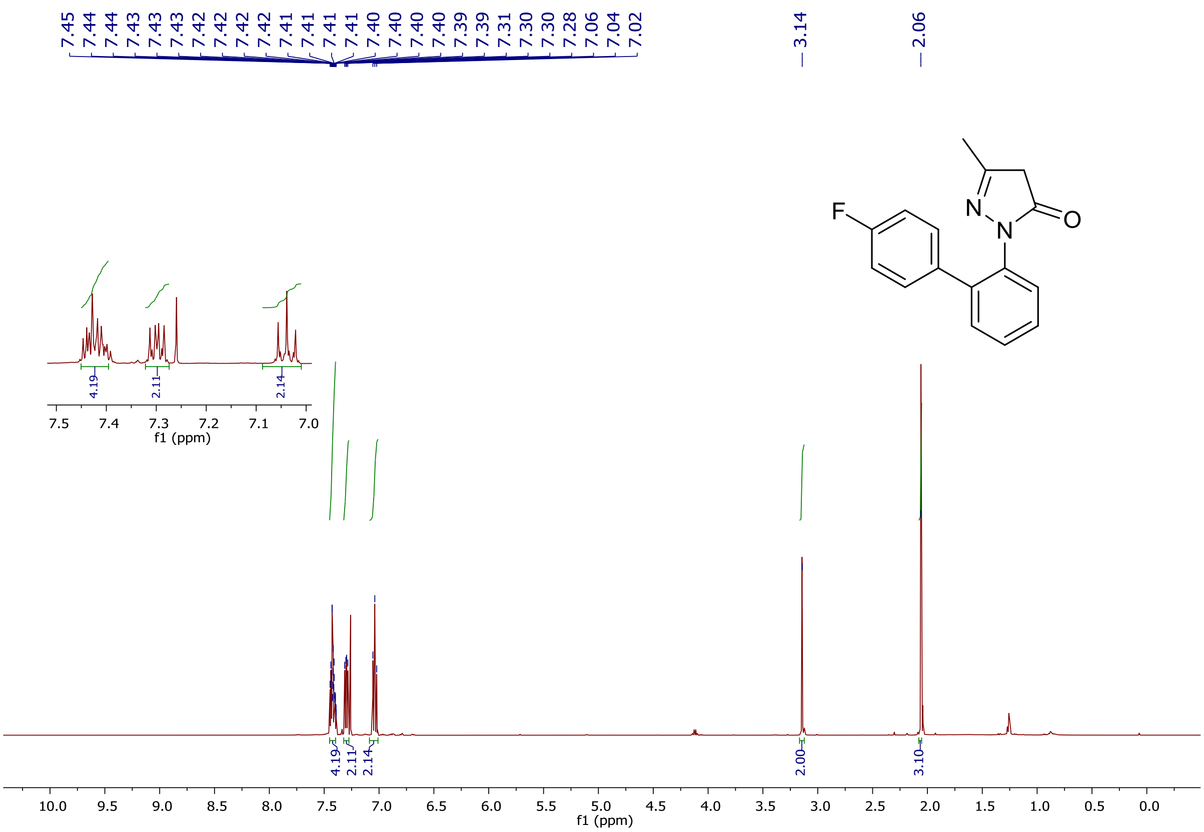

^{13}C NMR of 2-(4'-Fluoro-[1,1'-biphenyl]-2-yl)-5-methyl-2,4-dihydro-3H-pyrazol-3-one (5f)

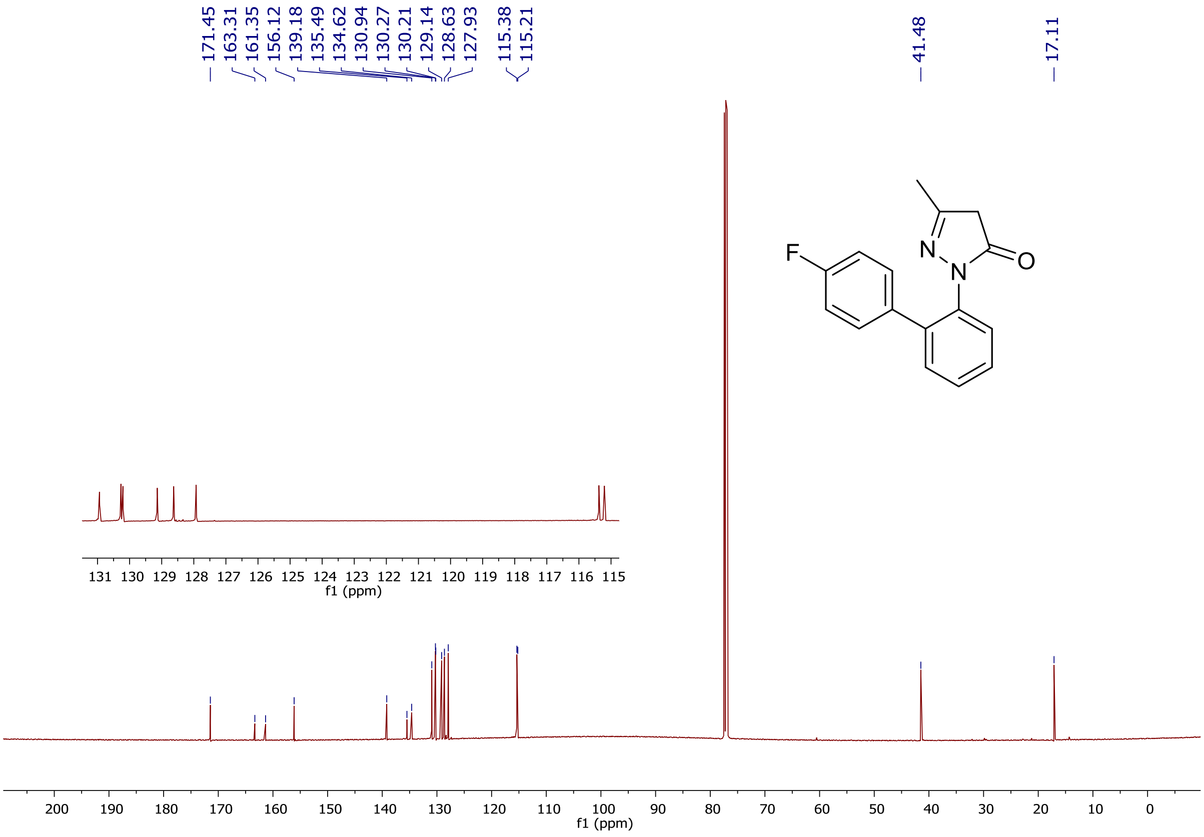

^{1}H NMR of 5-Methyl-2-(4'-morpholino-[1,1'-biphenyl]-2-yl)-2,4-dihydro-3H-pyrazol-3-one (5g)

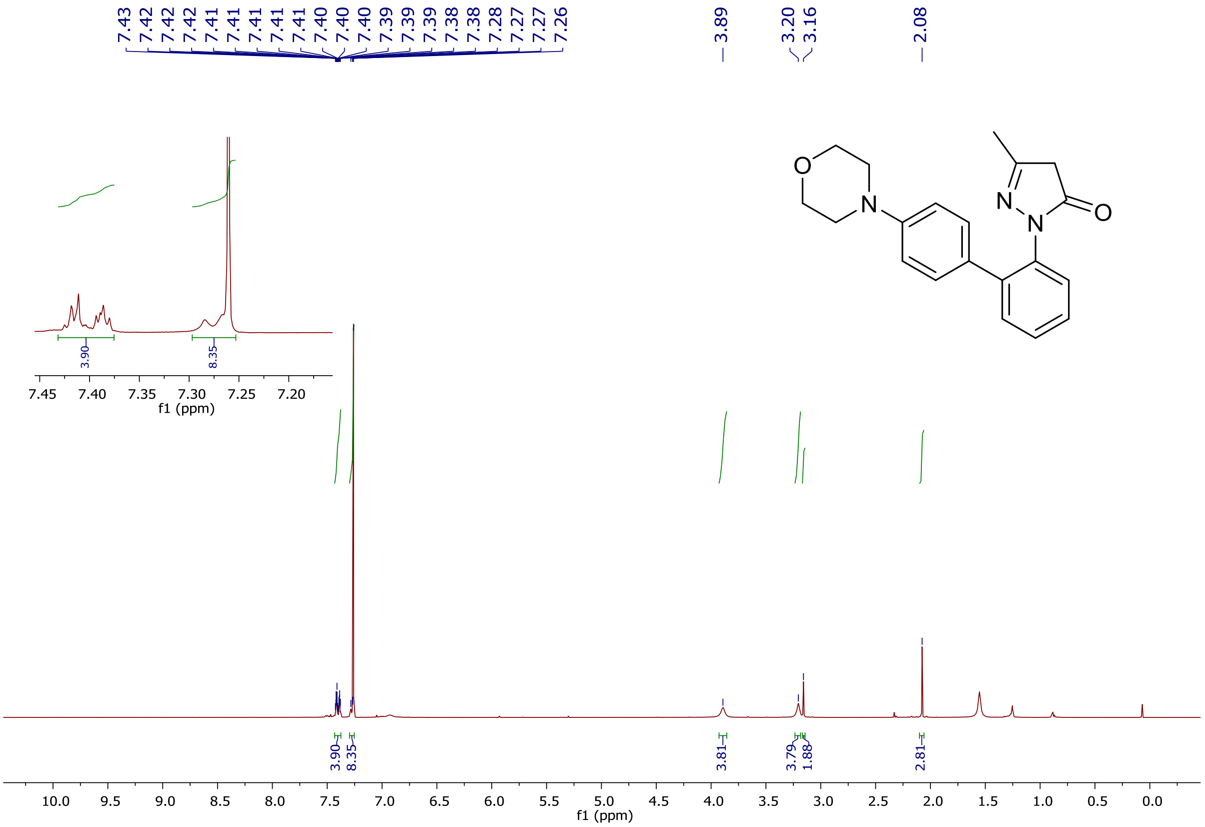

^{13}C NMR of 5-Methyl-2-(4'-morpholino-[1,1'-biphenyl]-2-yl)-2,4-dihydro-3H-pyrazol-3-one (5g)

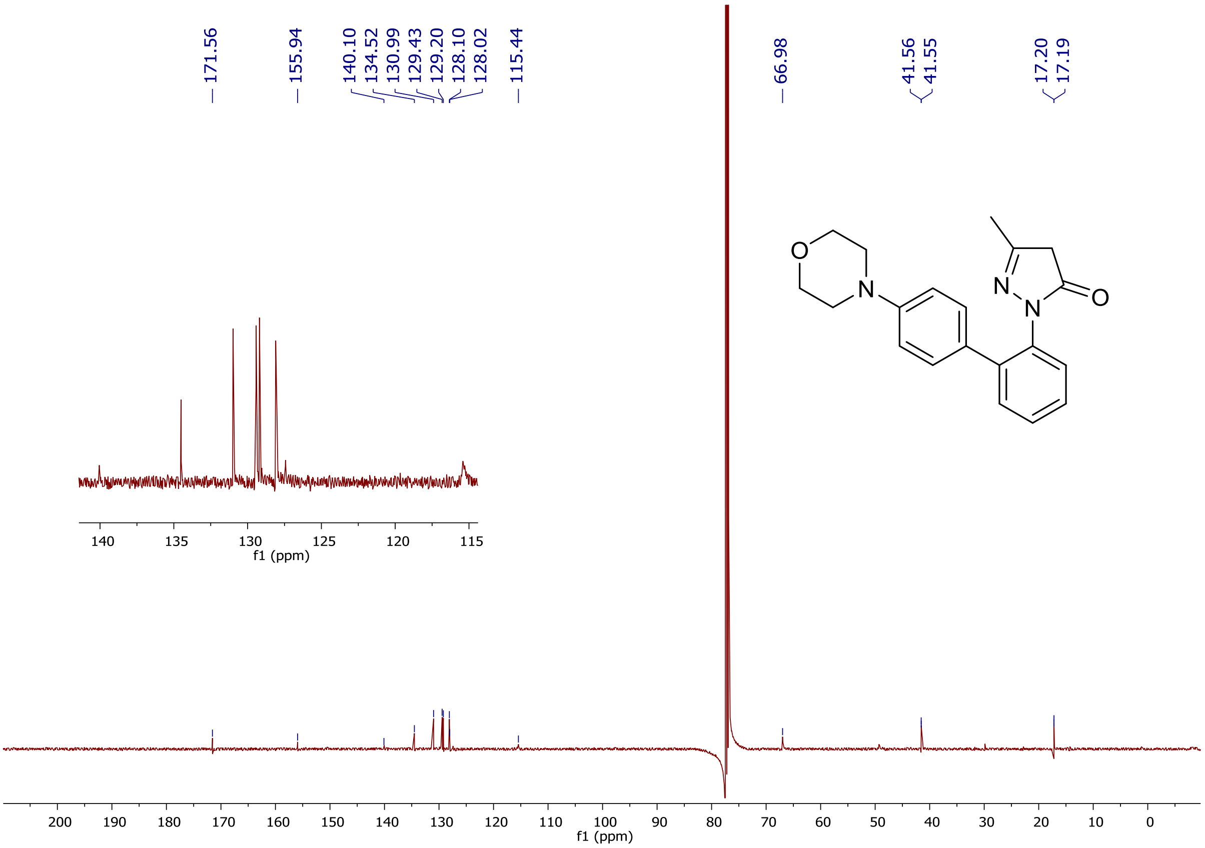

3. Crystallographic Data

X-ray diffraction experiments of complexes **6a** and **6b** were conducted at 100(2) K on a Bruker APEX-II diffractometer using Mo-K_α radiation (λ = 0.71073 Å). Data collections were performed using a CCD area detector from a single crystal mounted on a glass fibre. Intensities were integrated in SAINT [18] and absorption corrections were based on equivalent reflections were carried out using SADABS [19]. Using Olex2 [20], the structures were solved with ShelXT [21] using Intrinsic Phasing and refined using full matrix least squares against F^2 within ShelXL [22].

The crystal structure **6b** was refined as a racemic twin and the twin fraction is 0.70:0.30. In structure **6b**, the occupancies of the disordered atom C24 and C24a were refined, and restraints and constraints were applied to maintain sensible thermal and geometric parameters.

The crystallographic and refinement details are given in Table S5. The x-ray crystallographic coordinates for structures **6a** and **6b** have been deposited at the Cambridge Crystallographic Data Centre (CCDC) under deposition numbers 1575368 and 1585369 respectively. These data are provided free of charge by The Cambridge Crystallographic Data Centre.

Table S5: Crystallographic data and summary of data collection and refinement for **6a** and **6b**

	**[Co(SIPr)(dvtms)], 6a**	**[Co(SIPr)(norbornene)$_2$], 6b**
**Empirical formula**	$C_{35}H_{56}CoN_2OSi_2$	$C_{41}H_{58}CoN_2$
**Formula weight**	635.92	637.82
**Temperature/K**	100(2)	100(2)
**Crystal system**	triclinic	monoclinic
**Space group**	P-1	$P2_1$
***a*/Å**	11.8030(4)	10.4979(4)
***b*/Å**	11.8881(4)	12.5991(4)
***c*/Å**	12.7843(4)	13.6019(5)
**α/°**	90.098(2)	90
**β/°**	91.964(2)	101.000(2)
**γ/°**	94.508(2)	90
**Volume/Å^3**	1787.21(10)	1765.99(11)
**Z**	2	2
**ρ_{calc}g/cm^3**	1.182	1.199
**μ/mm^{-1}**	0.575	0.516
**F(000)**	686	690

**Crystal size/mm^3**	0.662 × 0.557 × 0.481	0.444 × 0.241 × 0.166
**Radiation**	MoKα (λ = 0.71073)	MoKα (λ = 0.71073)
**2θ range for data collection/°**	3.188 to 55.958	3.952 to 55.936
**Index ranges**	-15 ≤ h ≤ 15, -15 ≤ k ≤ 15, -16 ≤ l ≤ 16	-13 ≤ h ≤ 13, -16 ≤ k ≤ 16, -16 ≤ l ≤ 17
**Reflections collected**	32958	16233
**R_{int}/R_{sigma}**	0.0297/0.0281	0.0328/0.0575
**Data/restraints/parameters**	8579/0/382	8447/15/411
**Goodness-of-fit on F^2**	1.033	1.02
**Final R indexes [I>=2σ (I)]**	R_1 = 0.0298, wR_2 = 0.0728	R_1 = 0.0407, wR_2 = 0.0900
**Final R indexes [all data]**	R_1 = 0.0370, wR_2 = 0.0761	R_1 = 0.0506, wR_2 = 0.0943
**Largest diff. peak/hole / e Å^{-3}**	0.42/-0.26	0.34/-0.21

4. References

[S1] A. J. Arduengo III, R. Krafczyk, R. Schmutzler, *Tetrahedron*, **1999**, *55*, 14523.

[S2] D. F. Evans, *J. Chem. Soc.*, **1959**, 2003.

[S3] R. B. Bedford, P. B. Brenner, E. Carter, T. W. Carvell, P. M. Cogswell, T. Gallagher, J. N. Harvey, D. M. Murphy, E. C. Neeve, J. Nunn, D. R. Pye, *Chem. Eur. J.* **2014**, *20*, 7935.

[S4] J. Ge, J. Jiang, C. Yuan, C. Zhang, M. Liu, *Tetrahedron Lett.* **2017**, *58*, 1142.

[S5] J. Chen, J. Zhang, D. Zhu, T. Li, *App. Organometal Chem*. **2017**, 3996.

[S6] L. Sevaa , W. Hwang, S. Sabiah, *J. Mol. Catal. A: Chem*. **2016** *418*, 125.

[S7] L. Baia, J. Wang, *Adv. Synth. Catal.* **2008**, 350, 315.

[S8] C. Bai, X. Yao, Y. L, *ACS Catal*. **2015**, *5*, 884.

[S9] T. Wang, L. Liu, K. Xu, H. Xie, H. Shen, W. Zhao, *RSC Adv*. **2016**, *6*, 100690.

[S10] H. G. Ghaleshahi, G. Antonacci, R. M, *Eur. J. Org. Chem*. **2017**, 1331.

[S11] X. Li, W. Chen, H. Chang, Z. Shao, W. We, *Synthesis* **2014**, *46*, 1593.

[S12] Y. Zhao, V. Snieckus, *Org. Lett.* **2014**, *16*, 3200.

[S13] K. Saito, T. Umi, T. Yamada, T. Suga, T. Akiyama, *Org. Biomol. Chem*. **2017**, *15*, 1767.

[S14] N. Zhang, C. Wang, G. Zou, J. Tang, *J. Organomet. Chem.* **2017**, *842*, 54.

[S15] Y. Ye, W. Liu, B. Ye, *Catal. Comm.* **2017**, *89*, 100.

[S16] S. Mattiello, M. Rooney, A. Sanzone, P. Brazzo, M. Sassi, L. Beverina. *Org. Lett*. **2017**, *19*, 654.

[S17] F. Hu, X. Lie, *Tetrahedron* **2014**, *70*, 3854.

[S18] S. Kirchberg, S. Tani, K. Ueda, J. Yamaguchi, A. Studer, K. Itami, *Angew. Chem. Int. Ed*. **2011**, *50*, 2387.

[S19] Z. Fan, K. Wu, L. Xing, Q. Yao, A. Zhang, *Chem. Commun.*, **2014**, *50*, 1682.

[S20] Bruker-AXS SAINT V7.68A Madison, Wisconsin.

[S21] G.M. Sheldrick, SADABS V2008/1, University of Göttingen, Germany

[S22] O.V. Dolomanov, L.J. Bourhis, R.J. Gildea, J.A. K. Howard, H. Puschmann, *J. Appl. Cryst*. **2009**, *42*, 339.

[S23] Sheldrick, G.M. (2015). Acta Cryst. A71, 3.

[S24] Sheldrick, G.M. (2015). Acta Cryst. C71, 3-8.
